# Diastereoselective synthesis of nitroso acetals from (*S*,*E*)-γ-aminated nitroalkenes via multicomponent [4 + 2]/[3 + 2] cycloadditions promoted by LiCl or LiClO_4_

**DOI:** 10.3762/bjoc.9.96

**Published:** 2013-04-30

**Authors:** Leandro Lara de Carvalho, Robert Alan Burrow, Vera Lúcia Patrocinio Pereira

**Affiliations:** 1 Núcleo de Pesquisas de Produtos Naturais, Laboratório de Síntese Estereosseletiva de Substâncias Bioativas, Universidade Federal do Rio de Janeiro, 21941-902, Rio de Janeiro, Brazil; 2 Departamento de Química, Laboratório de Materiais Inorgânicos, Universidade Federal de Santa Maria, 97105-900, Santa Maria, Rio Grande do Sul, RS, Brazil

**Keywords:** chiral heterodiene, hetero-Diels–Alder reaction, pyrrolizidin-3-one, solvent effect, tandem reaction

## Abstract

Chiral nonracemic aminated nitroso acetals were synthesized via diastereoselective multicomponent [4 + 2]/[3 + 2] cycloadditions employing new (*S*,*E*)-γ-nitrogenated nitroalkenes **5a–c** as heterodienes, ethyl vinyl ether (EVE) as a dienophile, and selected electron-deficient alkenes as 1,3-dipolarophiles. The employment of different organic solutions of LiClO_4_ or LiCl as promoter systems provided the respective nitroso acetals with yields from 34–72% and good levels of diastereoselectivity. In addition, the nitroso acetal **9c** was transformed to the pyrrolizidin-3-one derivative **14c**, proving the usefulness of the route in the synthesis of an interesting chiral compound. The elucidation of the stereostructures was based on 2D COSY, NOESY and HSQC NMR experiments as well as an X-ray diffraction experiment.

## Introduction

**Scheme 1:**
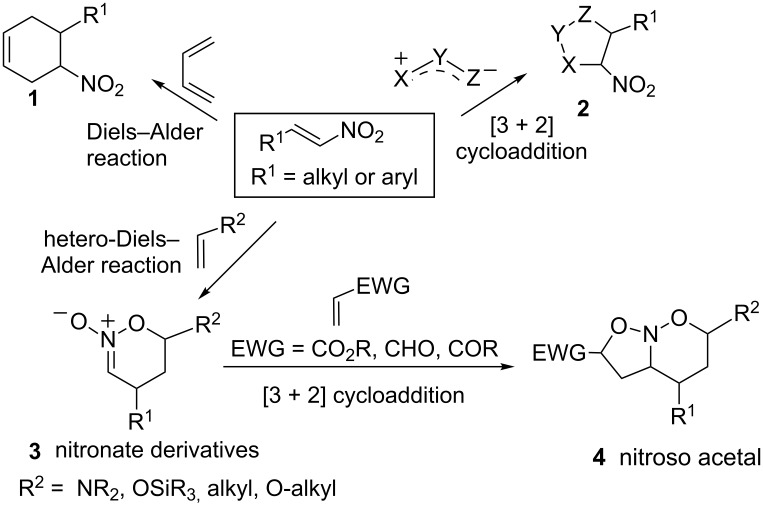
Reactivity of nitroalkenes and/or their respective nitronates in cycloaddition reactions.

Conjugated nitroalkenes play an important role in cycloaddition reactions providing useful nitrogenated cycloadducts with varied synthetic applications (Scheme 1) [1–3]. These compounds can act as dienophiles or 1,3-dipolarophiles to provide nitrocycloadducts of type **1** or nitroheterocycles of type **2**, respectively [4–5]. In addition, nitroalkenes can act as heterodienes reacting with suitable dienophiles, often in the presence of a Lewis acid, to furnish cyclic nitronate derivatives of type **3** [1,3].

In particular, the reactivity of nitroalkenes with unactivated olefins has been extensively investigated by Denmark and co-workers in [4 + 2] hetero-Diels–Alder (HDA) cycloadditions [1,3] and, in some instances, the resulting cyclic nitronates such as **3** (R^2^ = alkyl) were employed in various synthetic transformations [6–7] (Scheme 1). In addition, Denmark’s group and others investigated the tandem [4 + 2]/[3 + 2] nitroalkene cycloaddition employing unactivated olefins or enol ethers as dienophiles and electron-deficient alkenes as 1,3-dipolarophiles to furnish nitroso acetals of type **4** in an inter- or intramolecular fashion [1–3,8–9]. These nitroso acetals can be transformed into functionalized pyrrolizidin-3-ones and in sequence into alkaloid nuclei [1,3,10–11].

The majority of the tandem nitroalkene cycloadditions require the addition of a Lewis acid as a promoter reaction; however, a limited number of these species have been employed in these reactions, e.g., SnCl_4_ or Ti(O-iPr)_2_Cl_2_ [1–3]. However, LiClO_4_ or LiCl solutions have not been used in tandem nitroalkene cycloadditions, although they are widely employed as promoters in Diels–Alder (DA) and HDA reactions [12–17].

Regarding enantioselective processes, the majority of them have been associated with the employment of a specific Lewis acid and a selected chiral inductor connected to the enol ether moiety to furnish nonracemic nitroso acetals diastereoselectively [1–3]. In contrast, the use of a chiral pool strategy, wherein the nitroalkene is the chiral source, is still scarce. To the best of our knowledge, only Chattopadhyaya et al. [18] and Cintas et al. [9] utilized chiral nitroalkenes, synthesized from a nucleoside and a carbohydrate, respectively, to obtain nitroso acetals diastereoselectively.

**Scheme 2:**
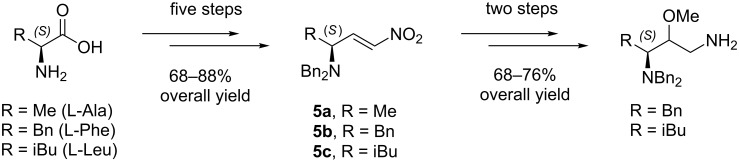
Synthetic route toward the chiral (*S*,*E*)-γ-aminated nitroalkenes **5a–c** and their 1,3-diamine derivatives.

In our continued studies on the reactivity of chiral nonracemic nitro compounds [19–26], we recently synthesized the γ-aminated nitroalkenes **5a–c** from L-alanine, L-phenylalanine and L-leucine, respectively, in five steps and 68–88% overall yield (Scheme 2). These electron-deficient nitroalkenes have exhibited excellent stereochemical stability and reactivity in conjugate additions with varied nucleophiles. The 1,3-nitroamines adducts obtained from these additions can be readily transformed into potentially useful chiral 1,3-diamines [19].

Herein, we report the reactivity and diastereoselectivity of **5a–c** as heterodienes in a multicomponent [4 + 2]/[3 + 2] cycloaddition with ethyl vinyl ether (EVE) and selected electron-deficient alkenes in the presence of LiCl or LiClO_4_ as promoters. A study on the solvent effect was also accomplished. Eleven novel aminated nitroso acetal derivatives were synthesized diastereoselectively, and one of these was transformed into the corresponding pyrrolizidin-3-one derivative to establish the utility of these cycloadducts.

## Results and Discussion

In an exploratory study to screen the best solvent system, the reactivity of **5a**,**b** with EVE and methyl acrylate (MA) as a dipolarophile was evaluated in the absence of a promoter. In all experiments conducted, the cycloadducts were obtained in 18–70% yield with total chemo- and regioselectivity including good levels of diastereoselectivity (Table 1). The nitroso acetal **6a** and another unidentified diastereoisomer were obtained in low yields after long reaction times from **5a** when toluene and dichloromethane were employed as the solvents (Table 1, entries 1 and 2). Similar behavior was observed when THF was used (Table 1, entry 3). The modest solvent performances shown in Table 1, entries 1–3 led us to use more polar solvents.

**Table 1:** Nitroso acetal synthesis via multicomponent [4 + 2]/[3 + 2] cycloadditions of **5a**,**b** with EVE and methyl acrylate in several solvents.

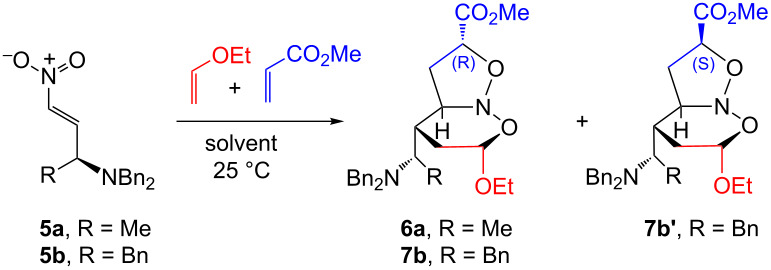						

entry	nitro alkene	solvent	time^a^ (days)	cycloadducts	yield (%)^b^	d.r.^c^

1	**5a**	PhMe	50	**6a/**nd^d^	18 (31)^e^	1.9 : 1.0
2	**5a**	CH_2_Cl_2_	24	**6a/**nd^d^	38 (15)^e^	1.8 : 1.0
3	**5a**	THF	50	**6a/**nd^d^	20 (28)^e^	1.9 : 1.0
4	**5a**	ethanol	20	**6a/**nd^d^	67	1.2 : 1.0
5	**5b**	MeOH	21	**7b/7b’**	70	2.2 : 1.0
6	**5b**	ethanol	25	**7b/7b’**	59	2.1 : 1.0
7	**5b**	2-propanol	30	**7b/7b’**	57 (7)^e^	2.5 : 1.0
8	**5b**	MeOH:H_2_O (3:1)	8	**7b/7b’**	62	2.0 : 1.0
9	**5b**	EtOH:H_2_O (3:1)	7	**7b/7b’**	70	2.4 : 1.0

^a^Monitored by thin layer chromatography. ^b^Purified overall yields of the diastereoisomers mixture. ^c^Diastereoisomeric ratio determined by ^1^H NMR and ^13^C NMR analysis of the crude reaction mixtures. ^d^Diastereoisomer with stereochemistry not defined. ^e^Percentage of nitroalkene not reacted and recovered after purification.

Thus, the use of ethanol resulted in a decrease in the reaction time affording a diastereoisomeric mixture in improved yields (Table 1, entry 4). Similarly, when **5b** was reacted in the presence of methanol (Table 1, entry 5), a higher yield of **7b**,**b’** was achieved compared with ethanol or 2-propanol (Table 1, entries 6 and 7). Next, the use of a more polar medium, such as an alcohol/water mixture 3:1 (Table 1, entries 8 and 9), caused no significant change in the yields in comparison to Table 1, entries 4–7; however, the reaction time decreased considerably. These results demonstrate that the new chiral γ-aminated nitroalkenes **5a**,**b** were reactive in these cycloadditions, even in the absence of a promoter, providing the respective cycloadducts diastereoselectively. In all entries, only two diastereoisomers were obtained among the sixteen possibilities. Additionally, no change in the sense of stereo induction was noted regarding the solvent system employed.

The increase in the reaction rate on employing more polar solvents can be explained through a large stabilization of the dipolar [4 + 2] transition state (TS) [9,27]. Theoretical investigations indicate that the TS of the HDA nitroalkene cycloadditions presents considerable charge transference and a large degree of asynchronicity, but remains a concerted process. In other words, the TS presents a high zwitterionic and polar character without a zwitterionic intermediate specifically. In the second step, the TS involved in [3 + 2] nitronate cycloadditions shows lower charge transference and degree of asynchronicity, and therefore, it is expected that a lower stabilizing effect is caused by polar solvents [9,30–31]. Furthermore, when a fraction of water is present in the medium, the hydrophobic effect can lead the reaction partners to collapse to a TS that is less hydrophobic and less destabilized than the initial state, promoting an increase in the reaction rate [17,28–29].

Aiming to improve the efficiency of the cycloaddition reactions, LiCl or LiClO_4_ solutions were used as a reaction promoter. These salts were chosen in particular because of their present high recyclability, low cost, and great applicability as promoters in cycloaddition processes [12–17]. Thus, the reactions between the very reactive *beta*-nitrostyrene **5d**, used as a model compound, and the chiral nitroalkenes **5a–c** with EVE and MA, acrylonitrile (AN) or methyl vinyl ketone (MVK) were carried out using lithium salt solutions (Table 2). Initially, the reactivity of **5d** in lithium perchlorate solution 4.7 M in THF/H_2_O (3:1) (henceforth LPTW), (Table 2, entry 1) was investigated. A mixture of only three diastereoisomers was obtained in good yield and useful reaction time. When lithium chloride solution 4.7 M in EtOH/H_2_O (3:1) (henceforth LCEW) was employed (Table 2, entry 2), the outcome was similar to that in Table 2, entry 1. Lithium chloride is not appreciably soluble in THF and for this reason ethanol was used as the solvent. The confirmation of the stereostructures of (+/−)-**8d**,**d’** was accomplished by comparison with NMR spectroscopic data available in the literature [9]. Based on these successes, **5a** was reacted with EVE and methyl acrylate in LPTW or LCEW solutions (Table 2, entries 3 and 4), respectively. In these experiments, a mixture of two diastereoisomers was observed in low yield from which only **6a** could be successfully isolated and identified.

**Table 2:** Nitroso acetal synthesis via multicomponent [4 + 2]/[3 + 2] cycloadditions of **5a–d** with EVE and electron-deficient alkenes in the presence of lithium salt solutions.

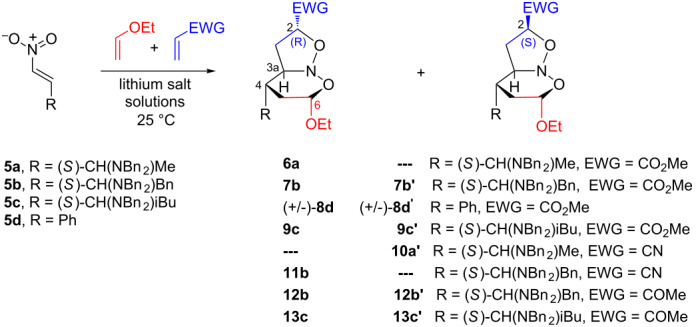						

entry	nitro alkenes	EWG	reaction conditions^a^	time^b^ (days)	cycloadducts (%)^c^	d.r.^d^

1	**5d**	CO_2_Me	LiClO_4_/THF/H_2_O^e^	2.5	**8d/8d’/**nd^f^ (85)^g^	4.0:1.4:1.0
2	**5d**	CO_2_Me	LiCl/EtOH/H_2_O^e^	2.5	**8d**/**8d’**/nd^f^ (77)^g^	3.3:1.3:1.0
3	**5a**	CO_2_Me	LiClO_4/_THF/H_2_O	2	**6a** (21)/nd^f^ (16)	2.3:1.0
4	**5a**	CO_2_Me	LiCl/EtOH/H_2_O	2	**6a** (17)/ nd^f^ (17)	1.7:1.0
5	**5b**	CO_2_Me	LiClO_4_/THF/H_2_O	3	**7b** (51)/**7b’** (21)	2.0:1.0
6	**5b**	CO_2_Me	LiCl/EtOH/H_2_O	3	**7b** (43)/ **7b’** (17)	1.4:1.0
7	**5b**	CO_2_Me	LiClO_4_/EtOH/H_2_O	3	**7b** (47)/ **7b’** (19)	1.7:1.0
8	**5c**	CO_2_Me	LiClO_4_/THF/H_2_O	3	**9c** (35)/ **9c’** (19)	1.6:1.0
9	**5a**	CN	LiClO_4_/THF/H_2_O	1	**10a’** (31)/ nd^f^ (30)	1.0:1.0
10	**5b**	CN	LiClO_4_/THF/H_2_O	3	**11b** (30)/ nd^f^ (23)	1.7:1.0
11	**5b**	COMe	LiClO_4_/THF/H_2_O	3	**12b’** (35)/ **12b** (18)	1.8:1.0
12	**5c**	COMe	LiClO_4_/THF/H_2_O	2	**13c’** (27)/ **13c** (7)	1.5:1.0

^a^Lithium salt solutions 4.7 M in organic solvent/water (3:1). ^b^Monitored by thin-layer chromatography. ^c^Purified by silica-gel column chromatography. ^d^Determined by ^1^H NMR and ^13^C NMR analysis of the crude reaction mixtures. ^e^Reaction carried out at 10 °C. ^f^Diastereoisomer with stereochemistry not defined. ^g^Overall yield of the diastereoisomeric mixture.

**Figure 1:**
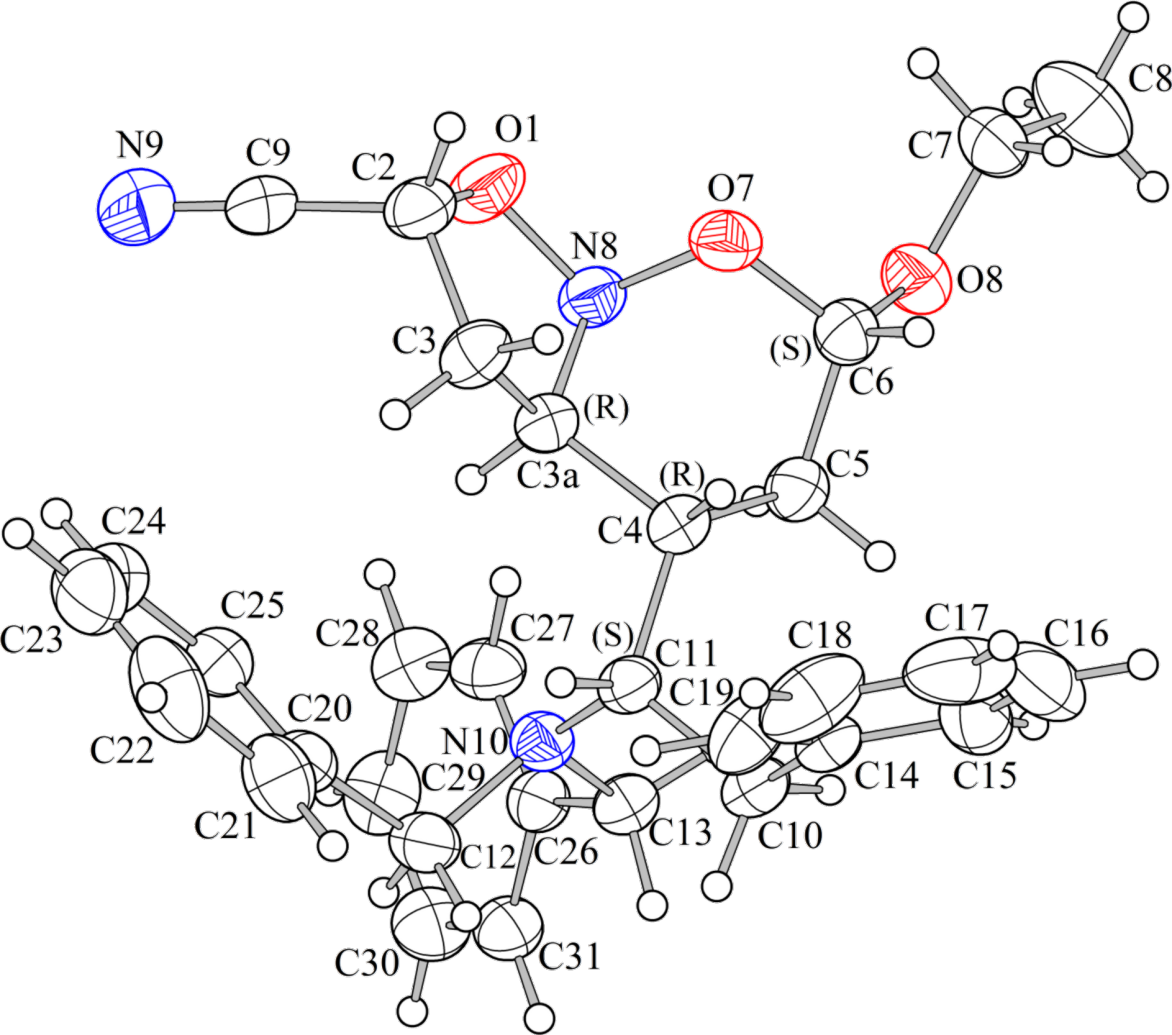
ORTEP for nitroso acetal **11b**.

On the other hand, better yields were achieved employing **5b** or **5c**, EVE and MA to furnish **7b**,**b’** and **9c**,**c’**, respectively, in the presence of LPTW or LCEW or even in lithium perchlorate solution 4.7 M in EtOH/H_2_O (3:1) (Table 2, entries 5–8). In these last entries, good levels of diastereoselectivity were achieved, and the mixture of two diastereoisomers was separated successfully. The reaction of **5a** or **5b** with EVE and AN as a dipolarophile provided a mixture of two diastereoisomers from which only the respective cycloadducts **10a’** and **11b** could be isolated (Table 2, entries 9 and 10). In addition, **11b** was obtained as suitable crystals for an X-ray diffraction experiment, (Figure 1) (Supporting Information File 1). Finally, reaction of **5b** or **5c** with EVE and MVK provided the respective cycloadducts **12b**,**b’** and **13c**,**c’** in low yields and with the same degree of diastereoselectivity (Table 2, entries 11 and 12). In these last two cases, the major diastereoisomers presented the (*S*)-configuration in the CH(2) stereogenic center resulting from the *endo* approach of the smaller dipolarophile MVK by the *Re* face of the nitronate. It is worthwhile to mention that, depending of the nitroso acetal structure, an opening of the six-membered ring is caused due to a greater sensitivity to the reaction medium, leading to generation of the respective carbonylated isoxazolines and, therefore, causing a decrease in yield. These last compounds could be identified through ^1^H and ^13^C NMR spectroscopy. The signals at 9.5 ppm, and 201 and 159 ppm revealed the presence of an aldehyde function and a sp^2^ carbon bond of an isoxazoline ring (spectra not related).

The cycloadditions with **5a–c** were performed in concentrated lithium salt solutions following similar protocols described in the literature in which lithium perchlorate 5.0 M in diethyl ether (LPDE) was employed successful as a promoter in Diels–Alder reactions. This solution was found to be a good reaction medium causing a large enhancement in the reaction rate. Moreover, the role of the lithium salt solution in the cycloaddition reactions is still controversial and has been the subject of discussion. The increase of the solvent internal pressure, caused by the presence of lithium salt, and the Lewis acid catalysis by cation–substrate interaction have been utilized to explain this enhancement of the reaction rate [12–14].

In the case of nitroalkene cycloadditions, performed in the presence of a Lewis acid, it is well documented that the rate acceleration effect caused by the Lewis acid in the [4 + 2] step is due to lowering of the LUMO energy of the nitroalkene [1,6,32–33]. Follow this judgment, we propose that the lithium cation could bind to one of the oxygen atoms of the nitro group, acting as a Lewis acid, to promote the rate acceleration of the cycloadditions. In the [3 + 2]-step the role of the Lewis acid is not well established; however, it is reasonable to imagine that lithium can be transferred from the nitronate to the electron-withdrawing group (EWG) of the 1,3-dipolarophile causing a decrease in the activation energy of this step, as similarly proposed by Domingos and co-workers in a PM3 study on domino reactions with nitroalkenes [34]. Additionally, it is possible that the high internal pressure of the solvent acts synergistically to promote the enhancement of the reaction rate of **5a–c** and should not be neglected.

**Figure 2:**
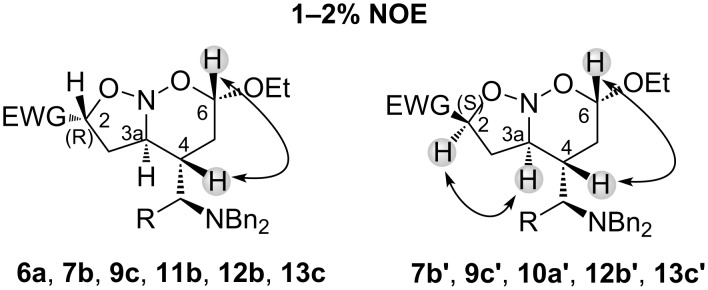
1D NOESY correlation between H2, H3a, H4 and H6 for all nitroso acetals.

In all cycloadditions, independent of the lithium salt/solvent system employed, no change was observed in the sense of diastereoselection. All stereostructures were elucidated from IR, ^1^H NMR, ^13^C NMR, 2D COSY, HSQC and 2D NOESY experiments (Supporting Information File 2). The absolute configurations were assigned from 2D NOESY experiments and could be corroborated by X-ray analysis of **11b**, since the diastereoselection of the [4 + 2]-step was the same for all cycloadditions investigated (Figure 1 and Figure 2).

The determination of the vicinal H,H constant coupling of the protons located in the six-membered ring was not so simple due to the tendency of these frameworks to assume the twist-boat conformation, as shown in X-ray analysis of **11b** (Figure 1). According to ^1^H NMR analysis of all cycloadducts, the protons (H6) showed the highest vicinal *J*-coupling in the range of 7.3 Hz to 8.0 Hz suggesting a pseudoequatorial arrangement [35]. The tendency to the pseudoaxial orientation of the alkoxy group in nitroso acetals is associated with a stabilization generated by an anomeric effect [1,36]. The protons (H6) and (H4) were both irradiated in 2D NOESY experiments and a *cis* relationship between them was achieved for all cycloadducts. When the stereogenic center at HC(2) presents (*S*)-configuration, as in **7b’**, **9c’**, **10a’**, **12b’** and **13c’**, the 2D NOESY experiments showed a *cis* relationship between (H2) and (H3a). In addition, the range of the vicinal H,H constant coupling of (H2) spans from 8.3 Hz to 10 Hz for all cycloadducts, and these high values suggest the orientation of EWG close to the equatorial position.

**Scheme 3:**
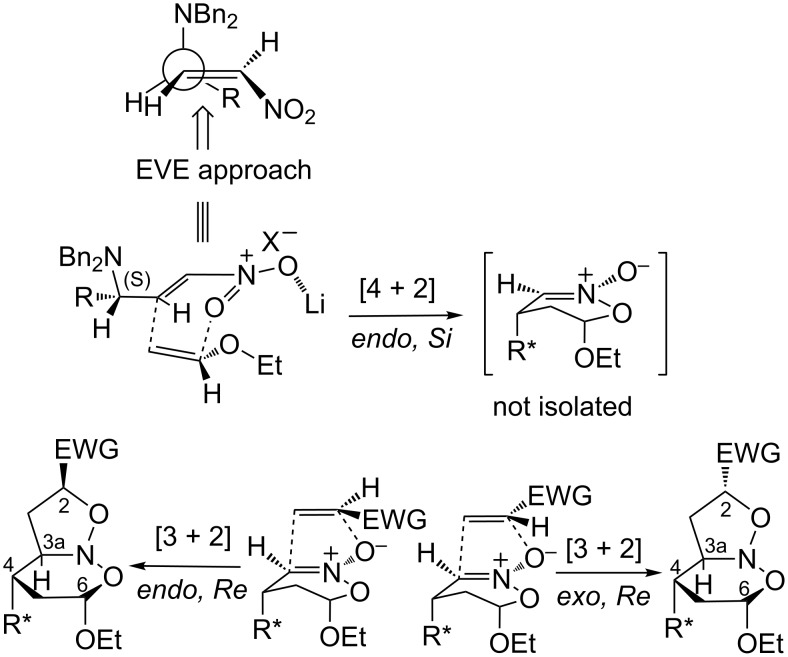
Transition-state models to stereoselective approaches in the multicomponent cycloadditions of **5a**–**c**.

In the [4 + 2] cycloadditions, the total facial diastereoselection exhibited by **5a–c** could be rationalized by a modified Felkin–Anh TS model [37–40] in which the largest *N,N*-dibenzylamino group is orthogonal to the electron-deficient C=C bond (Scheme 3). Thus, the approach of the enol ether to the β-nitro carbon was preferred by the less hindered *Si* face on the opposite side to the largest group. Secondary orbital and Coulombic interactions have been proposed to explain the *endo* approach of the enol ethers [9,33,41].

In the [3 + 2] cycloadditions, we believe that in the TS the respective nitronates present a half-chair conformation with the HC(4) substituent and the alkoxy group in an axial position to maximize the stabilization generated by the anomeric effect [9,33,41–42]. Thus, the competitive *endo*/*exo* approach of the 1,3-dipolarophile occurred by the *Re* face on the opposite side to the bulky lateral chain at HC(4).

**Scheme 4:**
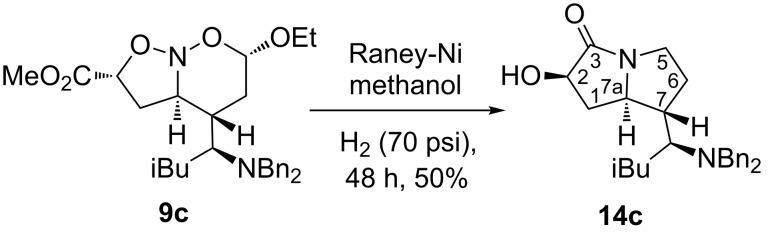
Hydrogenolysis of **9c** to pyrrolizidin-3-one **14c**.

In order to prove the applicability of the aminated nitroso acetals, the N–O bonds contained in **9c** were easily cleaved under hydrogenolysis conditions to give the corresponding pyrrolizidin-3-one **14c** in 50% yield (Scheme 4). Pyrrolizidin-3-one frameworks are important precursors of pyrrolizidine nuclei, which are largely widespread in nature, mainly in the form of pyrrolizidine alkaloids [43].

## Conclusion

In summary, the multicomponent [4 + 2]/[3 + 2] cycloadditions using a new class of chiral γ-aminated nitroalkenes **5a–c** showed good reactivity, chemo-, regio- and facial diastereoselection. In the absence of lithium salt solutions, polar solvents such as EtOH or MeOH and the binary solvent systems EtOH/H_2_O or MeOH/H_2_O (3:1) were more effective in promoting the reaction in 7–8 days with 60–70% yield. The employment of lithium salt solutions as the reaction medium decreased significantly the reaction time to 1–3 days with similar overall yields (34–72%). In addition, the aminated nitroso acetal **9c** was smoothly hydrogenolyzed to the correspondent pyrrolizidin-3-one **14c** in 50% yield. The route led to the obtainment of a great number of new chiral aminated nitroso acetals (**7**, **9–13**) and the feasible synthesis of the pyrrolizidin-3-one scaffold. The good reactivity and diastereoselectivity of the new chiral-γ-aminated nitroalkenes **5a–c** in multicomponent [4 + 2]/[3 + 2] cycloaddition besides conjugate addition [19] make them useful chiral building blocks for diastereoselective synthesis.

## Experimental

### General

EtOH, MeOH, 2-propanol, toluene, LiClO_4_, LiCl, methyl vinyl ketone, ethyl vinyl ether, methyl acrylate and acrylonitrile were purchased from Aldrich, Acros or Merck and were used as received. CH_2_Cl_2_ was dried from CaH_2_, and THF was dried according to a literature procedure [44]. Melting points are uncorrected and were determined on a Thomas Hoover apparatus. Optical rotations were recorded at 25 °C using a Jasco P-2000 (PTC-203) polarimeter. The sealed glass tube employed had dimensions of 2.0 cm ø × 15.0 cm. The overall cycloadditions were monitored by thin-layer chromatography (silica gel 60 F_254_ Merck^®^ twice eluted with ethyl acetate/hexane 1:4 v/v) and the visualization was achieved by using iodine impregnated on silica gel or UV light (254 nm). Liquid chromatography was performed on columns of silica gel 60 (70–230 mesh) and eluted with ethyl acetate/hexane gradient (5–15% v/v). IR spectra were recorded on a Shimadzu FT-IR spectrophotometer as a film on a NaCl plate. ^1^H NMR and ^13^C NMR spectra were recorded on a Varian or Bruker spectrometer operating at (400 or 500 MHz) and (100 or 125 MHz), at 25 °C by using CDCl_3_ 0.5% TMS v/v as solvent. HRMS (ESI) experiments were performed in positive mode on a Bruker Daltonics ultrOTOFQ-ESI-TOF mass spectrometer.

## Supporting Information

File 1 Experimental section and characterization for **6a**, **7b**,**b’**, **9c**,**c’**, **10a’**, **11b**, **12b**,**b’**, **13c**,**c’** and **14c**. Available edited spectra of IR, ^1^H NMR, ^13^C NMR, 2D COSY, HSQC and 2D NOESY.

File 2 Dataset of X*-*ray crystallography and extended ORTEP drawing of **11b**.
