# Supplementary material for: Diastereoselective synthesis of nitroso acetals from (S,E)-γ-aminated nitroalkenes via multicomponent [4 + 2]/[3 + 2] cycloadditions promoted by LiCl or LiClO4
Source: Beilstein J Org Chem. 2013 Apr 30;9:838–45. doi: 10.3762/bjoc.9.96 (PMC3678848; doi:10.3762/bjoc.9.96)
Supplement: File 1 — Experimental section and characterization for 6a, 7b,b’, 9c,c’, 10a’, 11b, 12b,b’, 13c,c’ and 14c. Available edited spectra of IR, 1H NMR, 13C NMR, 2D COSY, HSQC and 2D NOESY. [file Beilstein_J_Org_Chem-09-838-s001.pdf]

# SUPPORTING INFORMATION

**for**

# Diastereoselective synthesis of nitroso acetals from (*S*,*E*)-γ-aminated nitroalkenes via multicomponent [4 + 2]/[3 + 2] cycloadditions promoted by LiCl or $LiClO_4$

**Leandro Lara de Carvalho[1], Robert Alan Burrow[2] and Vera Lúcia Patrocinio Pereira*[1,§]**

Address: [1]Núcleo de Pesquisas de Produtos Naturais, Laboratório de Síntese Estereosseletiva de Substâncias Bioativas, Universidade Federal do Rio de Janeiro, 21941-902, Rio de Janeiro, Brazil and [2]Departamento de Química, Laboratório de Materiais Inorgânicos, Universidade Federal de Santa Maria, 97105-900, Santa Maria, Rio Grande do Sul, RS, Brazil

Email: Vera Lúcia Patrocinio Pereira - patrocinio@correio.nppn.ufrj.br

[*]Corresponding author

[§]Tel.: +55 21 2562 6792; Fax: +55 21 2562 6512

## Experimental section and characterization for 6a, 7b,b’, 9c,c’, 10a’, 11b, 12b,b’, 13c,c’ and 14c. Available edited spectra of IR, $^{1}$H NMR, $^{13}$C NMR, 2D COSY, HSQC and 2D NOESY

**1 Experimental section and characterization**......S4
Spectrum 1 – Infrared of **6a**......S10
Spectrum 2 – $^{1}$H and $^{13}$C NMR of **6a**......S11
Spectrum 3 – 2D COSY of **6a**......S12
Spectrum 4 – HSQC of **6a**......S13
Spectrum 5 – 2D NOESY of **6a**......S14
Spectrum 6 – Infrared of **7b**......S15
Spectrum 7 - $^{1}$H and $^{13}$C NMR of **7b**......S16
Spectrum 8 – 2D COSY of **7b**......S17
Spectrum 9 – HSQC of **7b**......S18
Spectrum 10 – 2D NOESY of **7b**......S19

Spectrum 11 – Infrared of **7b'** ........ S20
Spectrum 12 - $^{1}$H and $^{13}$C NMR of **7b'** ........ S21
Spectrum 13 – 2D COSY of **7b'** ........ S22
Spectrum 14 – 2D NOESY of **7b'** ........ S23
Spectrum 15 – Infrared of **9c** ........ S24
Spectrum 16 - $^{1}$H and $^{13}$C NMR of **9c** ........ S25
Spectrum 17 – 2D COSY of **9c** ........ S26
Spectrum 18 – HSQC of **9c** ........ S27
Spectrum 19 – 2D NOESY of **9c** ........ S28
Spectrum 20 – Infrared of **9c'** ........ S29
Spectrum 21 - $^{1}$H and $^{13}$C NMR of **9c'** ........ S30
Spectrum 22 – 2D COSY of **9c'** ........ S31
Spectrum 23 – HSQC of **9c'** ........ S32
Spectrum 24 – 2D NOESY of **9c'** ........ S33
Spectrum 25 – Infrared of **10a'** ........ S34
Spectrum 26 - $^{1}$H and $^{13}$C NMR of **10a'** ........ S35
Spectrum 27 – 2D COSY of **10a'** ........ S36
Spectrum 28 – HSQC of **10a'** ........ S37
Spectrum 29 – 2D NOESY of **10a'** ........ S38
Spectrum 30 – Infrared of **11b** ........ S39
Spectrum 31 - $^{1}$H and $^{13}$C NMR of **11b** ........ S40
Spectrum 32 – 2D COSY of **11b** ........ S41
Spectrum 33 – HSQC of **11b** ........ S42
Spectrum 34 – 2D NOESY of **11b** ........ S43
Spectrum 35 – Infrared of **12b** ........ S44
Spectrum 36 - $^{1}$H and $^{13}$C NMR of **12b** ........ S45
Spectrum 37 – 2D COSY of **12b** ........ S46
Spectrum 38 – HSQC of **12b** ........ S47
Spectrum 39 – 2D NOESY of **12b** ........ S48
Spectrum 40 – Infrared of **12b'** ........ S49
Spectrum 41 - $^{1}$H and $^{13}$C NMR of **12b'** ........ S50
Spectrum 42 – 2D COSY of **12b'** ........ S51
Spectrum 43 – HSQC of **12b'** ........ S52
Spectrum 44 – 2D NOESY of **12b'** ........ S53
Spectrum 45 – Infrared of **13c** ........ S54
Spectrum 46 - $^{1}$H and $^{13}$C NMR of **13c** ........ S55
Spectrum 47 – 2D COSY of **13c** ........ S56
Spectrum 48 – HSQC of **13c** ........ S57
Spectrum 49 – 2D NOESY of **13c** ........ S58

Spectrum 50 – Infrared of **13c’** ........................................................ S59
Spectrum 51 - $^{1}$H and $^{13}$C NMR of **13c’** ........................................................ S60
Spectrum 52 – 2D COSY of **13c’** ........................................................ S61
Spectrum 53 – 2D NOESY of **13c’** ........................................................ S62
Spectrum 54 – Infrared of **14c** ........................................................ S63
Spectrum 55 - $^{1}$H and $^{13}$C NMR of **14c** ........................................................ S64
Spectrum 56 – 2D COSY of **14c** ........................................................ S65
Spectrum 57 – 2D NOESY of **14c** ........................................................ S67

## 1 EXPERIMENTAL SECTION AND CHARACTERIZATION

Typical procedure 1 - Multicomponent [4 + 2]/[3 + 2] cycloadditions performed without lithium salt solution (Table 1, entry 4).

**(1'*S*,2*R*,3a*R*,4*R*,6*S*)-4-(1'-*N,N*-Dibenzylaminoethyl)-6-ethoxy-2-methoxycarbonylperhydro-isoxazolidino[2,3-*b*]-1,2-oxazine (6a)**: To a solution of **5a** (85.0 mg, 0.287 mmol) in ethanol (3.75 mL) were added EVE (0.69 mL, ~25 equiv) and methyl acrylate (0.64 mL, ~25 equiv). The reaction mixture was kept in a sealed glass tube at 25 °C and under constant stirring for 20 days. After, the organic solution was evaporated to dryness under reduced pressure at 25 °C and the resulting crude oil was purified rapidly by chromatography on a short silica-gel column using ethyl acetate-hexane (15% v/v) as eluent. A mixture of two diastereoisomers was obtained in 67% yield (d.r. = 1.2:1.0) where **6a** is majority diastereoisomer. Pale yellow oil. $[\alpha]_D^{25}$ = +27.1° (*c* 1.16, $CHCl_3$); IR (film) 3061–2804, 1746 (C=O), 1448, 1375, 1221, 1107, 1066, 1026 (N-O), 929, 744 $cm^{-1}$; $^1H$ NMR (400 MHz, $CDCl_3$) δ ppm 7.34-7.20 (m, 10H), 4.99 (dd, *J* = 9.9, 4.2 Hz, 1H, HC(2)), 4.73 (dd, *J* = 7.9, 7.1 Hz, 1H, HC(6)), 3.91 (dq, *J* = 9.5, 7.1 Hz, 1H), 3.76 (d, *J*=13.7 Hz, 2H), 3.74 (s, 3H), 3.51 (dq, *J* = 9.5, 7.1 Hz, 1H), 3.32 (d, *J* = 13.7 Hz, 2H), 3.25 (m, 1H, HC(3a)), 2.69 (ddd, *J* = 13.2, 9.5, 6.6 Hz, 1H), 2.55 (ddd, *J* = 13.8, 6.8, 3.3 Hz, 1H), 2.38 (ddd, *J* =12.2, 7.9, 4.3 Hz, 1H), 2.27 (ddd, *J* = 12.3, 9.6, 9.6 Hz, 1H), 1.75 (m, 1H, HC(4)), 1.55 (ddd, *J*=13.67, 13,67, 8.38 Hz, 1H), 1.25 (t, *J* = 7.1 Hz, 3H), 1.05 (d, *J* = 6.6 Hz, 3H); $^{13}C$ NMR (100 MHz, $CDCl_3$) δ ppm 170.6, 139.4, 128.7, 128.3, 127,0 100.5, 80.8, 72.0, 63.1, 55.4, 53.8, 52.4, 42.5, 36.7, 29.5, 15.1, 9.8; HRMS (ESI) calcd. for $C_{26}H_{35}N_2O_5$ $[M + H]^+$ 455.2546 found 455.2541.

Typical procedure 2 - Multicomponent [4 + 2]/[3 + 2] cycloadditions promoted by lithium salt solutions (Table 2, entry 5).

**(1'*S*,2*R*,3a*R*,4*R*,6*S*)-4-(1'-*N,N*-Dibenzylamino-2'-phenylethyl)-6-ethoxy-2-methoxycarbonylperhydro-isoxazolidino[2,3-*b*]-1,2-oxazine (7b) and (1'*S*,2*S*,3a*R*,4*R*,6*S*)-4-(1'-*N,N*-dibenzylamino-2'-phenylethyl)-6-ethoxy-2-methoxycarbonylperhydro-isoxazolidino[2,3-*b*]-1,2-oxazine (7b'):** In a sealed glass tube **5b** (154.4 mg, 0.414 mmol) was dissolved in $LiClO_4$ solution 4.0 M in anhydrous THF (4.13 mL), and EVE (0.99 mL, ~25 equiv) and methyl acrylate (0.93 mL, ~25 equiv) were added under constant stirring. Next, $LiClO_4$ solution 6.84 M in water (1.26 mL) was slowly dripped into the reaction medium, in order to keep it homogeneous and free from solid particles. The reaction mixture was kept at 25 °C and under constant stirring for 3 days. Afterward, dichloromethane (15 mL) was added to the reaction medium, precipitating the lithium salt in the solution. The heterogenic mixture was filtered and washed with DCM to extract the desired product. The organic solution was reserved and the recovered lithium salt was contained in a round-bottom flask and maintained under reduced pressure at 25 °C for 5 hours. After this procedure the recycled salt could be reused. The reserved organic solution was directly evaporated to dryness under reduced pressure at 25 °C to avoid decomposition. The resulting crude oil was purified carefully by chromatography on a silica-gel column using ethyl acetate-hexane gradient (5–15% v/v) to furnish **7a** (112.0 mg) in 51% yield and **7b** (46.1 mg) in 21% yield. Compound **7b**: Pale yellow oil. $[\alpha]_D^{25}$ = +100.8° (*c* 1.01, $CHCl_3$); IR (film) 3084–2802, 1743 (C=O), 1494, 1454, 1209, 1109, 1058, 1028 (N-O), 929 $cm^{-1}$; $^1H$ NMR (400 MHz, $CDCl_3$) δ ppm 7.36-7.11 (m, 15H), 4.78 (dd, *J* = 10.1, 5.0 Hz, 1H, HC(2)), 4.62 (dd, *J* = 7.9, 6.8 Hz, 1H, HC(6)), 3.89 (m, 1H), 3.86 (d, *J* = 14.0 Hz, 2H), 3.76 (m, 1H, HC(3a)), 3.73 (s, 3H), 3.46 (m, 1H), 3.41 (d, *J* = 13.4 Hz, 2H), 3.21 (dd, *J* = 13.5, 4.2 Hz, 1H), 2.93 (m, 1H), 2.68 (dd, *J* = 13.3, 10.4 Hz, 1H), 2.22 (ddd, *J* = 12.8, 6.4, 2.4 Hz, 1H), 1.88 (ddd, *J* = 13.5, 13.5, 8.4 Hz, 1 H), 1.54 (m, 1H), 1.47 (m, 1H, HC(4)), 1.32 (m, 1H), 1.21 (t, *J* = 7.0 Hz, 3H); $^{13}C$ NMR (100 MHz, $CDCl_3$) δ ppm 170.3, 139.8, 139.3, 129.1-126.2, 100.3, 80.5, 70.5, 63.2, 59.2, 55.5, 52.1, 40.3, 35.1, 31.5, 27.7, 15.0; HRMS (ESI) calcd. for $C_{32}H_{39}N_2O_5$ $[M + H]^+$ 531,2859 found 531.2854. Compound **7b'**: Pale yellow oil. $[\alpha]_D^{25}$ = +80.2° (*c* 2.33, $CHCl_3$); IR (film) 3084–2802, 1737 (C=O), 1492, 1454, 1217, 1209, 1105, 1056, 1030 (N-O), 914 $cm^{-1}$; $^1H$ NMR (400 MHz, $CDCl_3$) δ ppm 7.33-7.13 (m, 15H), 4.63 (dd, *J* = 7.44, 7.21 Hz, 1 H, HC(6)), 4.38 (t, *J* = 8.4 Hz, 1H, HC(2)),

3.93 (m, 1H), 3.85 (d, *J* = 13.3 Hz, 2H), 3.67 (s, 3H), 3.57 (m, 1H, HC(3a)), 3.47 (m, 1H), 3.42 (d, *J* = 13.7 Hz, 2H), 3.22 (dd, *J* = 13.6, 4.1 Hz, 1H), 2.97 (m, 1H), 2.70 ( dd, *J* = 13.4, 10.0 Hz, 1H), 2.21 (m, 1H), 1.86 (m, 1H), 1.69 (m, 1H), 1.56 (m, 1H, HC(4)), 1.42 (m, 1H), 1.21 (t, *J* = 7.0 Hz, 3H)); $^{13}$C NMR (100 MHz, $CDCl_3$) δ ppm 171.2, 139.9, 139.5, 129.2-126.3, 100.5, 81.5, 71.1, 63.3, 59.3, 55.5, 52.3, 40.1, 34.0, 31.9, 27.9, 15.0; HRMS (ESI) calcd. for $C_{32}H_{39}N_2O_5$ $[M + H]^+$ 531.2859 found 531.2878.

**(1'*S*,2*R*,3a*R*,4*R*,6*S*)-4-(1'-*N*,*N*-dibenzylamino-3'-methylbutyl)-6-ethoxy-2-methoxycarbonylperhydro-isoxazolidino[2,3-*b*]-1,2-oxazine (9c) and (1'*S*,2*S*,3a*R*,4*R*,6*S*)-4-(1'-*N*,*N*-dibenzylamino-3'-methylbutyl)-6-ethoxy-2-methoxycarbonylperhydroisoxazolidino[2,3-*b*]-1,2-oxazine** (**9c'**). Nitroalkene **5c** (235.7 mg, 0.696 mmol) was reacted with EVE (1.66 mL, ~25 equiv) and methyl acrylate (1.56 mL, ~25 equiv) for 3 days following the typical procedure 2. Compound **9c**: Yield = 121.0 mg (35%) as pale yellow oil. $[\alpha]_D^{25}$ = +17.3° (*c* 2.50, $CHCl_3$); IR (film) 3084–2802, 1743 (C=O), 1493, 1452, 1371, 1111, 1058, 1028 (N-O), 914 $cm^{-1}$; $^1$H NMR (400 MHz, $CDCl_3$) δ ppm 7.33-7.19 (m, 10H), 4.93 (dd, *J* = 10.0, 4.9 Hz, 1H, HC(2)), 4.72 (dd, *J* = 8.0, 6.7 Hz, 1 H, HC(6)), 3.92 (dq, *J* = 9.6, 7.1, 1 H), 3.78 (s, 3H), 3.76 (m, 1H, HC(3a)), 3.72 (d, *J* = 13.4 Hz, 2H), 3.48 (dq, *J* = 9.6, 7.0, 1 H), 3.36 (d, *J* = 13.4 Hz, 2H), 2.70 (m, 1H), 2.06 (ddd, *J* = 12.9, 6.6, 2.8 Hz, 1H), 1.87 (m, 2 H), 1.64 (m, 1H, HC(4)), 1.54 (m, 3H), 1.42 (m, 1H), 1.21 (t, *J* = 7.1 Hz, 3H), 0.93 (d, *J* = 6.11 Hz, 3 H); 0.89 (d, *J* = 6.11 Hz, 3 H); $^{13}$C NMR (100 MHz, $CDCl_3$) δ ppm 170.4, 139.7, 129.1, 128.3, 126.9, 100.5, 80.6, 70.6, 63.2, 55.3, 54.8, 52.2, 41.0, 35.5, 34.5, 27.9, 25.4, 23.7, 21.9, 15.0. Compound **9c'**: Yield = 65.7 mg (19%). Pale yellow oil. $[\alpha]_D^{25}$ = +61.2° (*c* 1.50, $CHCl_3$); IR (film) 3084–2800, 1737 (C=O), 1494, 1454, 1371, 1219, 1109, 1058, 1028 (N-O), 914 $cm^{-1}$; $^1$H NMR (400 MHz, $CDCl_3$) δ ppm 7.32-7.21 (m, 10H), 4.70 (dd, *J* = 7.9, 6.6 Hz, 1H, HC(6)), 4.49 (dd, *J* = 9.0, 7.1 Hz, 1H , HC(2)), 3.93 (dq, *J* = 9.7, 7.1 Hz, 1H), 3.75 (s, 3H), 3.71 (d, *J* = 13.5 Hz, 2H), 3.63 (m, 1H, HC(3a)), 3.47 (dq, *J* = 9.7, 7.1 Hz, 1H), 3.37 (d, *J* = 13.5 Hz, 2H), 2.71 (m, 1H), 2.04 (ddd, *J* = 12.7, 6.5, 2.4 Hz, 1H), 1.98 (m, 1H), 1.81 (m, 1H), 1.69 (m, 1H, HC(4)), 1.56 (m, 3H), 1.42 (m, 1H), 1.21 (t, *J* = 7.1 Hz, 3H), 0.92 (d, *J* = 5.1 Hz, 3H), 0.91 (d, *J* = 5.3 Hz, 3H); $^{13}$C NMR (100 MHz, $CDCl_3$) δ ppm 171.5, 139.9, 129.2-127.1, 100.6, 81.5, 71.3, 63.4, 55.4, 54.9, 52.4, 40.7, 34.8, 34.1, 28.1, 25.5, 23.8, 22.0, 15.0; HRMS (ESI) calcd. for $C_{29}H_{41}N_2O_5$ $[M + H]^+$ 497.3015 found 497.3012.

**(1'*S*,2*S*,3a*R*,4*R*,6*S*)-4-(1'-*N*,*N*-dibenzylaminoethyl)-2-cyano-6-ethoxyperhydroisoxazolidino[2,3-*b*]-1,2-oxazine (10a').** Nitroalkene **5a** (141.9 mg, 0.479 mmol) was reacted with EVE (1.15 mL, ~25 equiv) and acrylonitrile (0.79 mL, ~25 equiv) for 1 day following the typical procedure 2. Yield = 63.2 mg (31%) as a yellow pale oil. $[\alpha]_D^{25}$ = +49.4° (*c* 1.55, $CHCl_3$); IR (film) 3061–2804, 1492, 1452, 1377, 1211, 1107, 1057, 1022 (N-O), 914 $cm^{-1}$; $^1H$ NMR (400 MHz, $CDCl_3$) δ ppm 7.34-7.21 (m, 10H), 4.81 (m, 2H, HC(6,2)), 3.95 (dq, *J* = 9.7, 7.1 Hz, 1H), 3.75 (d, *J* = 13.7 Hz, 2H), 3.56 (dq, *J* = 9.7, 7.1 Hz, 1H), 3.32 (d, *J* = 13.5 Hz, 2H), 3.23 (m, 1H, HC(3a)), 2.70 (m, 1H), 2.52 (m, 2H), 2.32 (ddd, *J* = 12.3, 9.1, 6.6 Hz, 1H), 1.83 (m, 1H, HC(4)), 1.55 (*J* = 13.7, 13.7, 8.4 Hz, 1H), 1.26 (t, 6.9 Hz, 3H), 1.05 (d, 6.6 Hz, 3H); $^{13}C$ NMR (100 MHz, $CDCl_3$) δ ppm 139.3, 128.8-127.1, 118.6, 100.7, 72.9, 68.8, 63.6, 55.0, 54.0, 42.8, 38.4, 29.3, 15.0, 9.9; HRMS (ESI) calcd. for $C_{25}H_{32}N_3O_3$ $[M + H]^+$ 422.24437 found 422.2437.

**(1'*S*,2*R*,3a*R*,4*R*,6*S*)-4-(1'-*N*,*N*-dibenzylamino-2'-phenylethyl)-2-cyano-6-ethoxyperhydroisoxazolidino-[2,3-*b*]-1,2-oxazine (11b).** Nitroalkene **5b** (132.0 mg, 0.354 mmol) was reacted with EVE (0.84 mL, ~25 equiv) and acrylonitrile (0.58 mL, ~25 equiv) for 3 days following the typical procedure 2. Yield = 52.9 mg (30%). White crystalline solid. Mp = 183–185 °C. For X-ray experiments only 10 mg of the purified material was recrystallized in DCM in a small assay tube, which was kept in a closed flask containing pentane for 7 days at 15 °C. $[\alpha]_D^{25}$ = +75.5° (*c* 1.78, $CHCl_3$); IR (film) 3086–2806, 1494, 1452, 1371, 1271, 1217, 1107, 1078, 1030 (N-O), 933 $cm^{-1}$; $^1H$ NMR (500 MHz, $CDCl_3$) δ ppm 7.41-7.13 (m, 15H), 4.83 (dd, *J* = 9.8, 4.9 Hz, 1H, HC(2)), 4.58 (dd, *J* = 8.3, 6.6 Hz, 1H, HC(6)), 3.86 (m, 4H, HC(3a), $NC\underline{H_2}$-Ph, $CH_3\underline{H}CH$-O), 3.44 (m, 1H), 3.43 (d, *J* = 13.5 Hz, 2H), 3.25 (dd, *J* = 13.2, 3,9 Hz , 1H), 2.92 (ddd, *J* = 10.76, 3.91, 1 H), 2.70 (dd, *J* = 13.3, 10.9 Hz, 1H), 2.19 (ddd, *J* = 13.0, 6.5, 2.6 Hz, 1H ), 1.88 (ddd, *J* = 13.6, 13.6, 8.4 Hz, 1H), 1.48 (ddd, *J* = 12.04, 9.38, 9.38 Hz, 1 H), 1.38 (m, 1H, HC(4)), 1.26 (ddd, *J* = 12.4, 8.4, 4.9 Hz, 1H), 1.21 (t, *J* = 7.1 Hz, 3H); $^{13}C$ NMR (125 MHz, $CDCl_3$) δ ppm 139.7, 139.2, 129.2-126.5, 116.6, 100.7, 71.4, 69.0, 63.7, 58.8, 55.8, 40.0, 36.0, 31.4, 27.2, 15.0; HRMS (ESI) calcd. for $C_{31}H_{36}N_3O_3$ $[M + H]^+$ 498.2757 found 498.2748

**(1'*S*,2*R*,3a*R*,4*R*,6*S*)-2-acetyl-4-(1'-*N*,*N*-dibenzylamino-2'-phenylethyl)-6-ethoxyperhydroisoxazolidino-[2,3-*b*]-1,2-oxazine (12b) and (1'*S*,2*S*,3a*R*,4*R*,6*S*)-2-acetyl-4-(1'-*N*,*N*-dibenzylamino-2'-phenylethyl)-6-ethoxyperhydroisoxazolidino[2,3-*b*]-1,2-oxazine (12b').** Nitroalkene **5b** (117.8 mg, 0.316 mmol) was

reacted with EVE (0.75 mL, ~25 equiv) and methyl vinyl ketone (0.65 mL, ~25 equiv) for 3 days following the typical procedure 2. Compound **12b**: Yield = 30.0 mg (18%) as pale yellow oil. $[\alpha]_D^{25}$ = +41.2° (*c* 1.65, $CHCl_3$); IR (film) 3082–2804, 1720 (C=O), 1492, 1454, 1357, 1107, 1058, 1033 (N-O), 923 $cm^{-1}$; $^1H$ NMR (400 MHz, $CDCl_3$) δ ppm 7.37-7.13 (m, 15H), 4.64 (dd, *J* = 8.0, 6.7 Hz, 1H, HC(6)), 4.61 (dd, *J* = 10.5, 5.6 Hz, 1H, HC(2)), 3.91 (m, 1H), 3.87 (d, *J* = 13.9 Hz, 2H), 3.54 (m, 1H, HC(3a)), 3.47 (ddd, *J* = 7.1, 7.1, 2.4 Hz, 1 H), 3.42 (d, *J* = 13.5 Hz, 2H), 3.25 (dd, *J* = 13.5, 3.7 Hz, 1H), 2.93 (ddd, *J* = 10.7, 3.9, 3.7 Hz, 1H), 2.69 (dd, *J* = 13.5, 10.8 Hz, 1H), 2.22 (ddd, *J*=12.84, 6.23, 2.98 Hz, 1 H), 2.06 (s, 3H), 1.86 (m, 1H), 1.47 (m, 1H, HC(4)), 1.42 (m, 1H), 1.23 (t, *J* = 7.1 Hz, 3H), 1.10 (ddd, *J* = 12.2, 8.8, 5.7 Hz, 1H); $^{13}C$ NMR (100 MHz, $CDCl_3$) δ ppm 207.0, 139.8, 139.4, 129.2-126.4, 100.4, 87.2, 70.7, 63.5, 59.3, 55.7, 40.3, 34.6, 31.6, 27.6, 26.2, 15.0; HRMS (ESI) calcd. for $C_{32}H_{39}N_2O_4$ $[M + H]^+$ 515.2909 found 515.2912. Compound **12b'**: Yield = 57.7 mg (35%). Pale yellow oil. $[\alpha]_D^{25}$ = +57.8° (*c* 1.60, $CHCl_3$); IR (film) 3061-2804, 1716 (C=O), 1492, 1452, 1357, 1109, 1064, 1039 (N-O), 920 $cm^{-1}$; $^1H$ NMR (400 MHz, $CDCl_3$) δ ppm 7.32-7.13 (m, 15H), 4.56 (dd, *J* = 8.3, 6.5 Hz, 1H, HC(6)), 4.28 (dd, *J* = 9.7, 6.4 Hz, 1H, HC(2)), 3.86 (m, 1H), 3.84 (d, *J* = 12.6 Hz, 2H), 3.55 (m, 1H, HC(3a)), 3.46 (m, 1H), 3.41 (d, *J* = 13.4 Hz, 2H), 3.21 (dd, *J* = 13.8, 4.7 Hz, 1H), 2.98 (ddd, *J* = 9.54, 4.82, Hz, 1H), 2.68 (dd, *J* = 13.7, 9.7 Hz, 1H), 2.23 (s, 3H), 2.20 (m, 1H), 1.84 (ddd, *J* = 13.5, 13,5, 8.3 Hz, 1H), 1.58 (ddd, *J* = 12.0, 9.0, 6.5 Hz, 1H), 1.49 (m, 1H, HC(4)), 1.42 (m, 1H), 1.23 (t, *J* = 7.1 Hz, 3H); $^{13}C$ NMR (100 MHz, $CDCl_3$) δ ppm 209.1, 139.8, 139.4, 129.3-126.2, 100.5, 88.6, 71.2, 63.4, 59.3, 55.5, 40.5, 32.6, 32.1, 27.9, 26.8, 15.0; HRMS (ESI) calcd. for $C_{32}H_{39}N_2O_4$ $[M + H]^+$ 515.2909 found 515.2903

**(1'*S*,2*R*,3a*R*,4*R*,6*S*)-2-acetyl-4-(1'-*N*,*N*-dibenzylamino-3'-methylbutyl)-6-ethoxyperhydroisoxazolidino-[2,3-*b*]-1,2-oxazine (13c) and (1'*S*,2*S*,3a*R*,4*R*,6*S*)-2-acetyl-4-(1'-*N*,*N*-dibenzylamino-3'-methylbutyl)-6-ethoxyperhydroisoxazolidino[2,3-*b*]-1,2-oxazine (13c')**. Nitroalkene **5c** (211.2 mg, 0.624 mmol) was reacted with EVE (1.50 mL, ~25 equiv) and methyl vinyl ketone (1.30 mL, ~25 equiv) for 2 days following the typical procedure 2. Compound **13c**: Yield = 20.5 mg, (7%) as a pale yellow oil. $[\alpha]_D^{25}$ = +35.9° (*c* 1.58, $CHCl_3$); IR (film) 3084–2802, 1720 (C=O), 1494, 1454, 1357, 1109, 1028 (N-O), 920 $cm^{-1}$; $^1H$ NMR (400 MHz, $CDCl_3$) δ ppm 7.31-7.21 (m, 10H), 4.77 (dd, *J* = 10.4, 5.7 Hz, 1H, HC(2)), 4.73 (dd, *J* = 7.9, 6.8 Hz, 1H, HC(6)), 3.94 (dq, *J* = 9.7, 7.1 Hz, 1H), 3.72 (d, *J* = 13.2 Hz, 2H), 3.63 (m, 1H, HC(3a)), 3.50 (dq, *J* =

9.7, 7.1 Hz, 1H), 3.33 (d, *J* = 13.5 Hz, 2H), 2.70 (m, 1H), 2.14 (s, 3H), 2.06 (ddd, *J* = 12.8, 6.7, 2.5 Hz, 1H), 1.85 (m, 1H), 1.78 (m, 1H), 1.61 (m, 1H, HC(4)), 1.51 (m, 3H), 1.27 (m, 1H), 1.23 (t, *J* = 7.2 Hz, 3H), 0.97 (d, *J* = 6.0 Hz, 3H), 0.92 (d, *J* = 6.0 Hz, 3H); $^{13}$C NMR (100 MHz, $CDCl_3$) δ ppm 207.1, 139.8, 129.3-127.2, 100.6, 87.2, 70.8, 63.6, 55.5, 54.6, 41.2, 35.0, 34.3, 27.7, 26.4, 25.6, 24.0, 21.8, 15.0; HRMS (ESI) calcd. for $C_{29}H_{41}N_2O_4$ $[M + H]^+$ 481.3066 found 481.3057. Compound **13c'**: Yield = 80.3 mg (27%). Pale yellow oil. $[\alpha]_D^{25}$ = +44.4° (*c* 1.99, $CHCl_3$); IR (film) 3084–2800, 1720, (C=O), 1494, 1454, 1355, 1111, 1028 (N-O), 916 cm$^{-1}$; $^1$H NMR (400 MHz, $CDCl_3$) δ ppm 7.31-7.21 (m, 10H), 4.65 (t, *J* = 7.7 Hz, 1H, HC(6)), 4.37 (dd, *J* = 9.7, 6.0 Hz, 1H, HC(2)), 3.88 (dq, *J* = 9.6, 7.1 Hz, 1H), 3.70 (d, *J* = 13.2, 2H), 3.61 (q, *J*=8.4 Hz, 1H, HC(3a)), 3.47 (m, 1H), 3.37 (d, *J* = 13.5, 2H), 2.70 (ddd, *J* = 8.9, 4.2, 3.8 Hz, 1H), 2.31 (s, 3H), 2.02 (ddd, *J* = 12.7, 6.4, 2.7 Hz, 1H ), 1.88 (ddd, *J* = 12.0, 8.5, 6.2 Hz, 1 H), 1.79 (m, 1H), 1.61 (m, 1H, HC(4)), 1.52 (m, 3H), 1.40 (m, 1H), 1.23 (t, *J* = 7.1 Hz, 3H), 0.91 (d, *J* = 5.9 Hz, 6H); $^{13}$C NMR (100 MHz, $CDCl_3$) δ ppm 209.2, 139.8, 129.2, 128.2, 127.0, 100.6, 88.6, 71.3, 63.4, 55.3, 54.9, 40.9, 34.8, 32.6, 28.0, 26.8, 25.4, 23.7, 22.0, 15.0

**(1'*S*,2*R*,7*R*,7a*R*)-7-(1'-*N,N*-dibenzylamino-3'-methylbutyl)-2-hydroxypyrrolizin-3-one (14c).** To a solution of **9c** (39.6 mg, 0.079 mmol) in MeOH (9.5 mL) was added a slurry of Raney Ni (previously activated to level W-2 and washed with water and subsequently with MeOH) (151 mg in 3.0 mL of MeOH). This mixture was shaken at room temperature under an atmosphere of $H_2$ (70 PSI) for 48 h, filtered through a short column of Celite eluted with MeOH (15 mL), concentrated in vacuum, and purified by silica gel chromatography eluted with EtOAc to afford **14c** (16.2 mg) in 50% yield as a pale yellow oil. $[\alpha]_D^{25}$ = +27.6° (*c* 0.7, $CHCl_3$); IR (film) 3370 (O-H), 3084–3026, 2924-2803, 2363, 2340, 1686 (C=O), 1454, 1115, 1028, 962 cm$^{-1}$; $^1$H NMR (400 MHz, $CDCl_3$) δ ppm 7.29 (m, 10 H), 4.46 (dd, *J*=10.37, 7.63 Hz, 1 H, HC(2)), 3.70 (d, *J* = 13.69 Hz, 2 H), 3.48 (d, *J* = 13.69 Hz, 2 H), 3.43 (m, 2 H, HC(7a), <u>H</u>CH(5)), 3.15 (dd, *J* = 10.96, 9.78 Hz, 1 H), 2.65 (ddd, *J* = 6.95, 4.89 Hz, 1 H), 2.43 (m, 1 H), 2.26 (ddd, *J* = 11.74, 7.82, 5.87 Hz, 1 H), 1.89 (m, 1 H), 1.80 (m, 1 H, HC(7)), 1.67 (m, 1 H), 1.62 (m, 1 H), 1.52 (ddd, *J* = 11.93, 10.56, 8.80 Hz, 1 H), 1.18 (ddd, *J* = 13.30, 7.24, 5.67 Hz, 1 H), 0.91 (d, *J* = 6.26 Hz, 3 H), 0.86 (d, *J* = 6.26 Hz, 3 H); $^{13}$C NMR (126 MHz, $CDCl_3$) δ ppm 173.9, 139.9, 129.0, 128.2, 127.1, 73.1, 59.3, 55.9, 54.7, 49.8, 40.6, 38.1, 37.9, 31.0, 25.7, 23.2, 22.7; HRMS (ESI) calcd. for $C_{26}H_{35}N_2O_2$ $[M + H]^+$ 407.2699 found 407.2701.

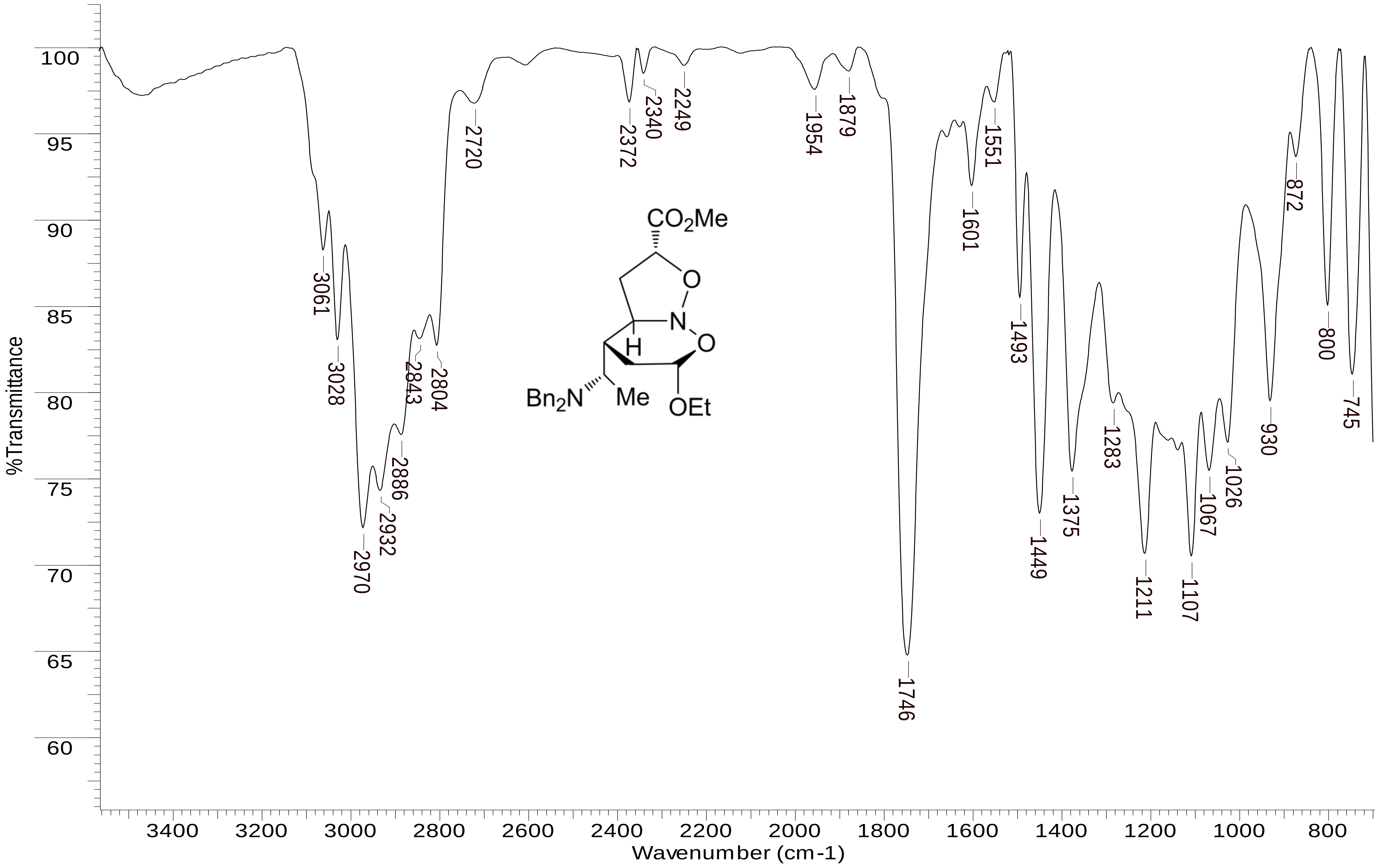


Spectrum 1 – Infrared of **6a**

1H NMR (400 MHz, CHLOROFORM-D) δ ppm 1.05 (d, *J*=6.62 Hz, 3 H); 1.25 (t, *J*=7.06 Hz, 3 H); 1.55 (ddd, *J*=13.67, 13,67, 8.38 Hz, 1 H); 1.75 (m, 1 H); 2.27 (ddd, *J*=12.29, 9.62, 9.62 Hz, 1 H); 2.38 (ddd, *J*=12.18, 7.99, 4.30 Hz, 1 H); 2.55 (ddd, *J*=13.84, 6.67, 3.31 Hz, 1 H); 2.69 (ddd, *J*=13.18, 9.54, 6.62 Hz, 1 H); 3.25 (m, 1 H); 3.32 (d, *J*=13.67 Hz, 2 H); 3.52 (m, 1 H); 3.76 (d, *J*=13.67 Hz, 2 H); 3.74 (s, 3 H); 3.91 (m, 1 H); 4.73 (dd, *J*=7.94, 7.06 Hz, 1 H); 4.99 (dd, *J*=9.92, 4.19 Hz, 1 H); 7.28 (m, 10 $H_{Ar}$)

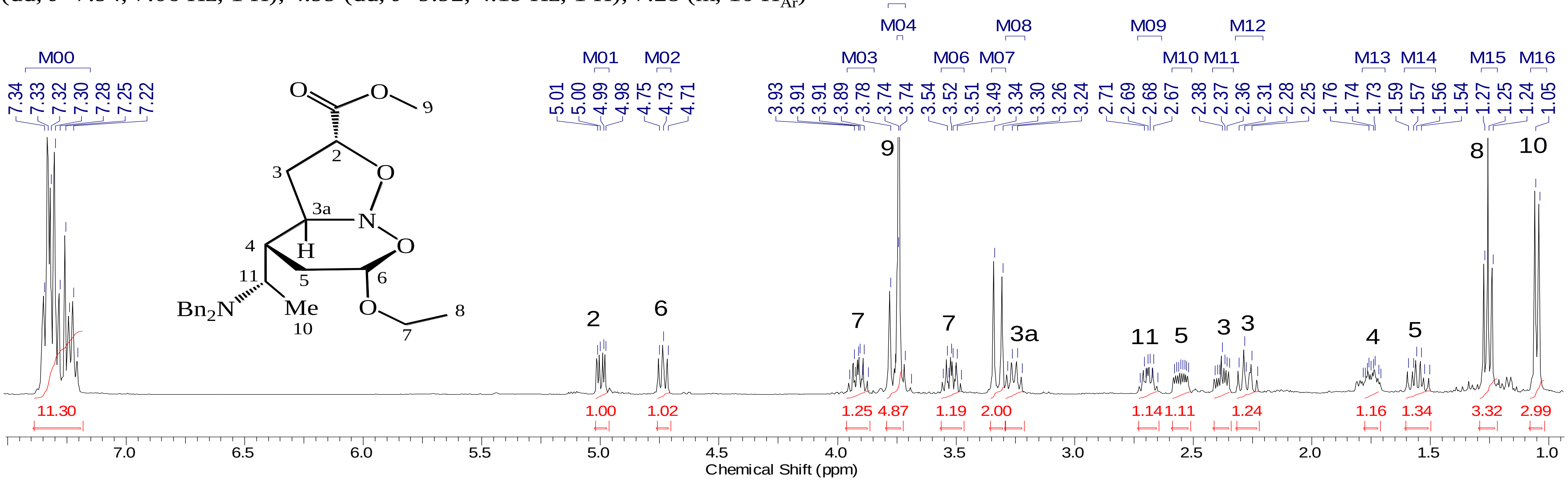


13C NMR (101 MHz, CHLOROFORM-D) δ ppm 9.8 ($CH_3$) 15.1 ($CH_3$) 29.5 ($CH_2$) 36.7 ($CH_2$) 42.5 (CH) 52.4 ($CH_3$) 53.8 (2x $CH_2$) 55.5 (CH) 63.1 ($CH_2$) 72.0 (CH) 80.8 (CH) 100.5 (CH) 127.0-128.7 (10x $CH_{Ar}$) 139.4 (2x $C_{ipso}$) 170.6 (C=O)

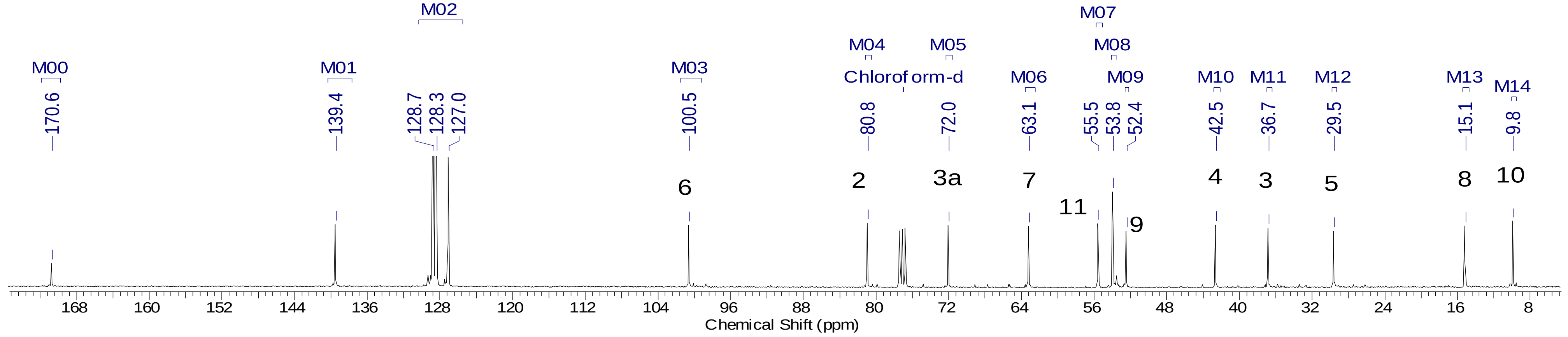


Spectrum 2 – $^1$H and $^{13}$C NMR of **6a**

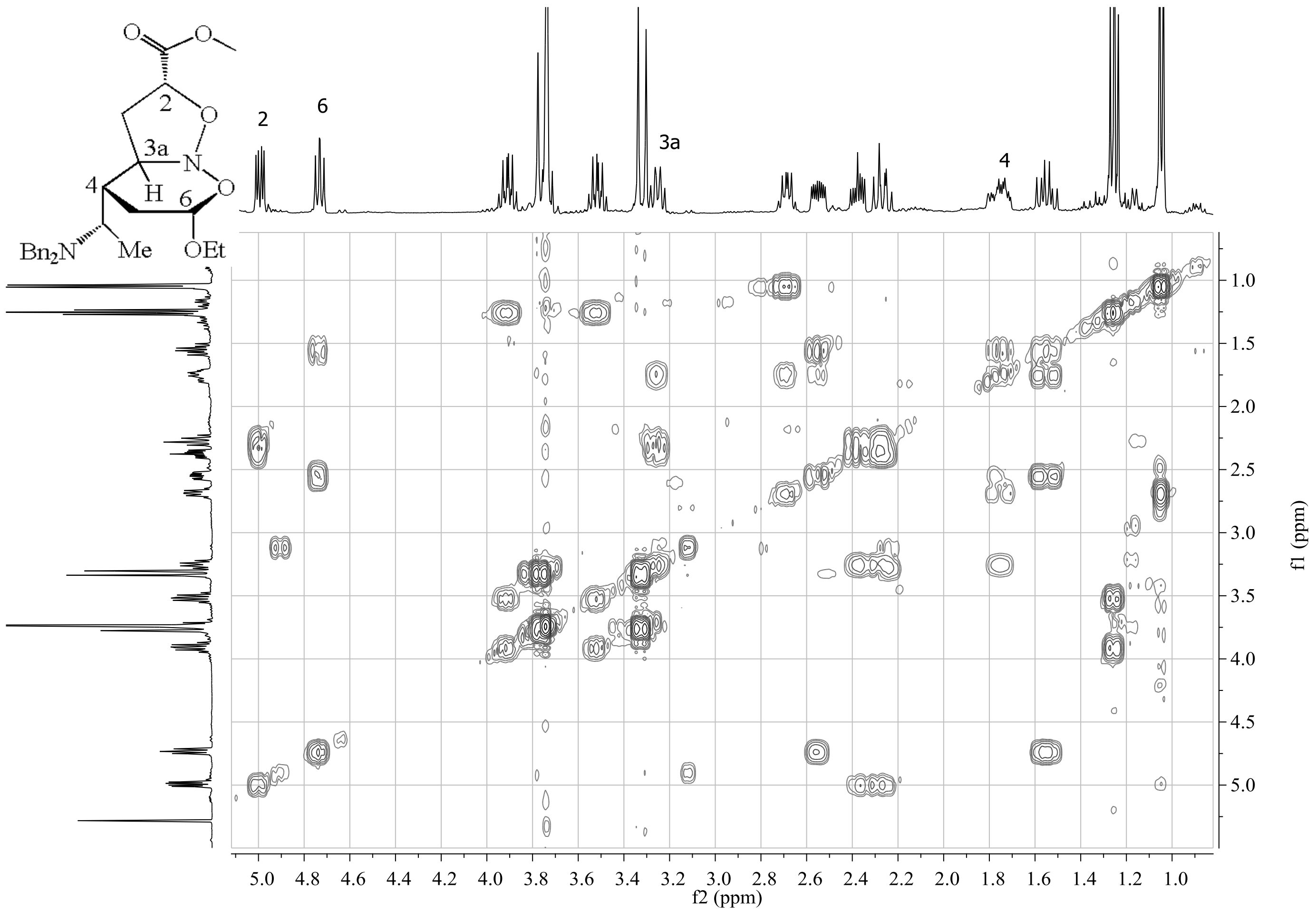


Spectrum 3 – 2D COSY of **6a**

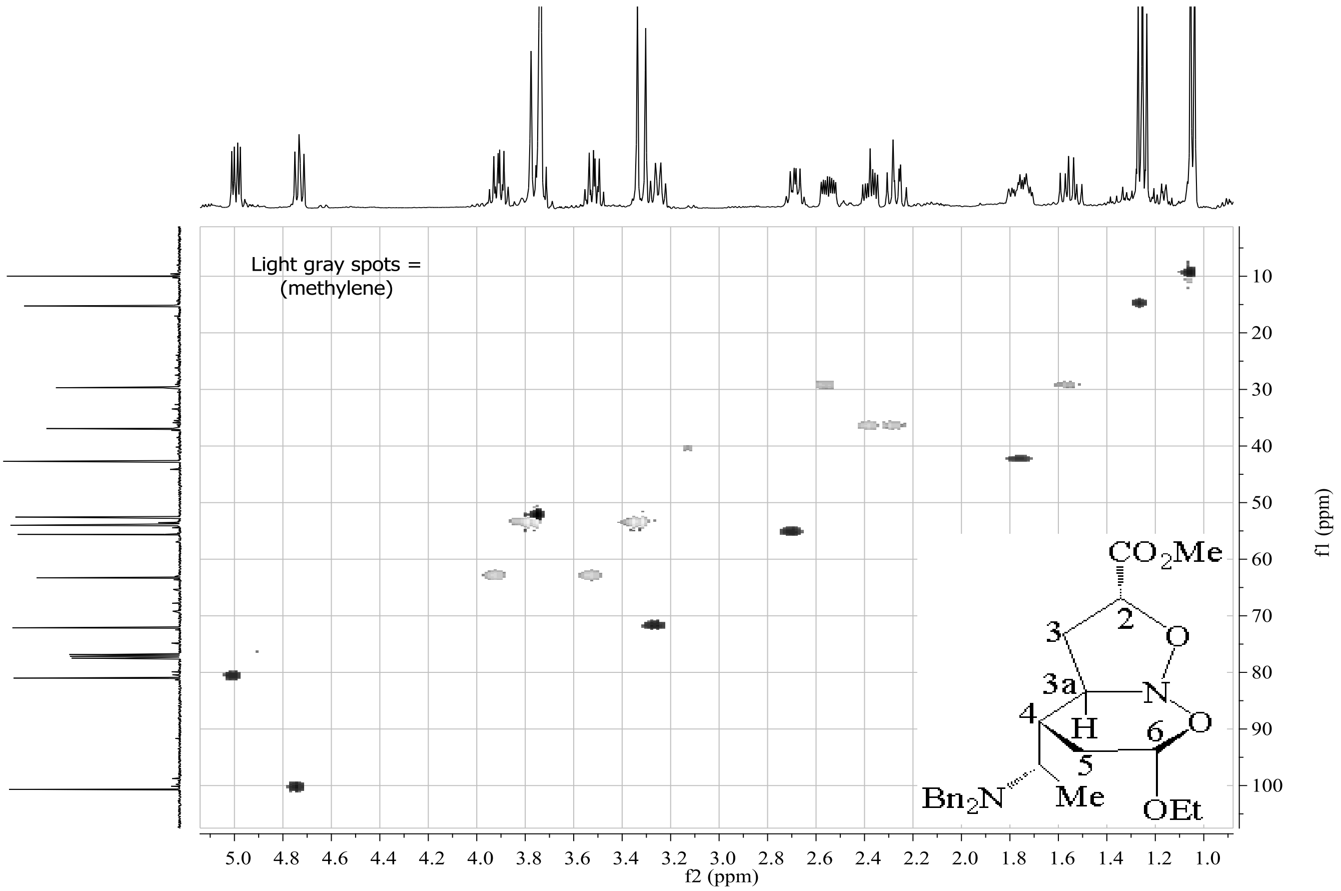


Spectrum 4 – HSQC of **6a**

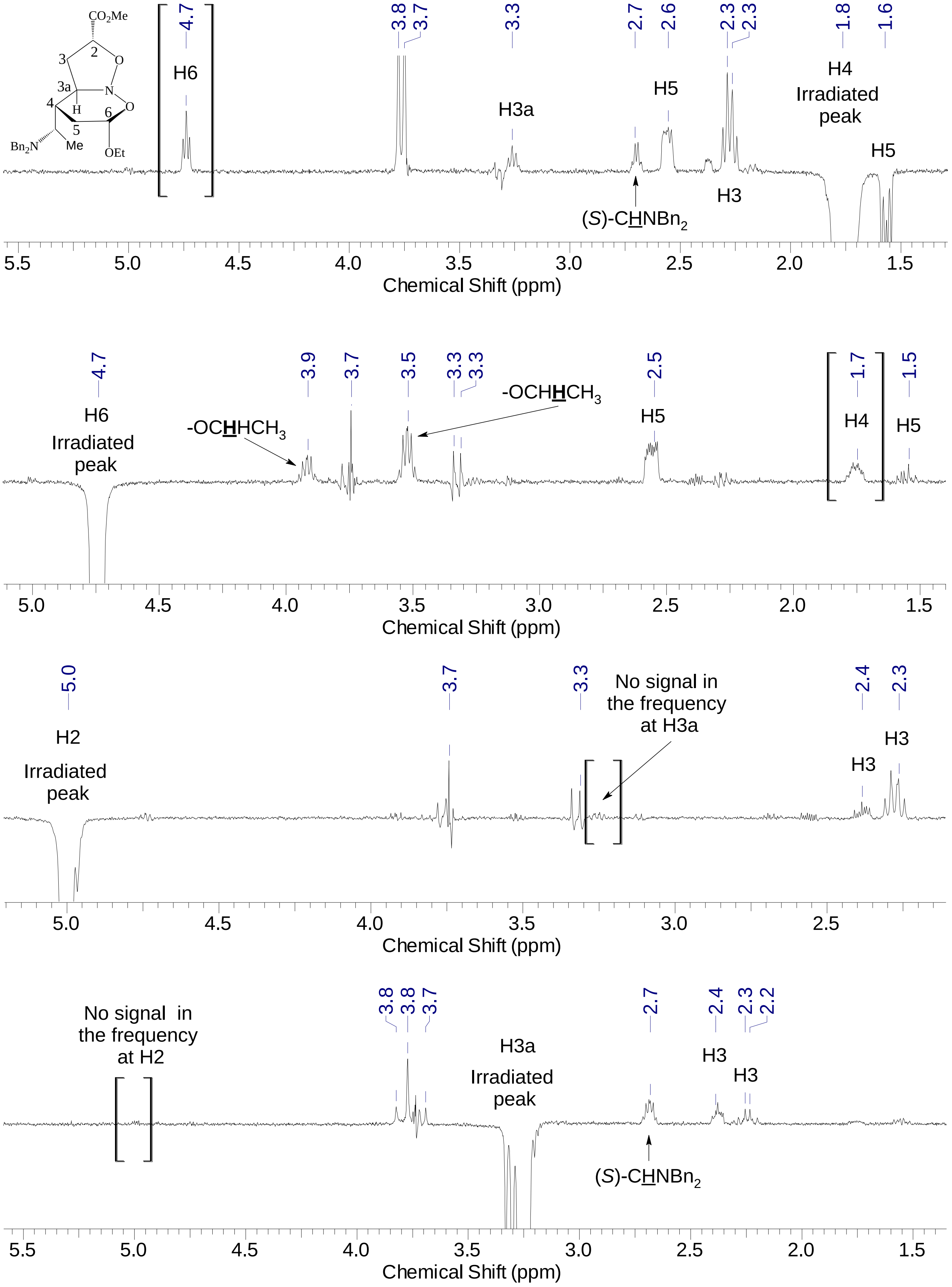


Spectrum 5 – 2D NOESY of **6a**

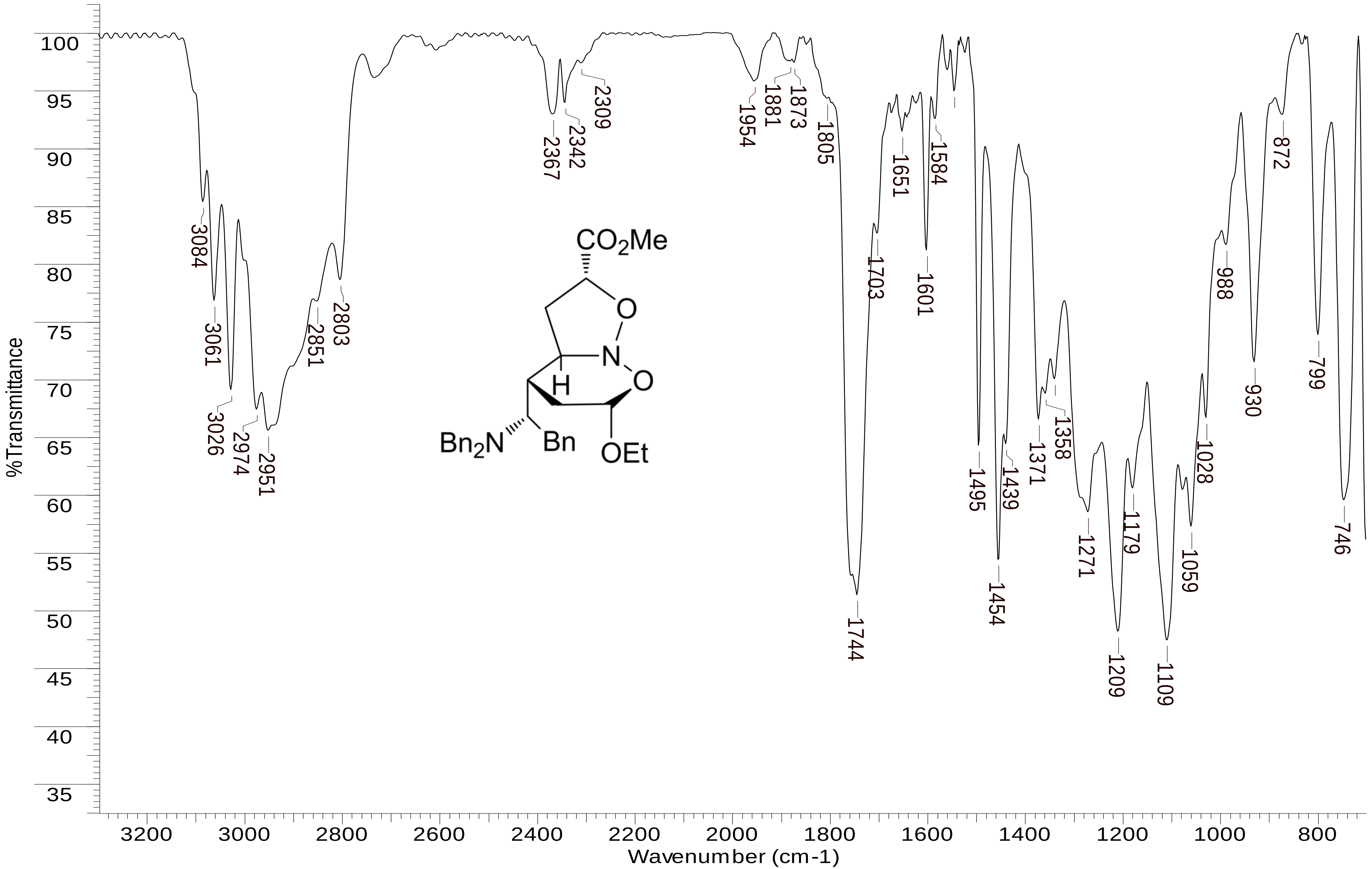


Spectrum 6 – Infrared of **7b**

1H NMR (400 MHz, CHLOROFORM-D) δ ppm 1.21 (t, *J*=7.03 Hz, 3 H); 1.32 (m, 1 H); 1.47 (m, 1 H); 1.54 (m, 1 H); 1.88 (ddd, *J*=13.51, 13.51, 8.44 Hz, 1 H); 2.22 (ddd, *J*=12.81, 6.45, 2.38 Hz, 1 H); 2.68 (dd, *J*=13.33, 10.39 Hz, 1 H); 2.93 (m, 1 H); 3.21 (dd, *J*=13.51, 4.22 Hz, 1 H); 3.41 (d, *J*=13.45 Hz, 2 H); 3.46 (m, 1 H); 3.76 (m, 1 H); 3.73 (s, 3 H); 3.86 (d, *J*=14.06 Hz, 2 H); 3.89 (m, 1 H); 4.62 (dd, *J* = 7.95, 6.85 Hz, 1 H); 4.78 (dd, *J*=10.15, 5.01 Hz, 1 H); 7.24 (m, 15 H)

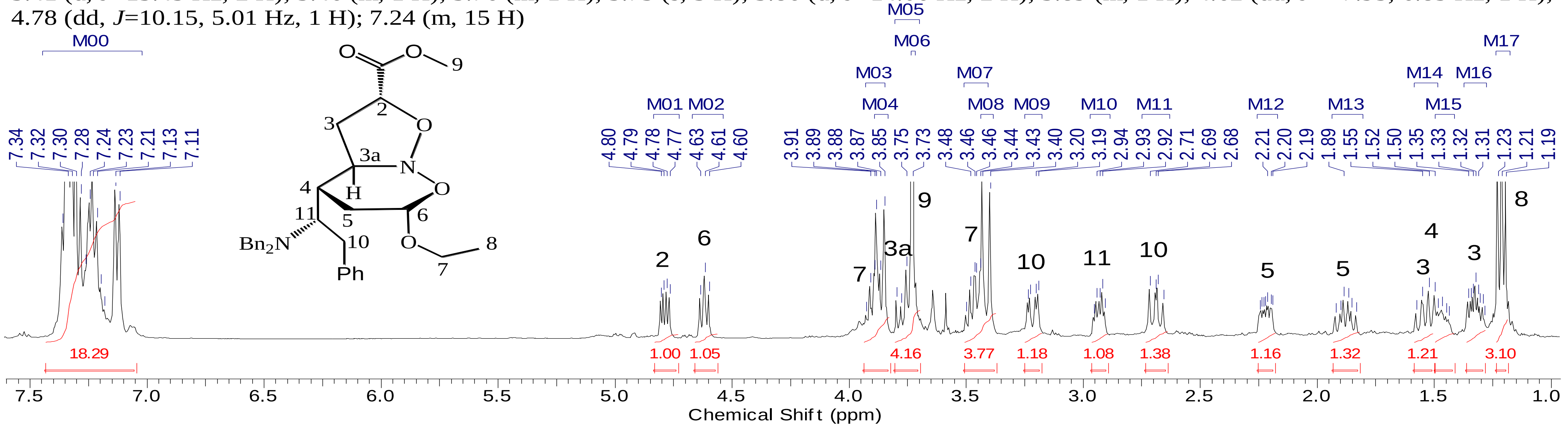


13C NMR (101 MHz, CHLOROFORM-D) δ ppm 15.0 ($CH_3$); 27.7 ($CH_2$); 31.5 ($CH_2$); 35.1 ($CH_2$); 40.3 (CH); 52.1 ($CH_3$); 55.5 (2x $CH_2$); 59.2 (CH); 63.2 (CH2); 70.5 (CH); 80.5 (CH); 100.3 (CH); 126.2-129.1 (15x $CH_{Ar}$); 139.3 (2x $C_{ipso}$); 139.8 ($C_{ipso}$); 170.3 (C=O)

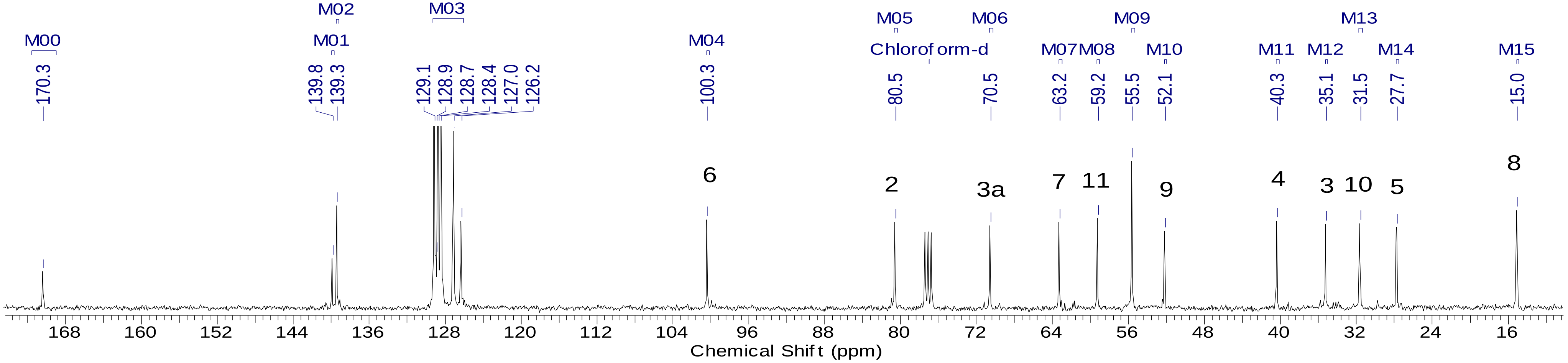


Spectrum 7 - $^{1}$H and $^{13}$C NMR of **7b**

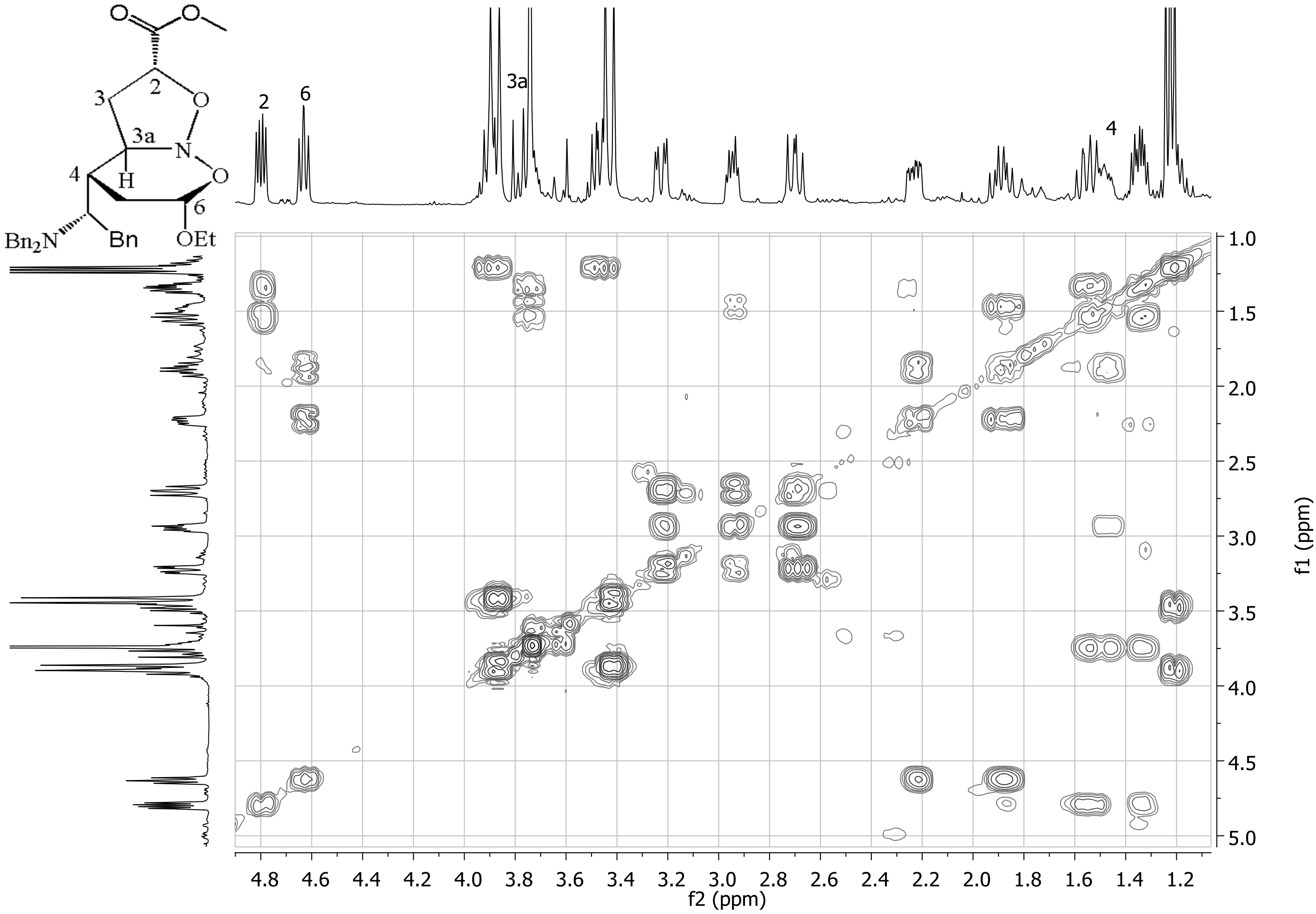


Spectrum 8 – 2D COSY of **7b**

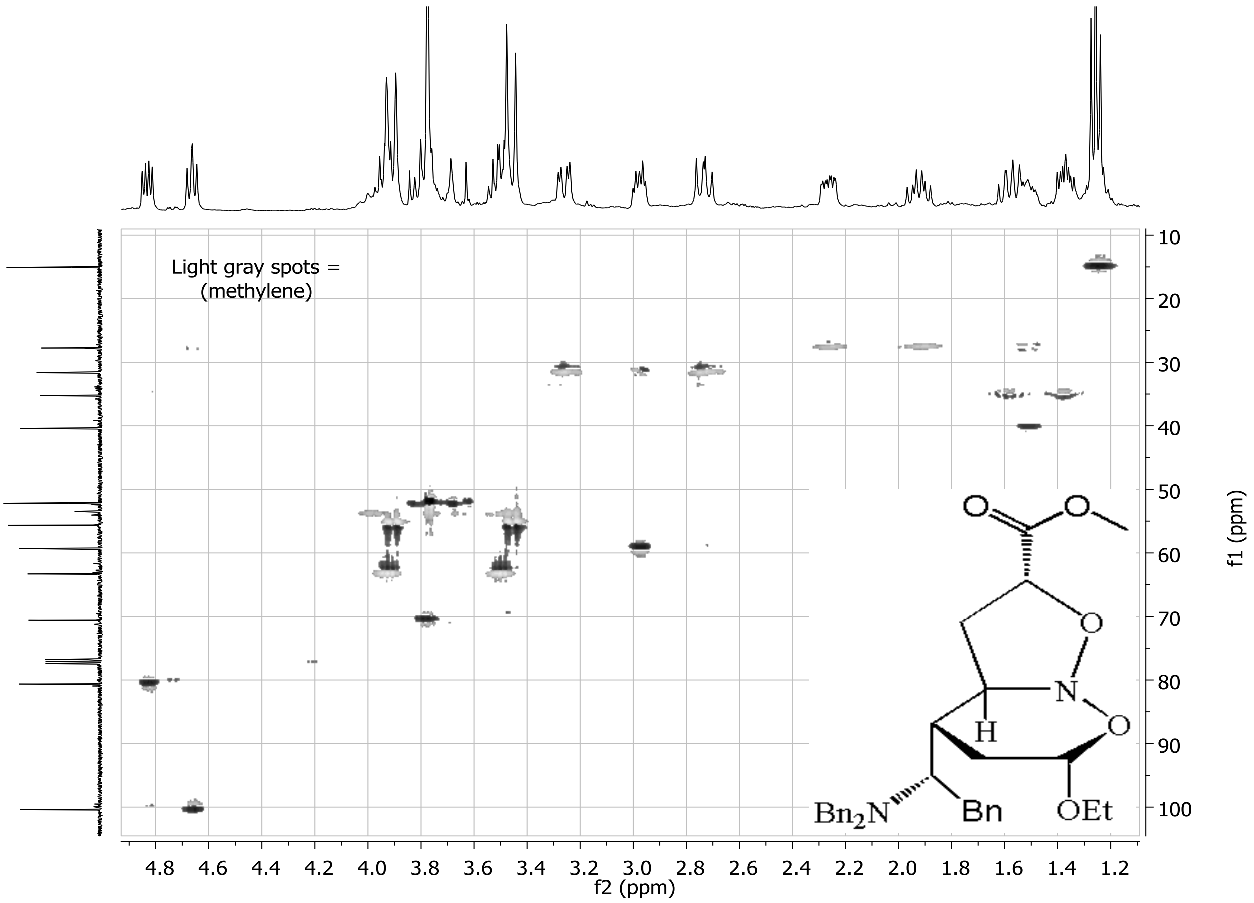


Spectrum 9 – HSQC of **7b**

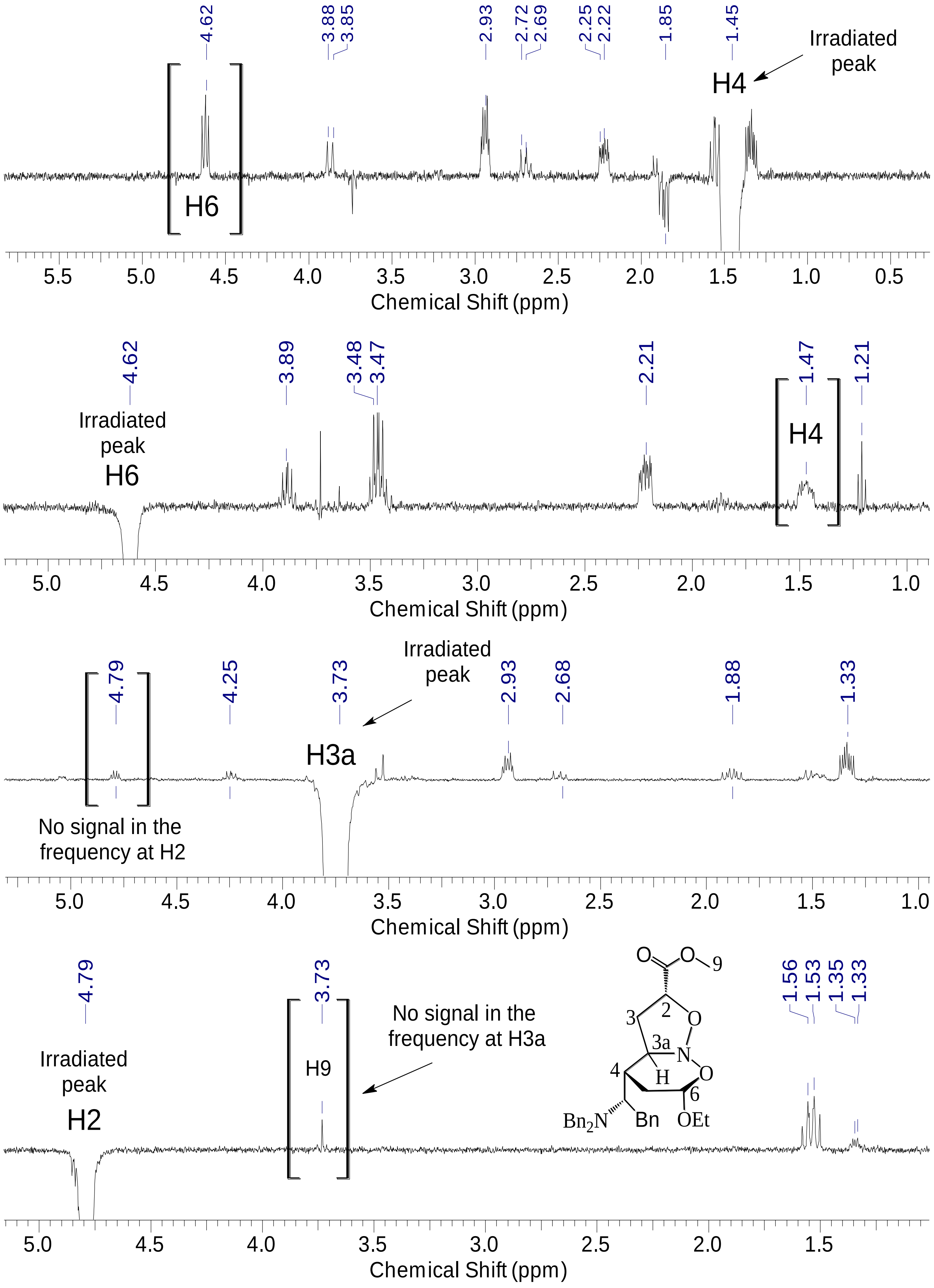


Spectrum 10 – 2D NOESY of **7b**

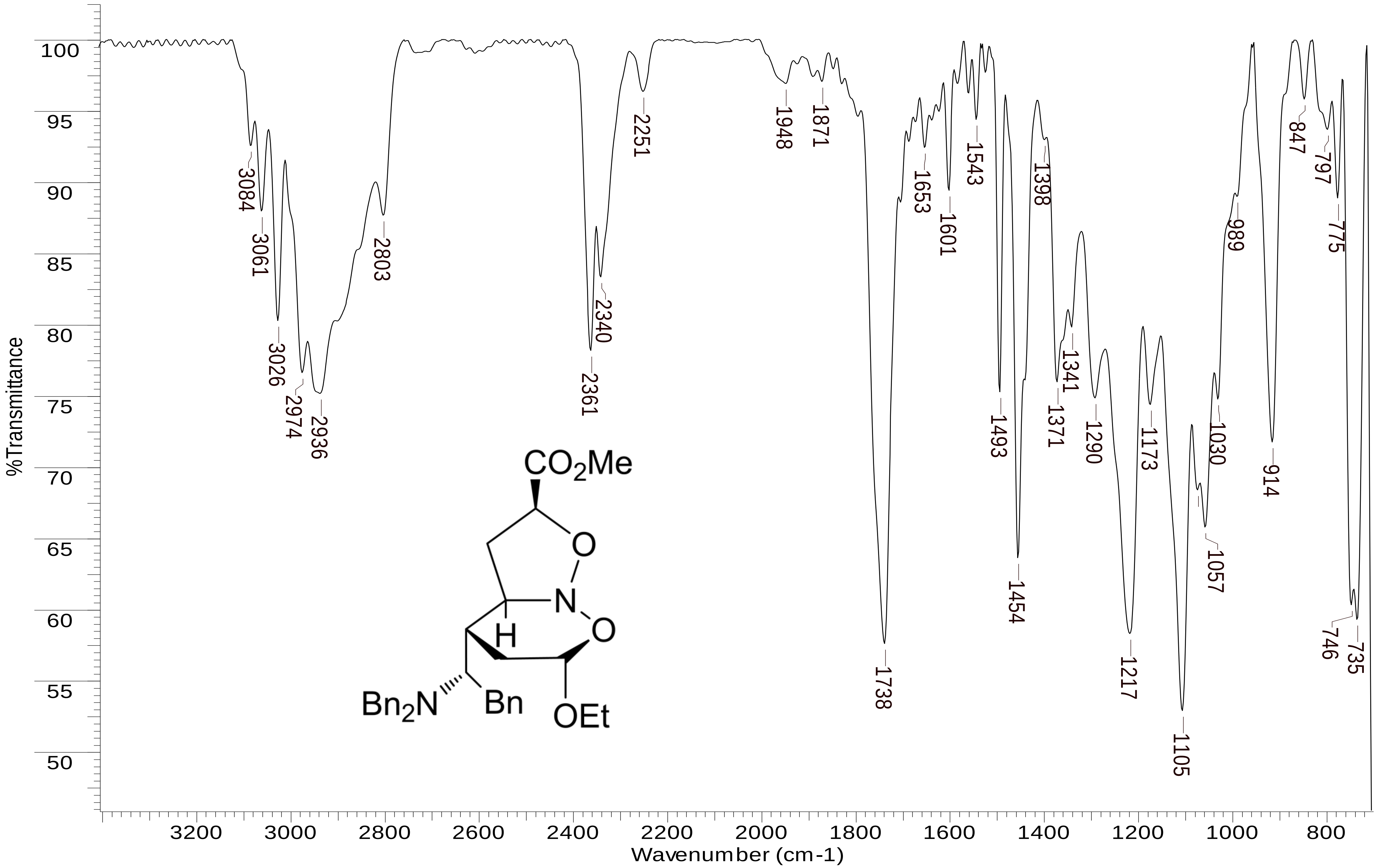


Spectrum 11 – Infrared of **7b’**

1H NMR (400 MHz, CHLOROFORM-D) δ ppm 1.21 (t, *J*=6.98 Hz, 3 H); 1.42 (m, 1 H); 1.56 (m, 1 H); 1.69 (m, 1 H); 1.86 (m, 1 H); 2.21 (m, 1 H); 2.70 (dd, *J*=13.38, 10.12 Hz, 1 H); 2.97 (m, 1 H); 3.22 (dd, *J*=13.61, 4.07 Hz, 1 H); 3.42 (d, *J*=13.72 Hz, 2 H); 3.47 (m, 1 H); 3.57 (m, 1 H); 3.67 (s, 3 H); 3.85 (d, *J*=13.26 Hz, 2 H); 3.93 (m, 1 H); 4.38 (t, *J*=8.37 Hz, 1 H); 4.63 (dd, *J*=7.44, 7.21 Hz, 1 H); 7.24 (m, 15 H)

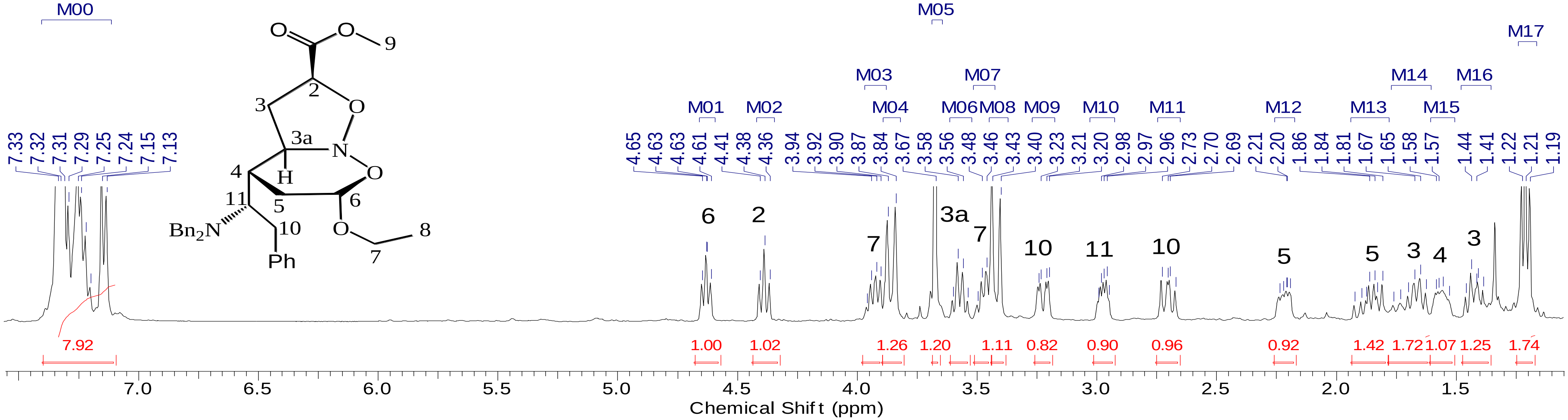


13C NMR (101 MHz, CHLOROFORM-D) δ ppm 15.0 ($CH_3$); 27.9 ($CH_2$); 31.9 ($CH_2$); 34.0 ($CH_2$); 40.1 (CH); 52.3 ($CH_3$); 55.5 (2x $CH_2$);59.3 (CH); 63.3 ($CH_2$); 71.1 (CH); 81.5 (CH); 100.5 (CH); 126.3-129.2 (15x $CH_{Ar}$); 139.5 (2x $C_{ipso}$); 139.9 ($C_{ipso}$); 171.2 (C=O)

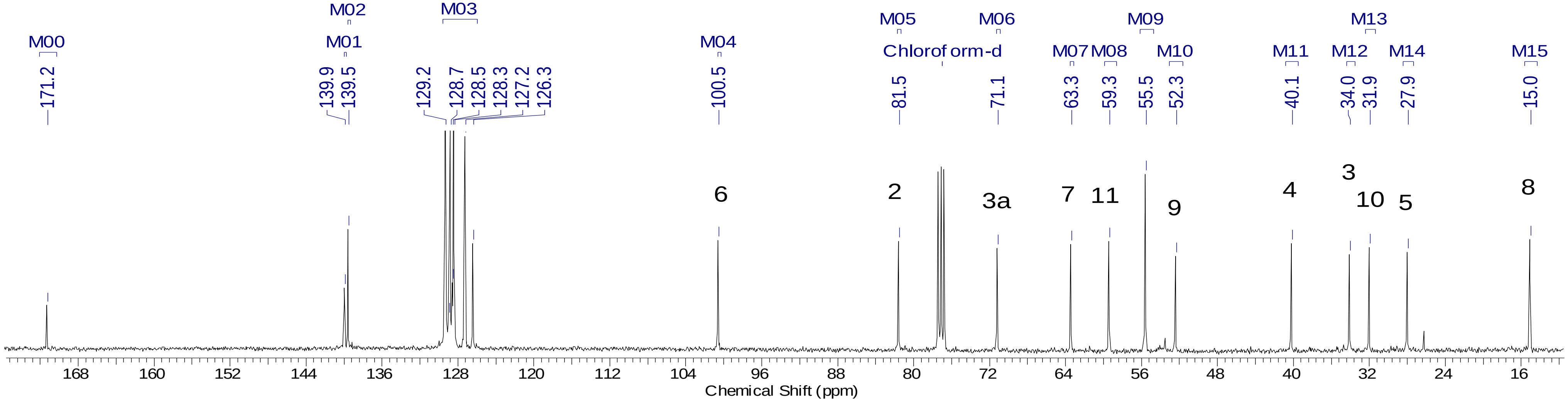


Spectrum 12 - $^{1}$H and $^{13}$C NMR of **7b'**

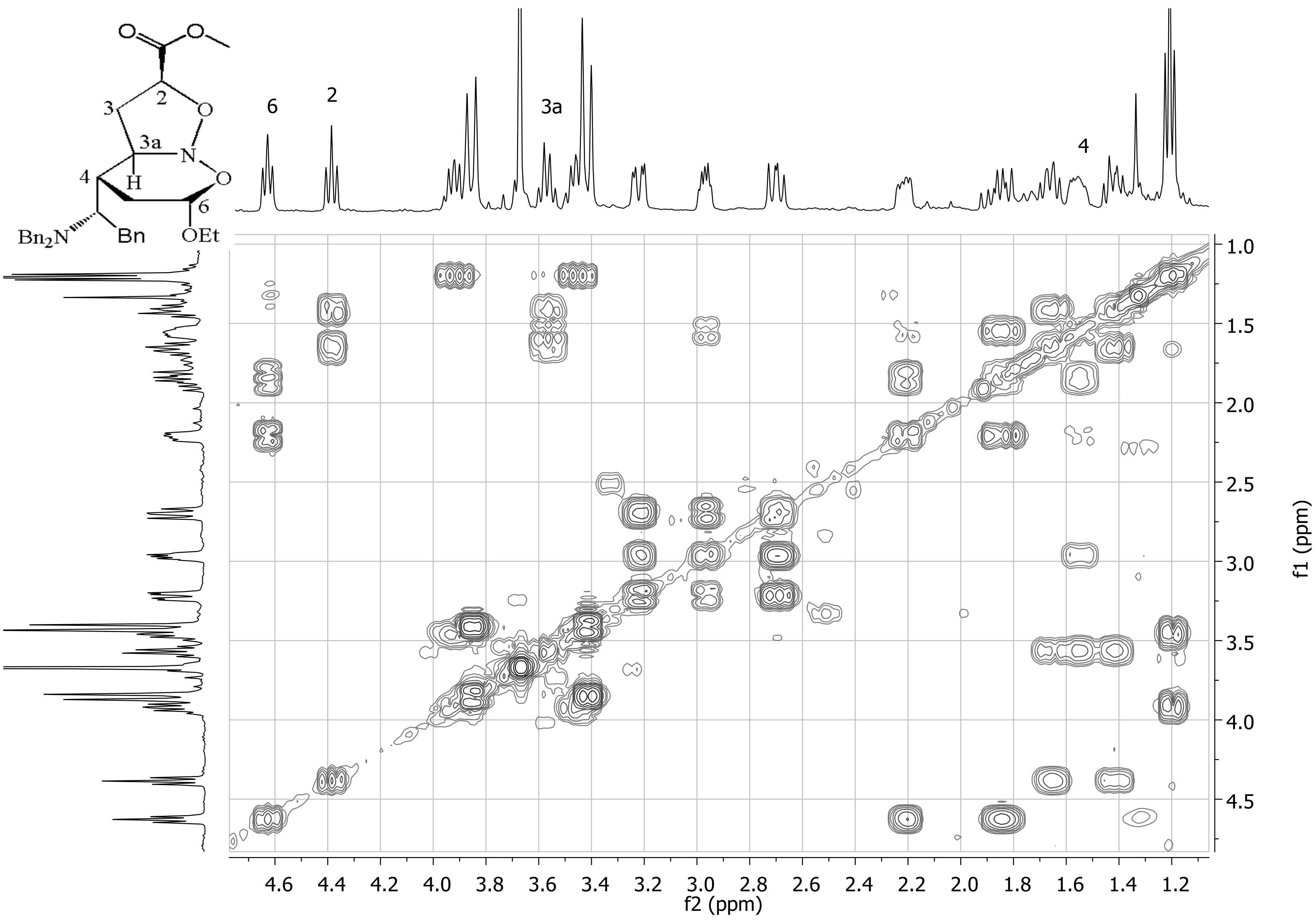


Spectrum 13 – 2D COSY of **7b’**

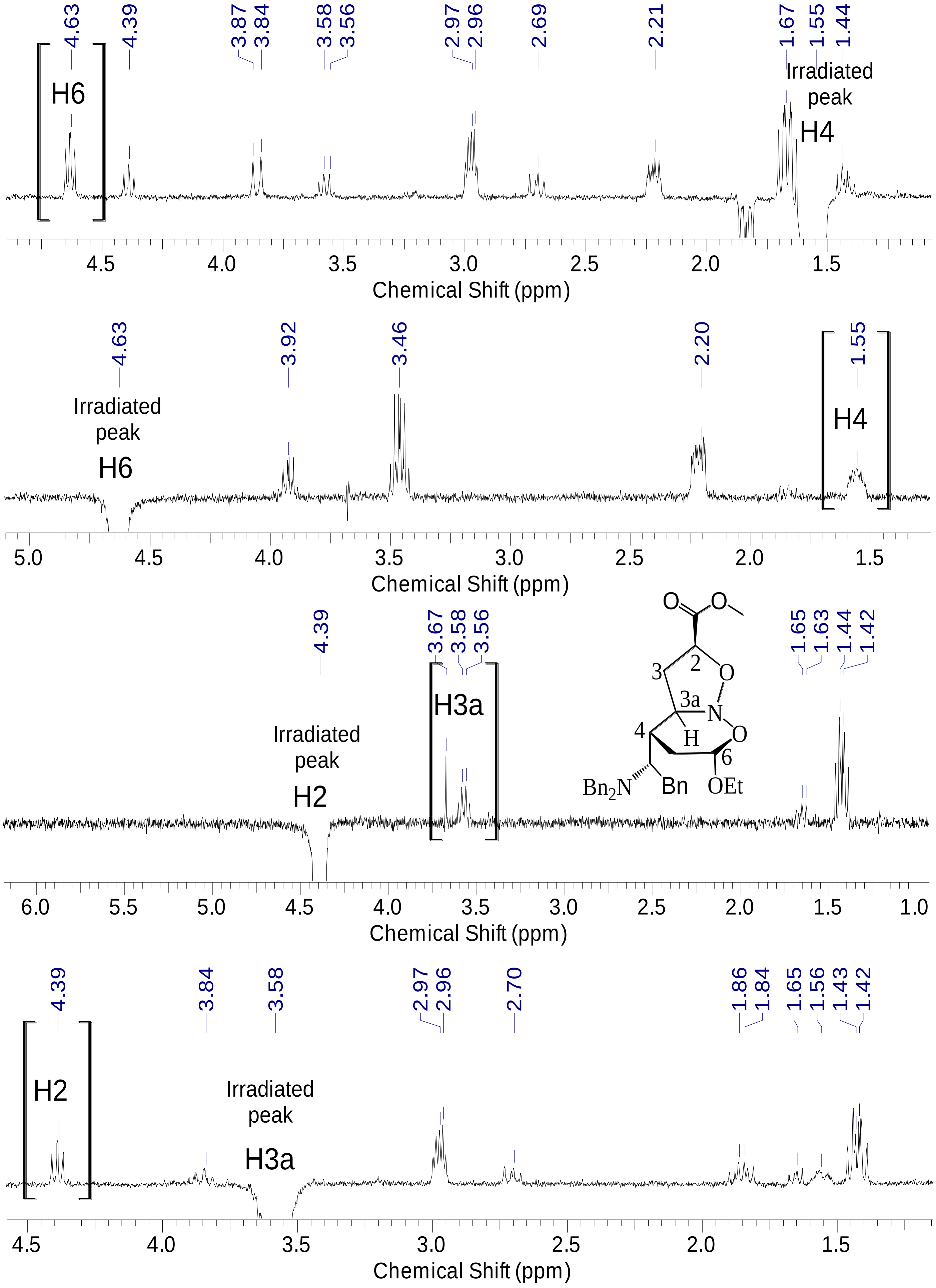


Spectrum 14 – 2D NOESY of **7b'**

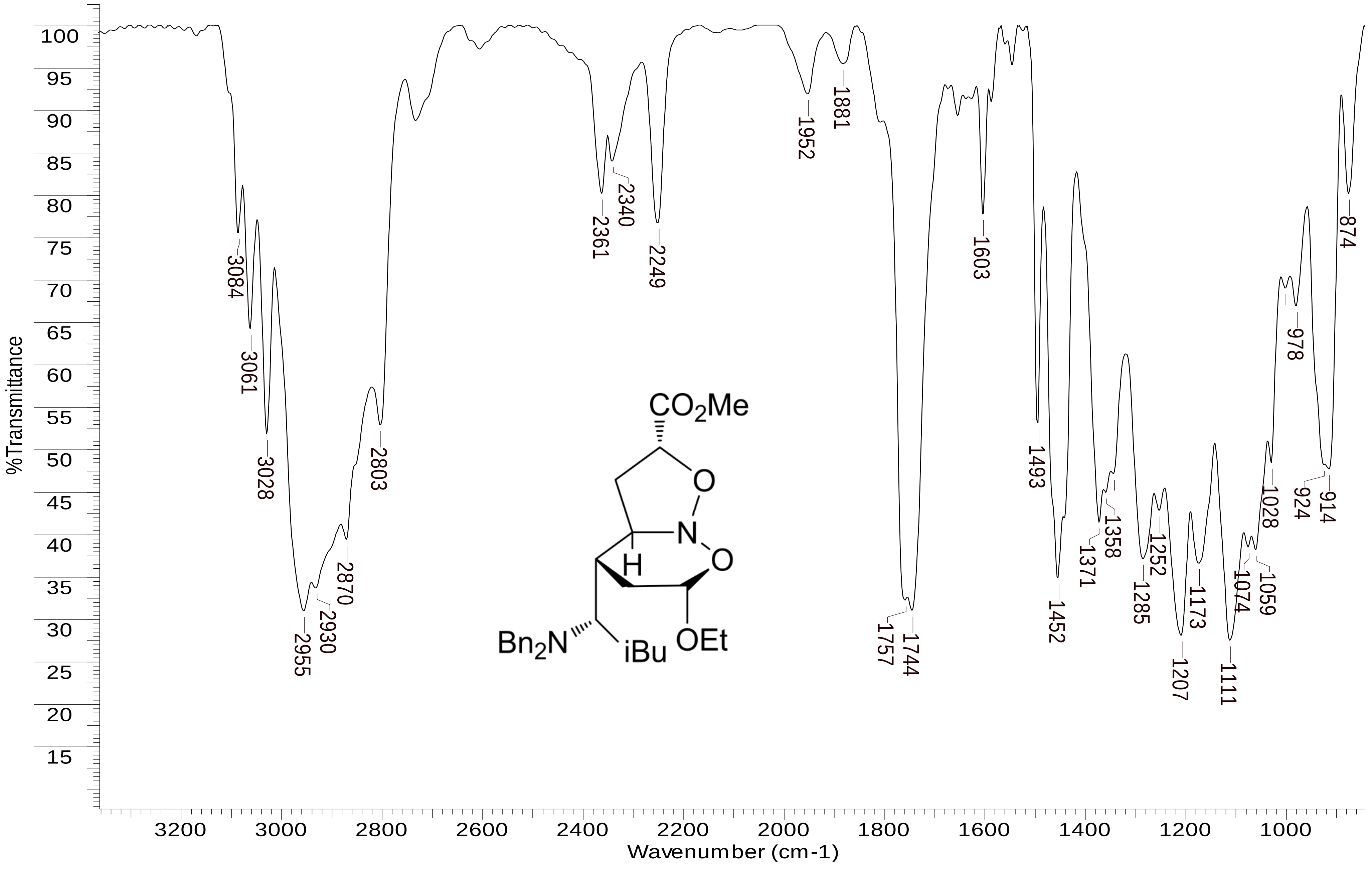


Spectrum 15 – Infrared of **9c**

1H NMR (400 MHz, CHLOROFORM-D) δ ppm 0.89 (d, *J*=6.11 Hz, 3 H); 0.93 (d, *J*=6.11 Hz, 3 H); 1.21 (t, *J*=7.08 Hz, 3 H); 1.42 (m, 1 H); 1.54 (m, 3 H); 1.64 (m, 1 H); 1.87 (m, 2 H); 2.06 (ddd, *J*=12.93, 6.59, 2.83 Hz, 1 H); 2.70 (m, 1 H); 3.36 (d, *J*=13.41 Hz, 2 H); 3.48 (dq, *J* = 9.6, 7.0, 1H); 3.76 (m, 1 H); 3.72 (d, *J*=13.41 Hz, 2 H); 3.78 (s, 3 H); 3.92 (dq, *J* = 9.6, 7.1, 1 H); 4.72 (dd, *J*=8.05, 6.71 Hz, 1 H); 4.93 (dd, *J*=9.98, 4.92 Hz, 1 H); 7.25 (m, 10 H)

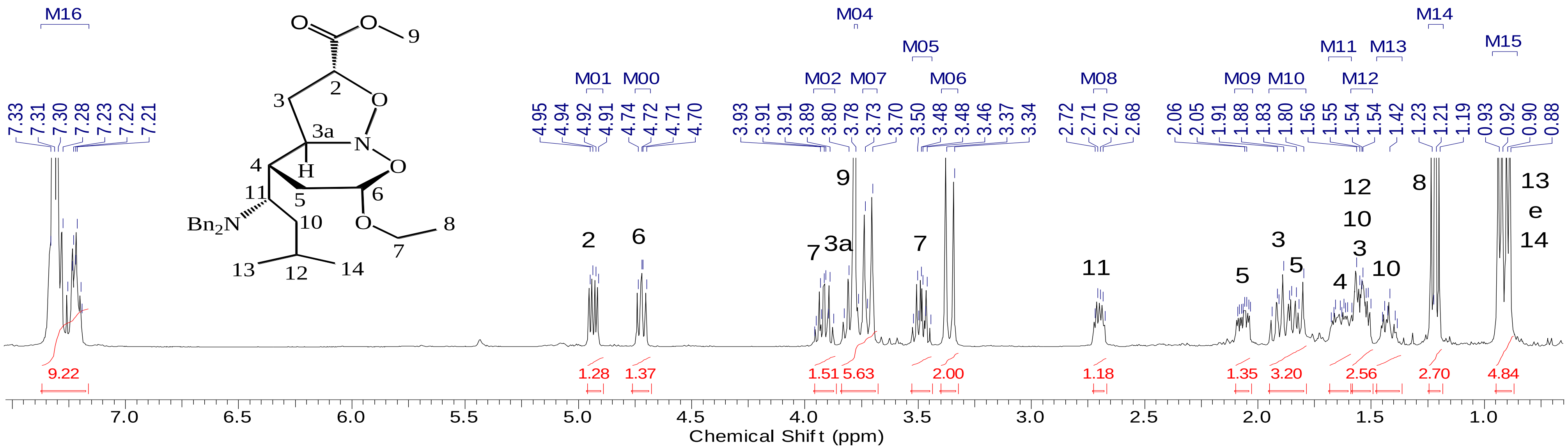


13C NMR (101 MHz, CHLOROFORM-D) δ ppm 15.0 ($CH_3$); 21.9 ($CH_3$); 23.7 ($CH_3$); 25.4 (CH); 27.9 ($CH_2$); 34.5 ($CH_2$); 35.5 ($CH_2$); 41.0 (CH); 52.2 ($CH_3$); 54.8 (CH); 55.3 (2x $CH_2$); 63.2 ($CH_2$); 70.6 (CH); 80.6 (CH); 100.5 (CH); 126.9; 128.3; 129.1 (10x $CH_{Ar}$); 139.7 (2x $C_{ipso}$); 170.4 (C=O)

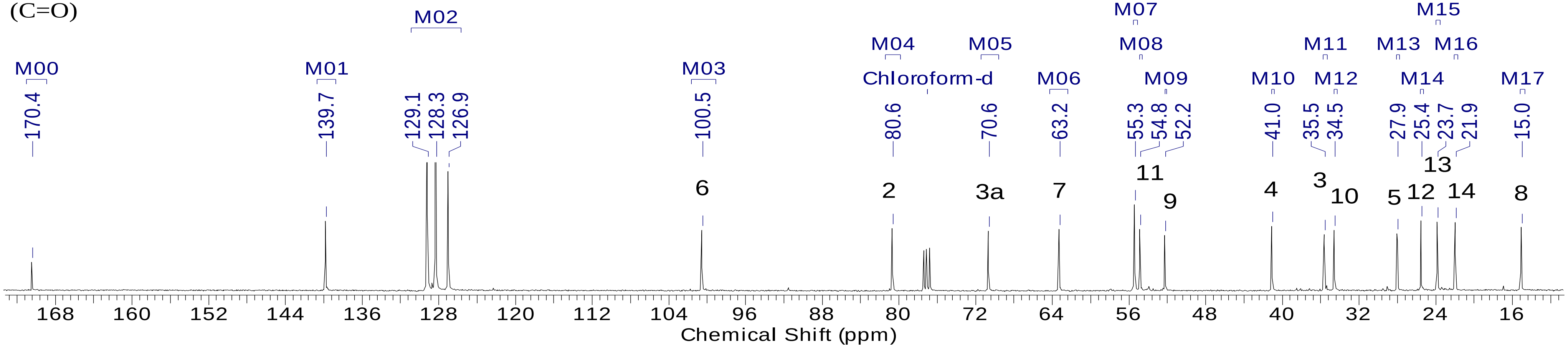


Spectrum 16 - $^{1}$H and $^{13}$C NMR of **9c**

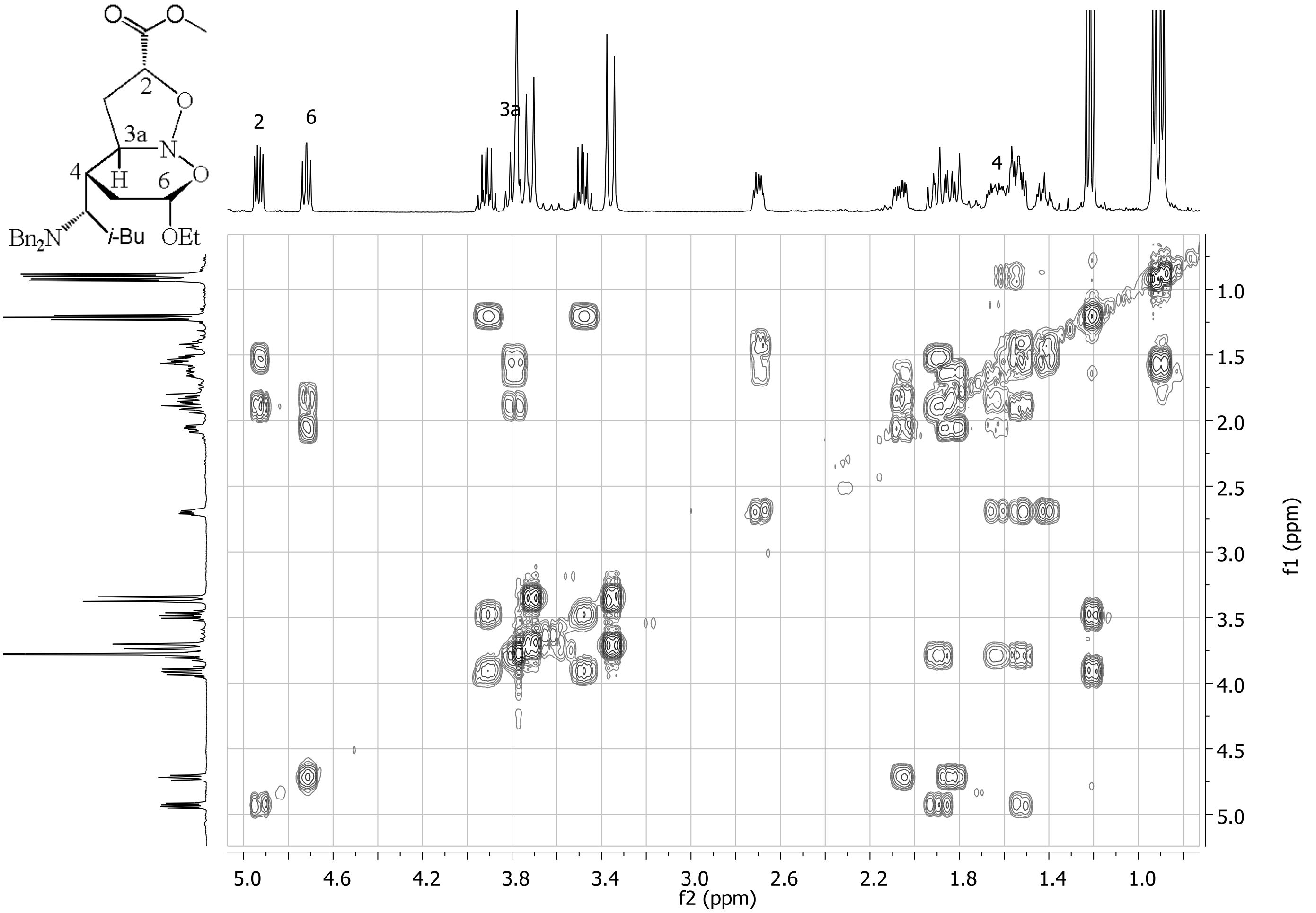


Spectrum 17 – 2D COSY of **9c**

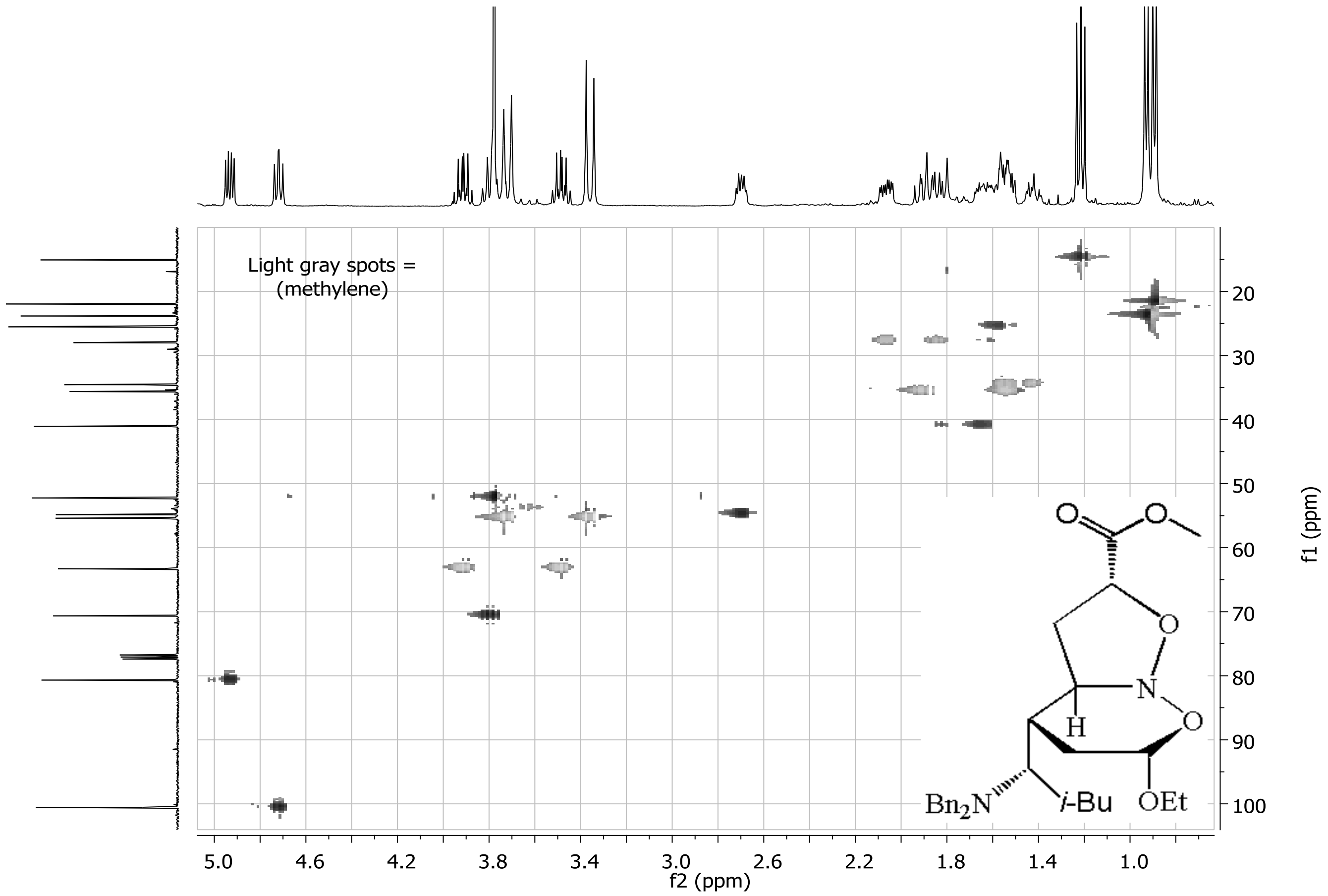


Spectrum 18 – HSQC of **9c**

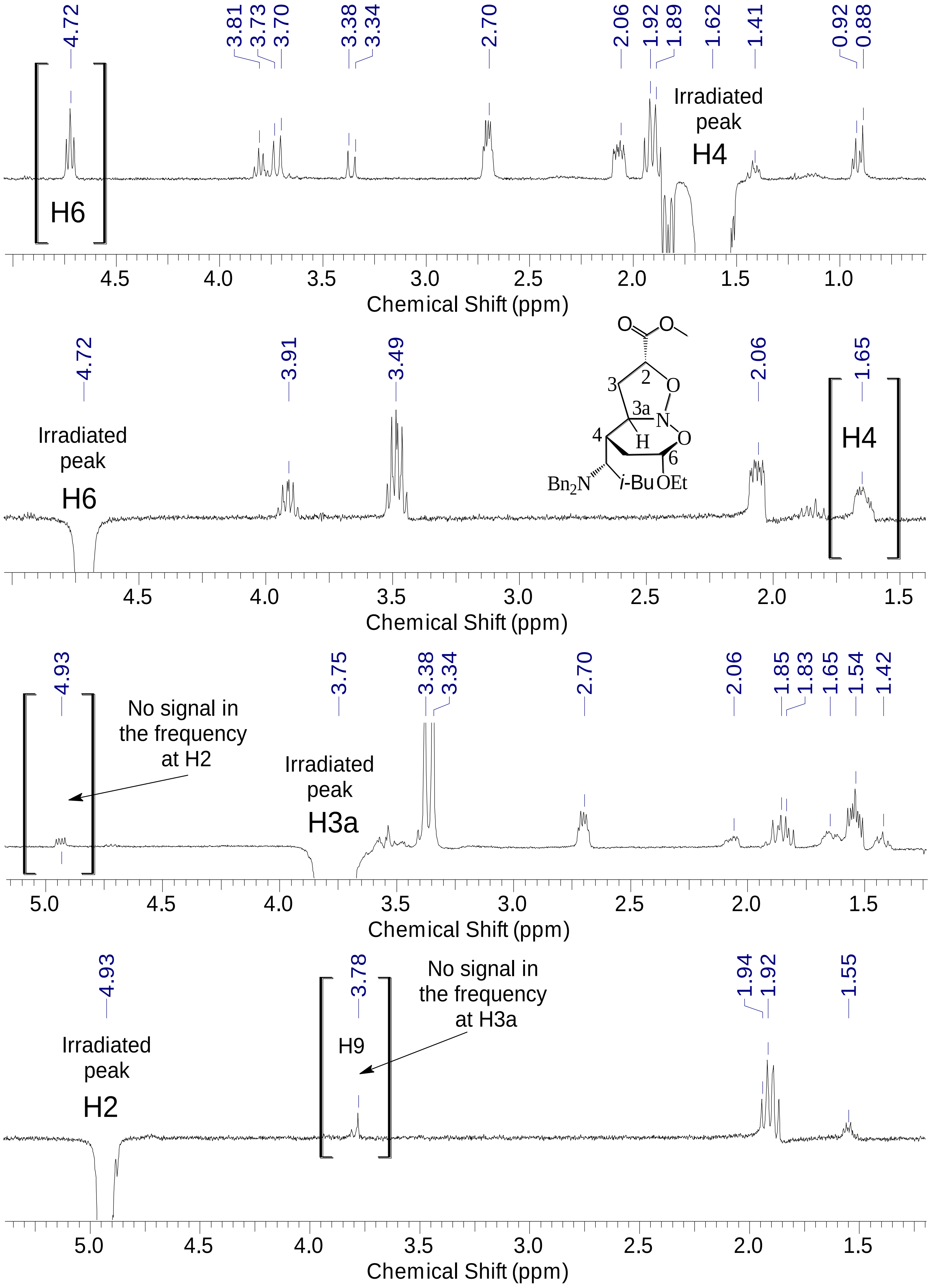


Spectrum 19 – 2D NOESY of **9c**

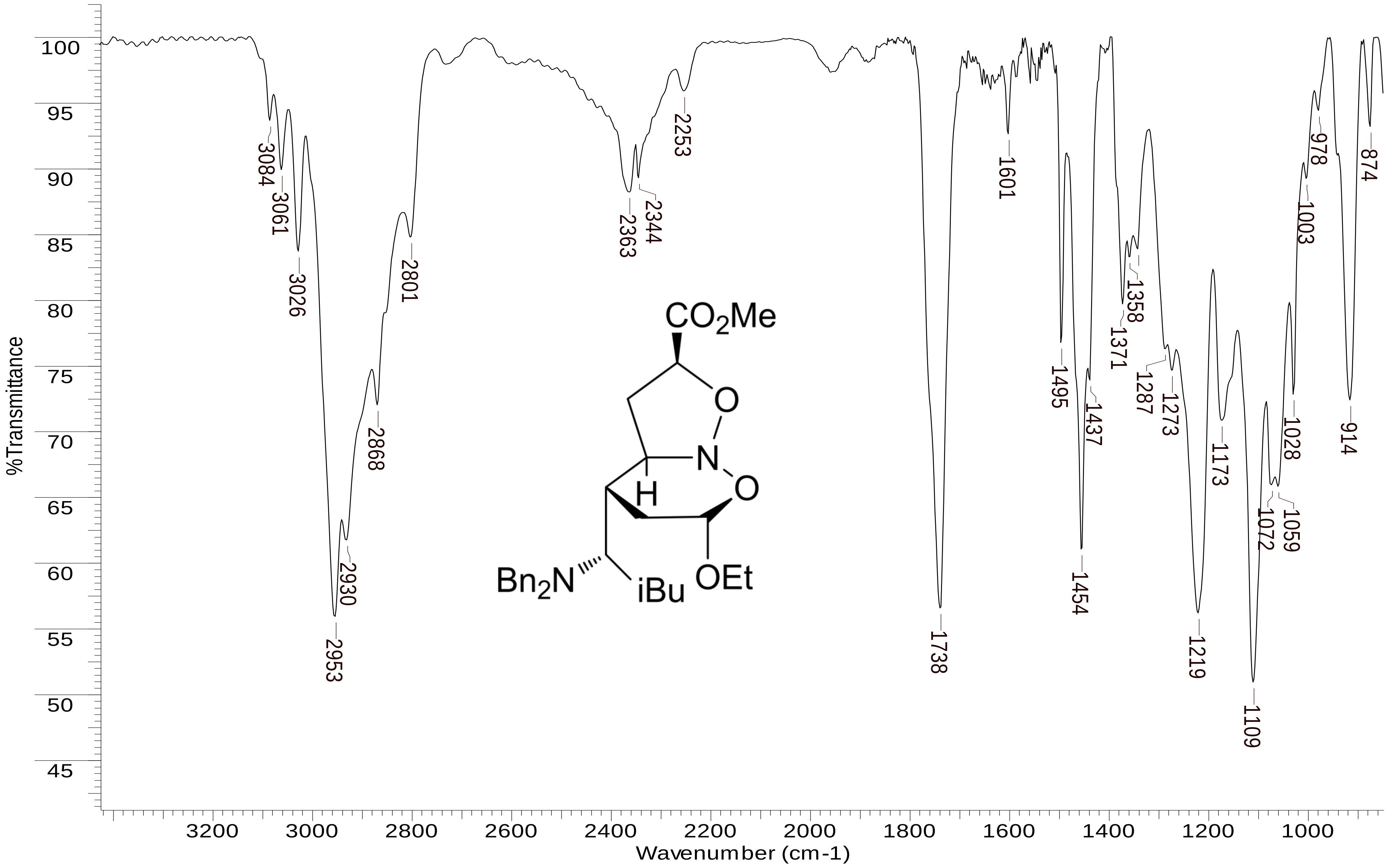


Spectrum 20 – Infrared of **9c’**

1H NMR (400 MHz, CHLOROFORM-D) δ ppm 0.91 (d, *J* = 5.29 Hz, 3 H); 0.92 (d, *J* = 5.07 Hz, 3 H); 1.21 (t, *J*=7.06 Hz, 3 H); 1.56 (m, 3 H); 1.69 (m, 1 H); 1.81 (m, 1 H); 1.98 (m, 1 H); 2.04 (ddd, *J*=12.68, 6.51, 2.43 Hz, 1 H); 2.71 (m, 1 H); 3.37 (d, *J*=13.45 Hz, 2 H); 3.47 (dq, *J* = 9.67, 7.07 Hz, 1 H); 3.63 (m, 1 H); 3.73 (m, 1 H); 3.71 (d, *J*=13.45 Hz, 2 H); 3.75 (s, 3 H); 3.93 (m, 1 H); 4.49 (dd, *J*=9.04, 7.06 Hz, 1 H); 4.70 (d, *J*=7.94, 6.62 Hz, 1 H); 7.27 (m, 10 H)

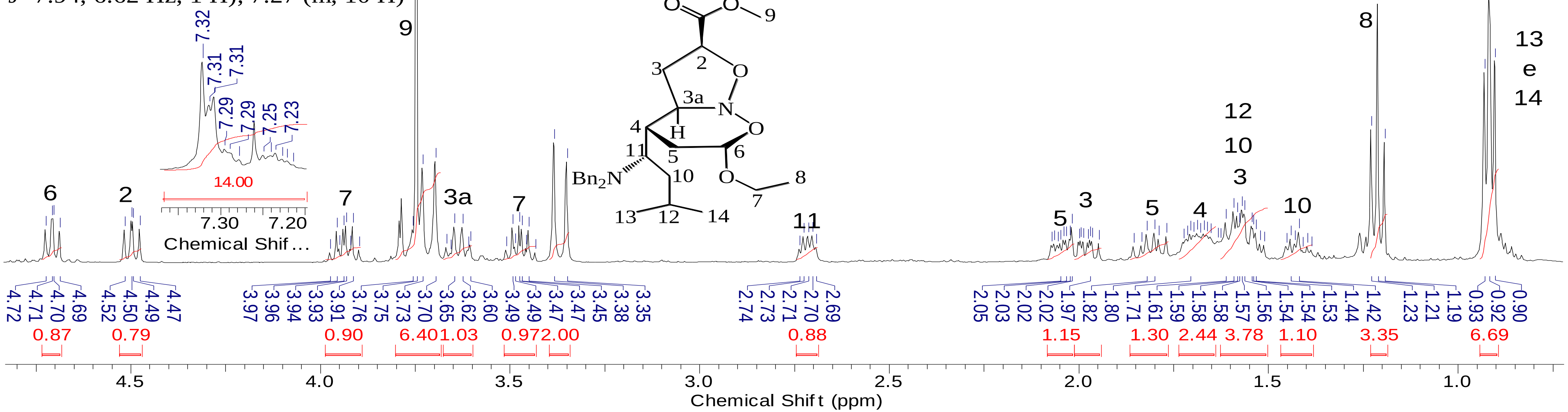


13C NMR (101 MHz, CHLOROFORM-D) δ ppm 15.0 ($CH_3$); 22.0 ($CH_3$); 23.8 ($CH_3$); 25.5 (CH); 28.1 ($CH_2$); 34.1 ($CH_2$); 34.8 ($CH_2$); 40.7 (CH); 52.4 ($CH_3$); 54.9 (CH); 55.4 (2x $CH_2$); 63.4 ($CH_2$); 71.3 (CH); 81.5 (CH); 100.6 (CH); 127.1-129.3 (10x $CH_{Ar}$); 139.9 (2x $C_{ipso}$); 171.5 (C=O)

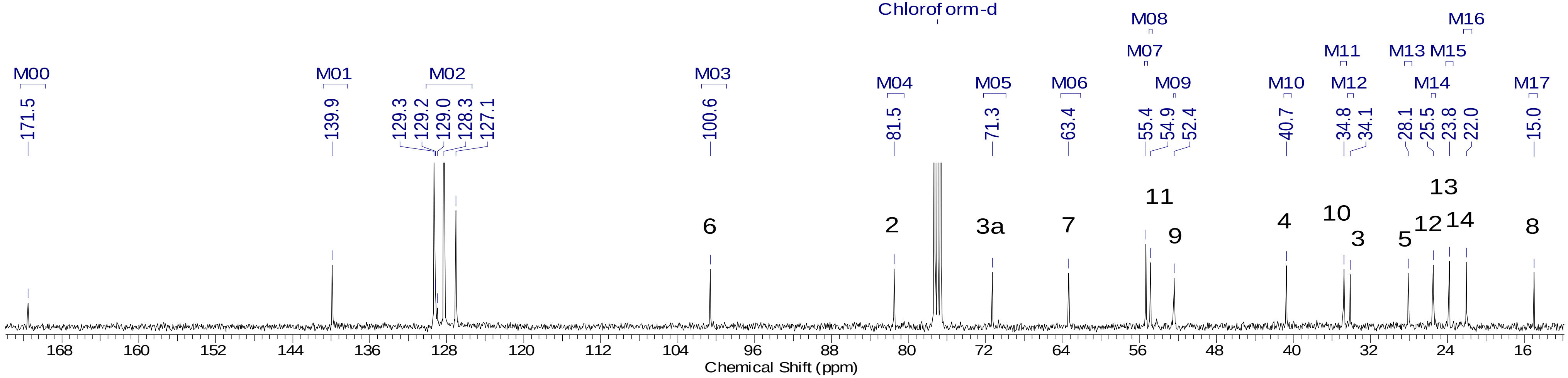


Spectrum 21 - $^{1}$H and $^{13}$C NMR of **9c’**

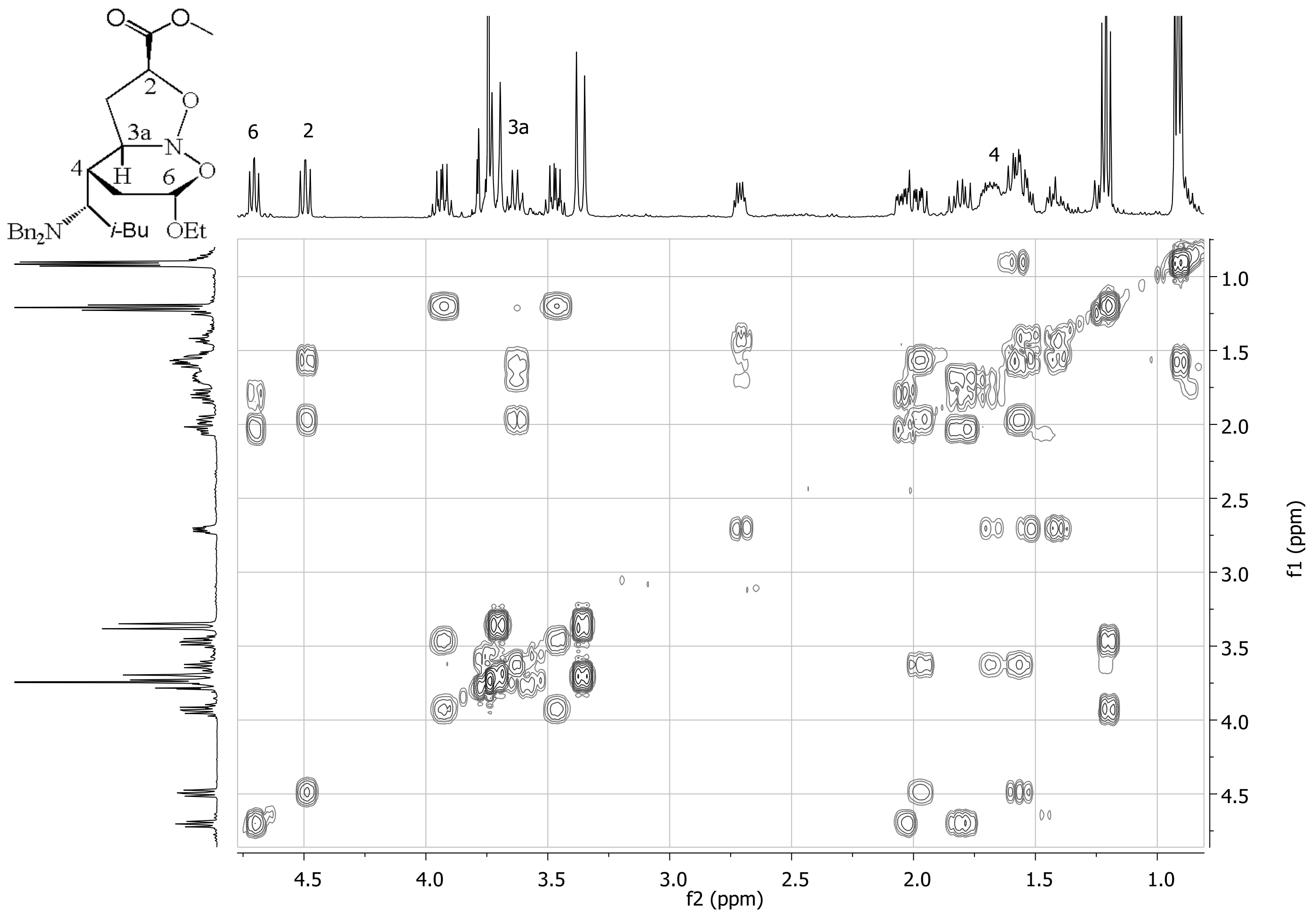


Spectrum 22 – 2D COSY of **9c'**

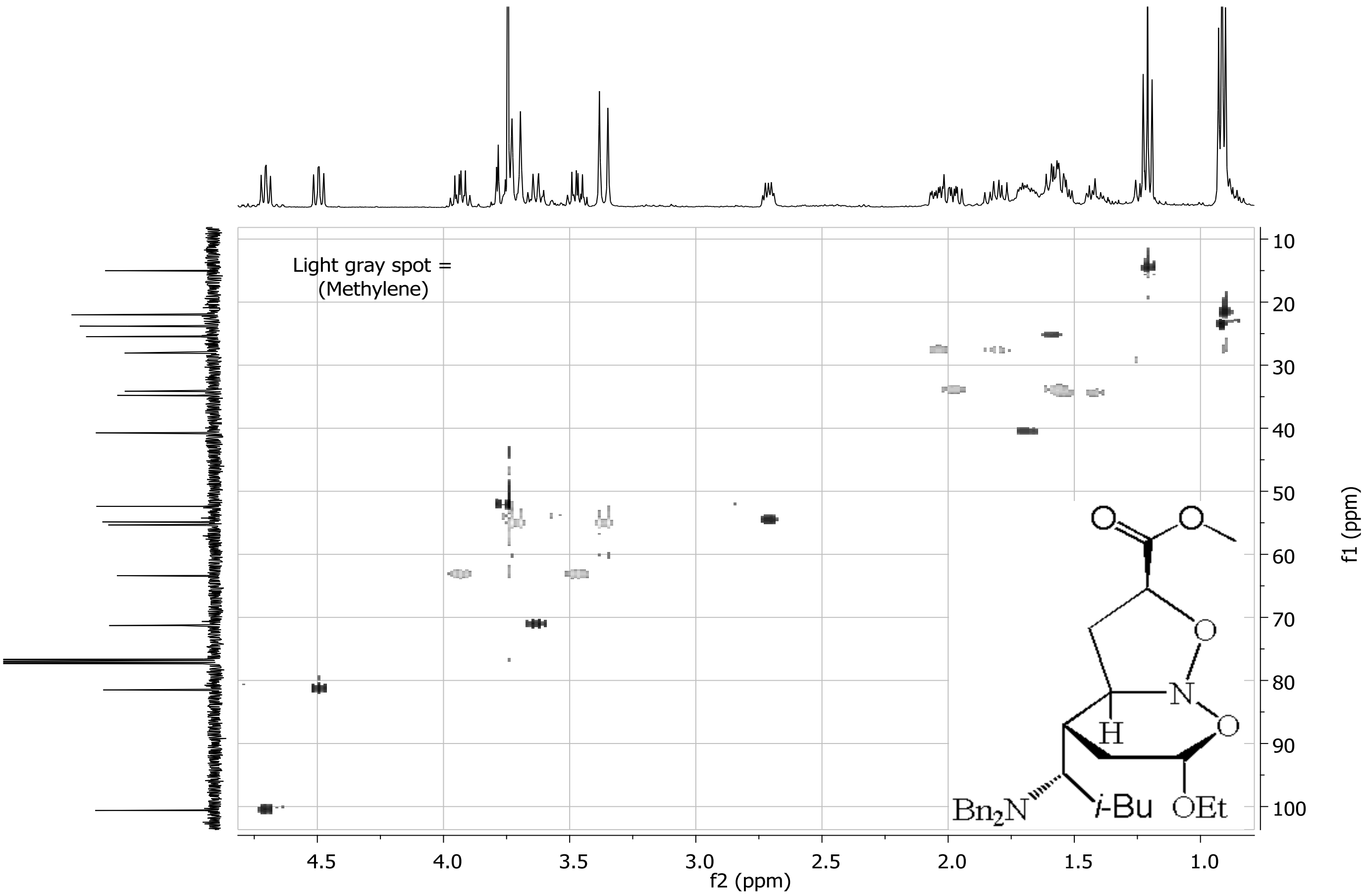


Spectrum 23 – HSQC of **9c'**

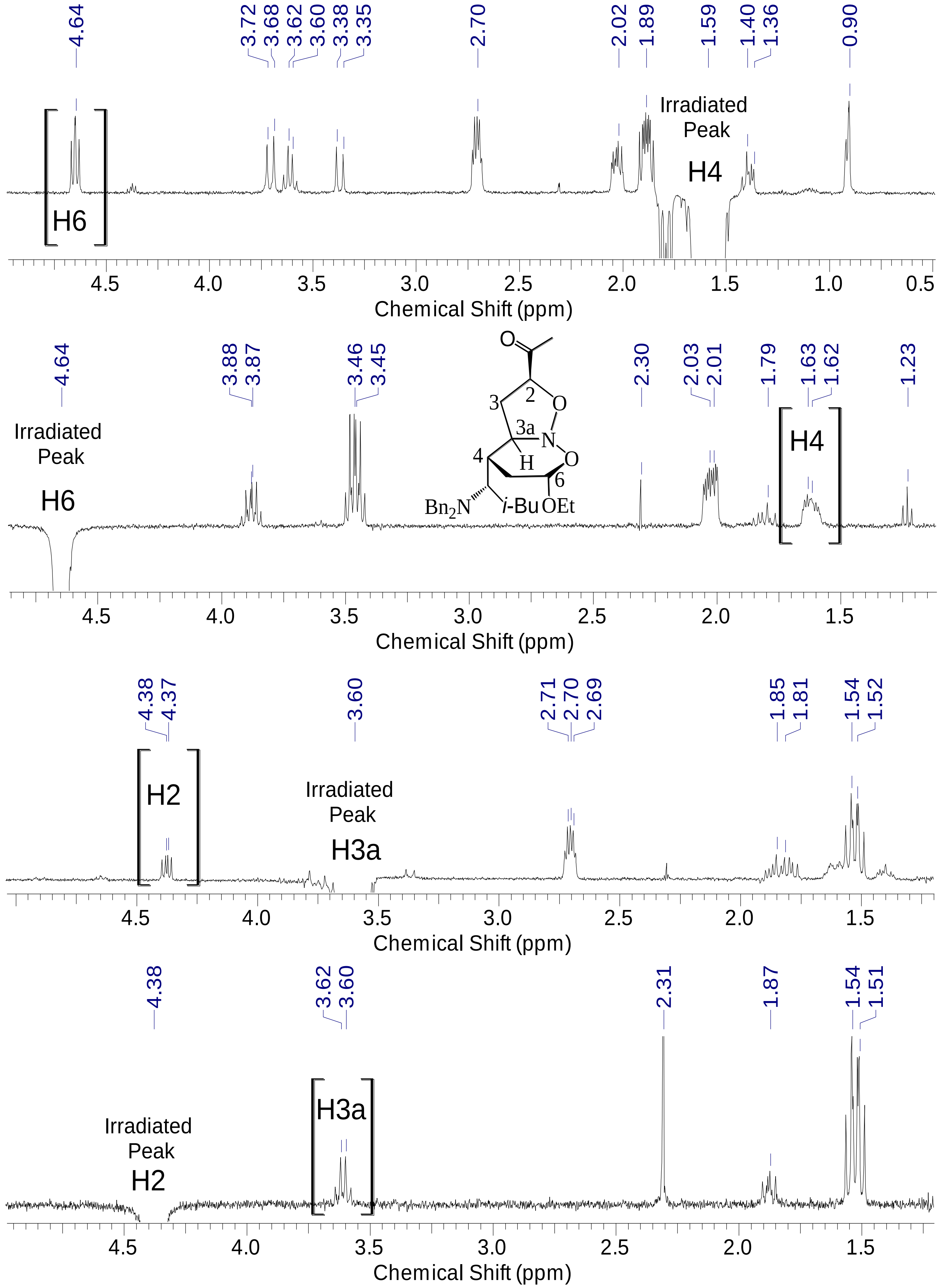


Spectrum 24 – 2D NOESY of **9c’**

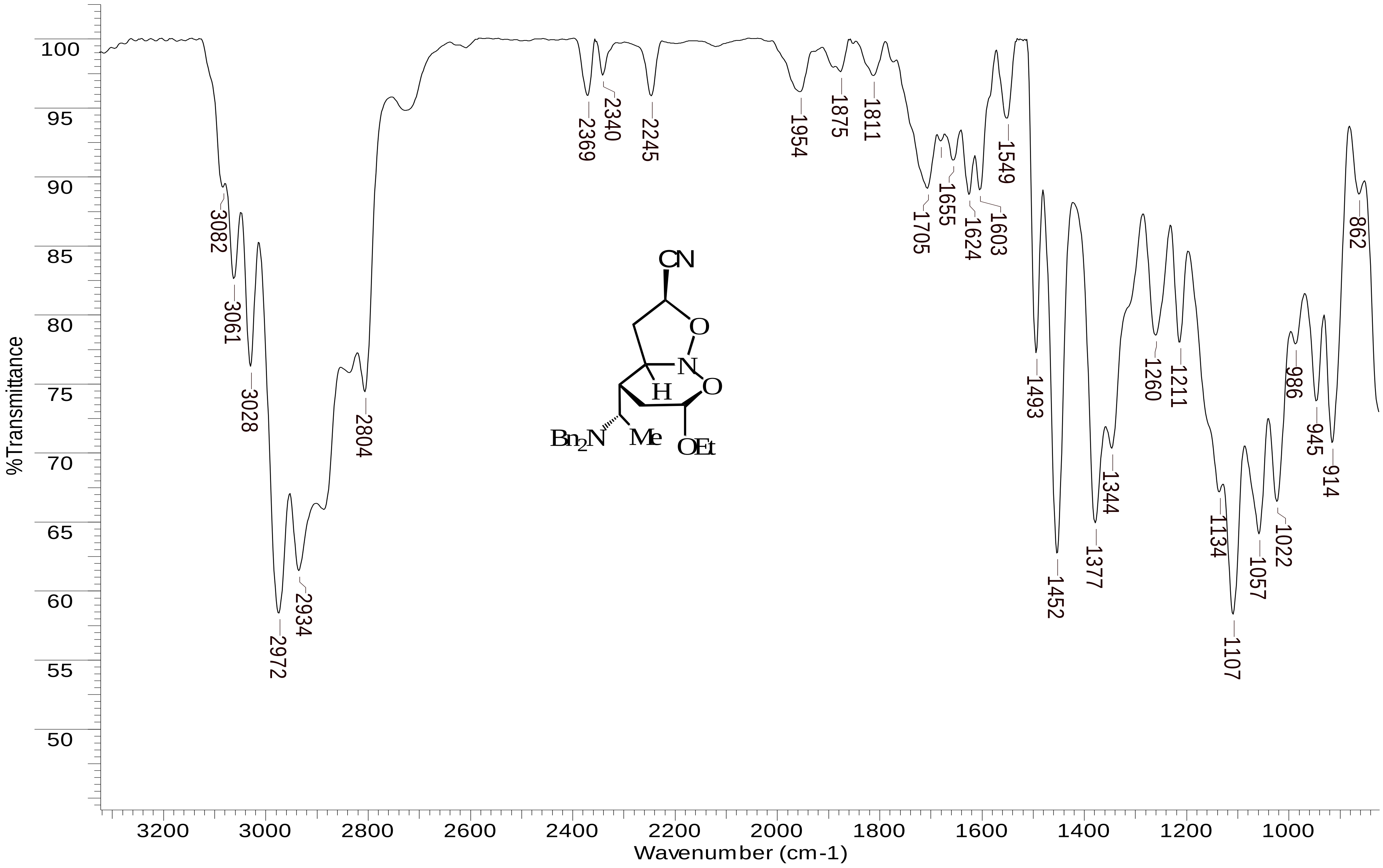


Spectrum 25 – Infrared of **10a’**

1H NMR (400 MHz, CHLOROFORM-D) δ ppm 1.05 (d, *J*=6.62 Hz, 3 H); 1.26 (t, *J*=6.95 Hz, 3 H); 1.55 (ddd, *J*=13.67, 13.67, 8.38 Hz, 1 H); 1.83 (m, 1 H); 2.32 (ddd, *J*=12.29, 9.10, 6.62 Hz, 1 H); 2.52 (m, 2 H); 2.70 (m, 1 H); 3.23 (m, 1 H); 3.32 (d, *J*=13.45 Hz, 2 H); 3.56 (m, 1 H); 3.75 (d, *J*=13.67 Hz, 2 H); 3.95 (m, 1 H); 4.81 (m, 2 H); 7.27 (m, 10 H)

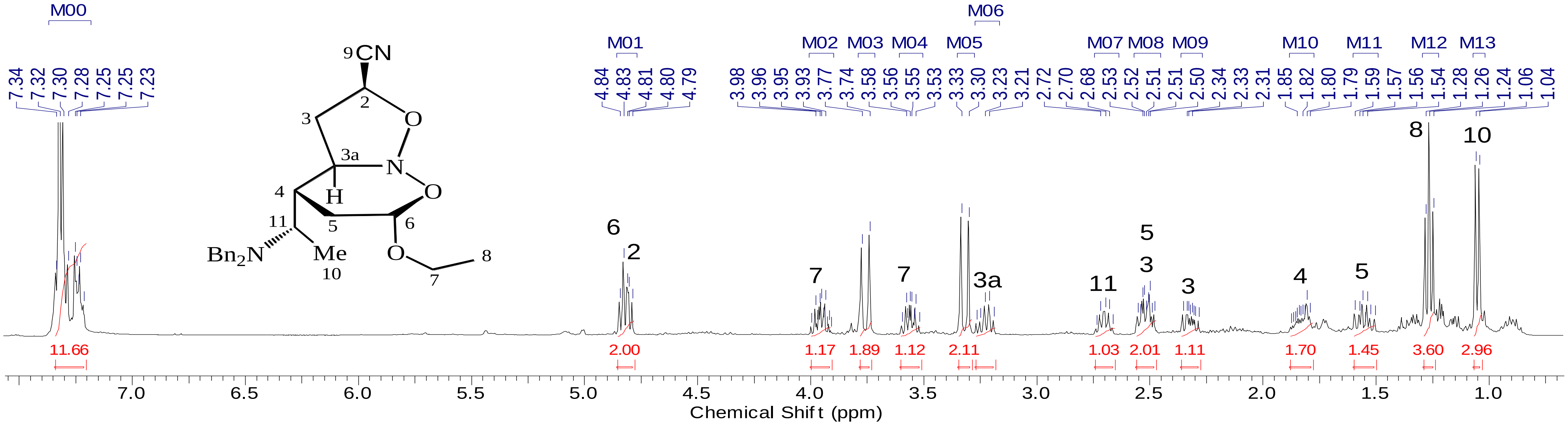


13C NMR (101 MHz, CHLOROFORM-D) δ ppm 9.9 ($CH_3$); 15.0 ($CH_3$); 29.3 ($CH_2$); 38.4 ($CH_2$); 42.8 (CH); 54.0 (2x $CH_2$); 55.0 (CH); 63.6 ($CH_2$); 68.8 (CH); 72.9 (CH); 100.7 (CH); 118.6 (CN); 127.1-128.8 (10x $CH_{Ar}$); 139.3 (2x $C_{ipso}$)

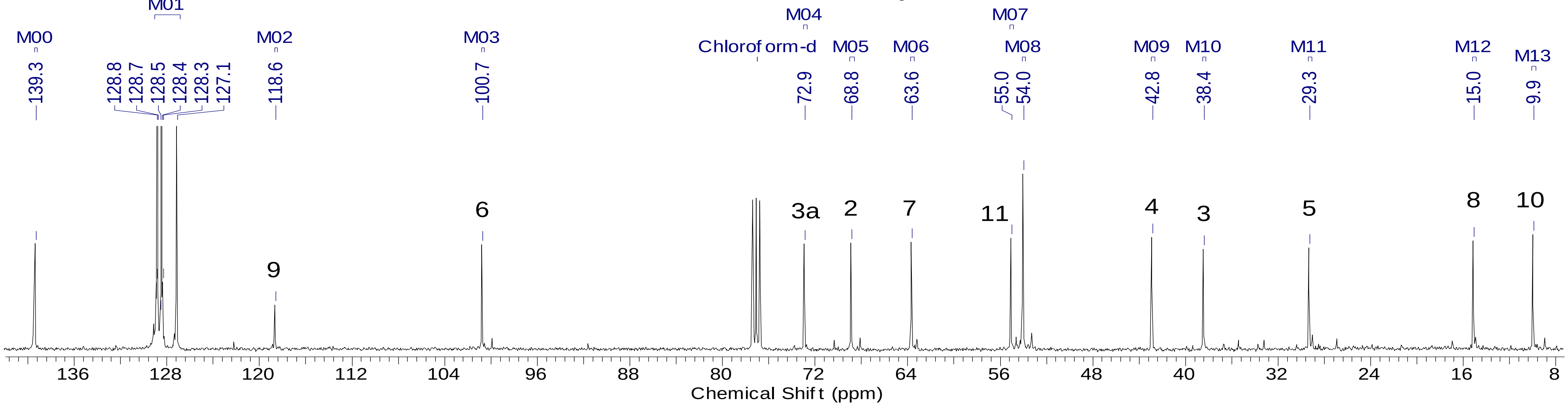


Spectrum 26 - ${}^{1}$H and ${}^{13}$C NMR of **10a’**

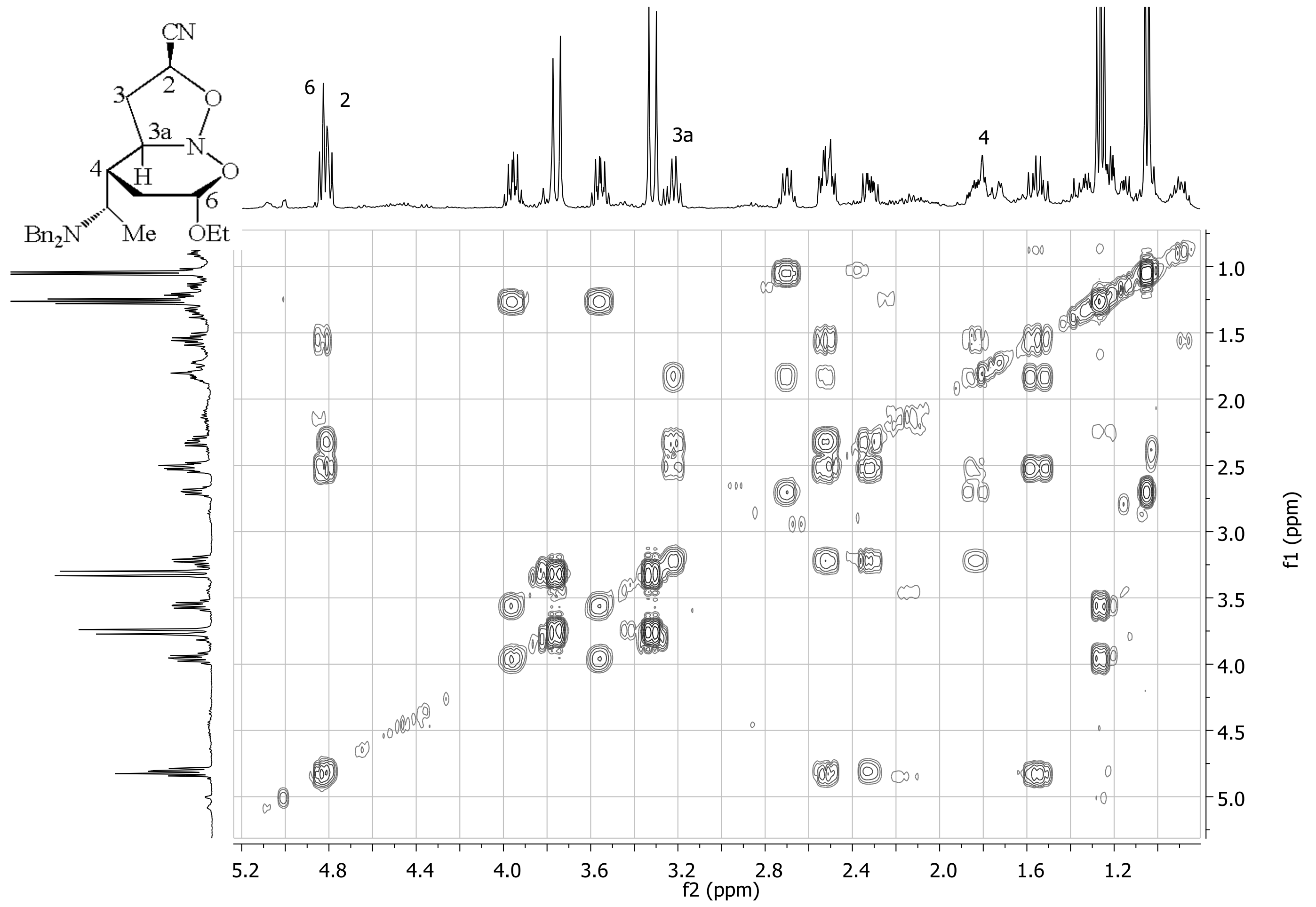


Spectrum 27 – 2D COSY of **10a’**

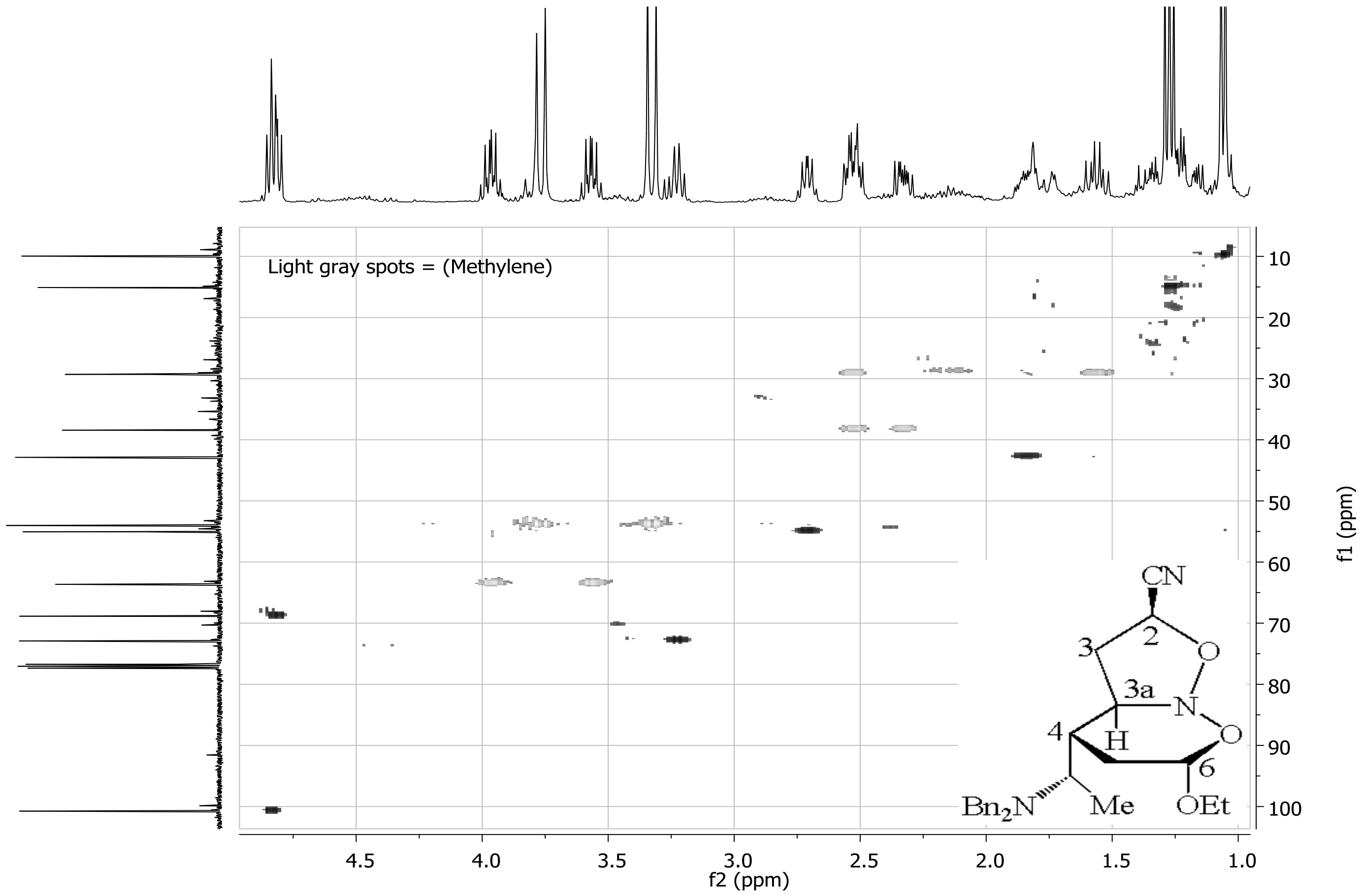


Spectrum 28 – HSQC of **10a’**

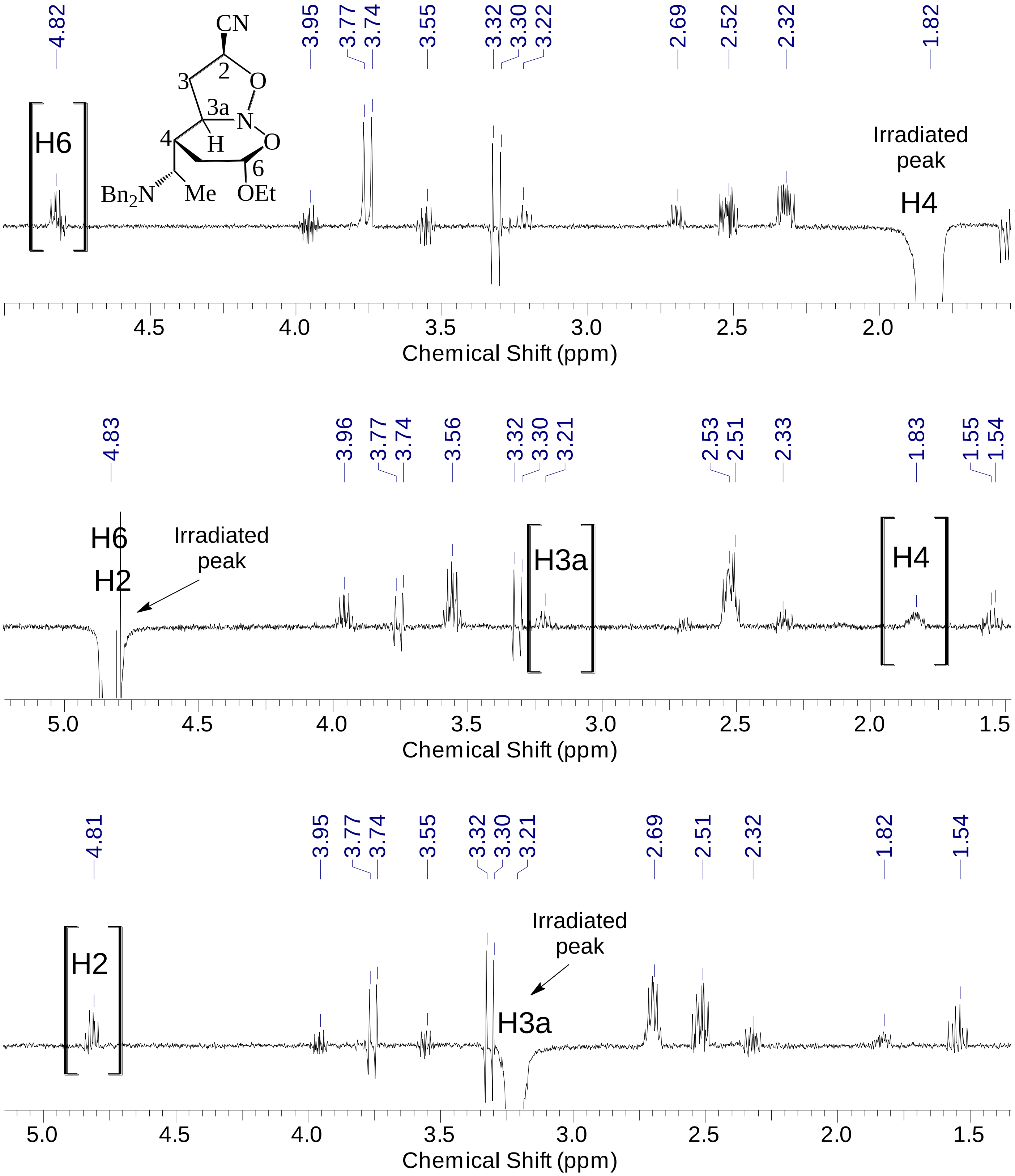


Spectrum 29 – 2D NOESY of **10a’**

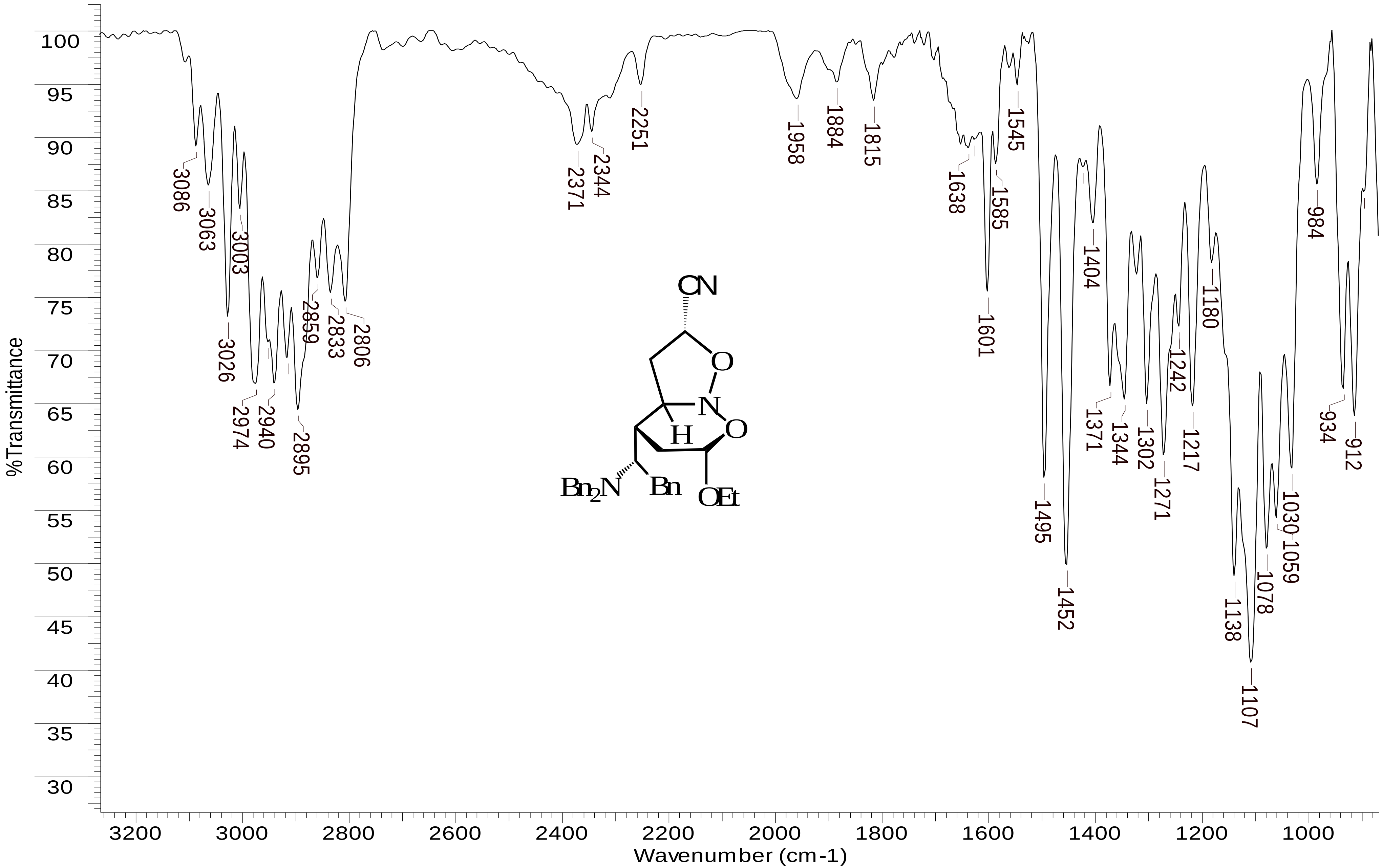


Spectrum 30 – Infrared of **11b**

1H NMR (500 MHz, CHLOROFORM-D) δ ppm 1.21 (t, *J*=7.09 Hz, 3 H); 1.26 (ddd, *J*=12.10, 8.44, 4.89 Hz, 1 H); 1.38 (m, 1 H); 1.48 (ddd, *J*=12.04, 9.38, 9.38 Hz, 1 H); 1.88 (ddd, *J*=13.63, 13.63, 8.44 Hz, 1 H); 2.19 (ddd, *J*=12.96, 6.48, 2.57 Hz, 1 H); 2.70 (dd, *J*=13.33, 10.88 Hz, 1 H); 2.92 (ddd, *J*=10.76, 3.91, 1 H); 3.25 (dd, *J*=13.45, 3.91 Hz, 1 H); 3.44 (m, 1 H); 3.43 (d, *J*=13.45 Hz, 2 H); 3.86 (m, 4 H); 4.58 (dd, *J*=8.31, 6.60 Hz, 1 H); 4.83 (dd, *J*=9.78, 4.89 Hz, 1 H); 7.29 (m, 15 H)

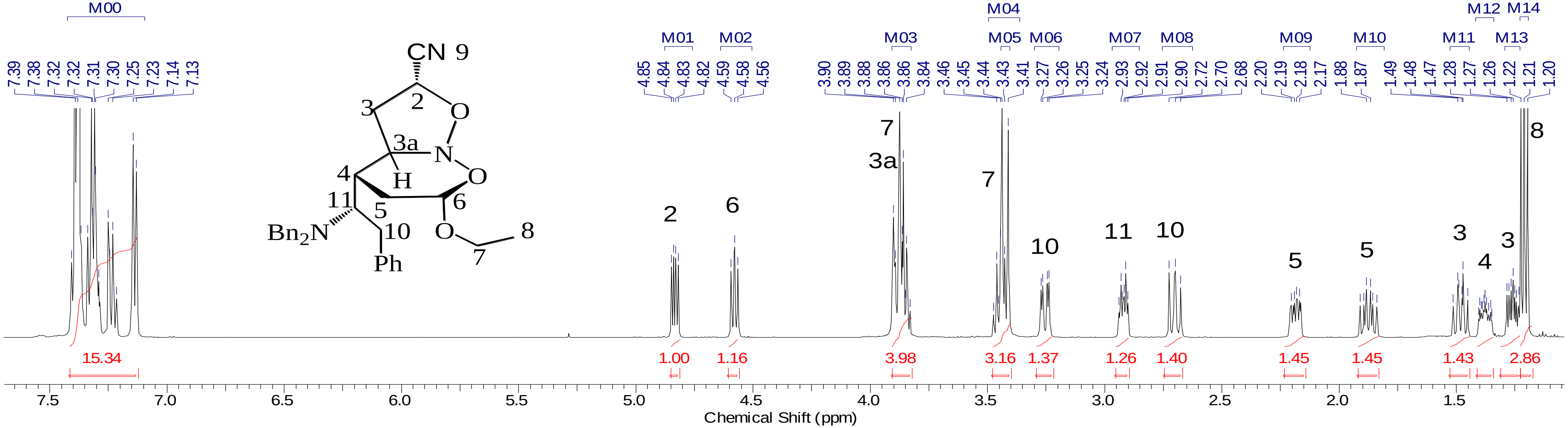


13C NMR (126 MHz, CHLOROFORM-D) δ ppm 15.0 ($CH_3$); 27.2 ($CH_2$); 31.3 ($CH_2$); 36.0 ($CH_2$); 40.0 (CH); 55.8 (2x $CH_2$); 58.7 (CH); 63.7 ($CH_2$); 69.0 (CH); 71.4 (CH); 100.7 (CH); 116.6 (CN); 126.4-129.2 (15 $CH_{Ar}$); 139.2 (2x $C_{ipso}$); 139.7 ($C_{ipso}$)

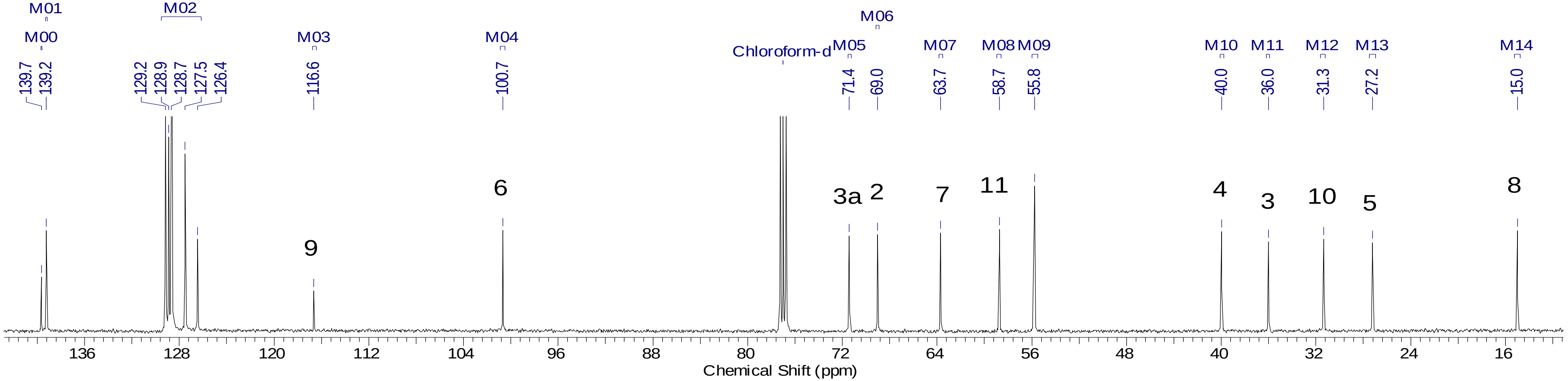


Spectrum 31 - $^{1}$H and $^{13}$C NMR of **11b**

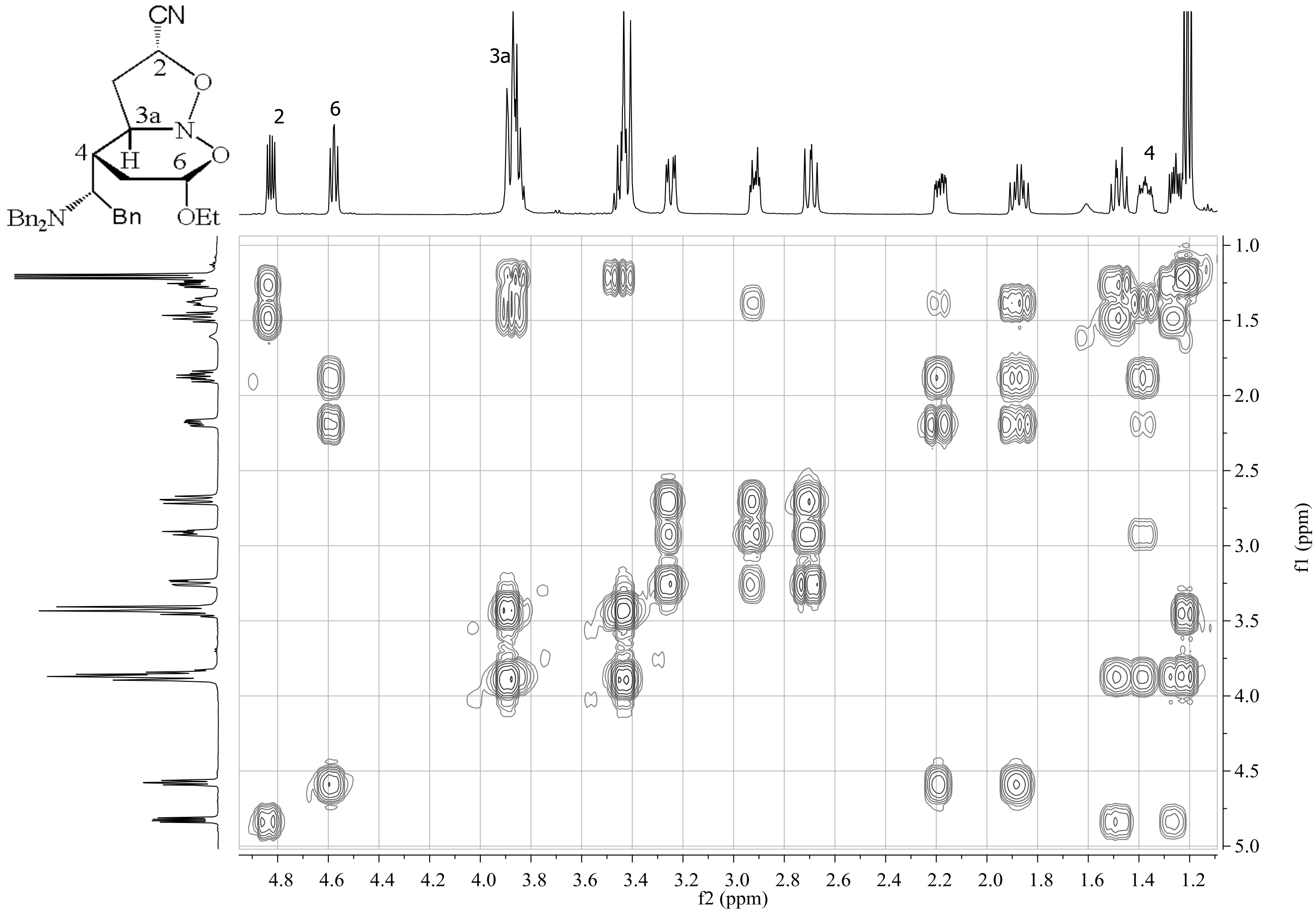


Spectrum 32 – 2D COSY of **11b**

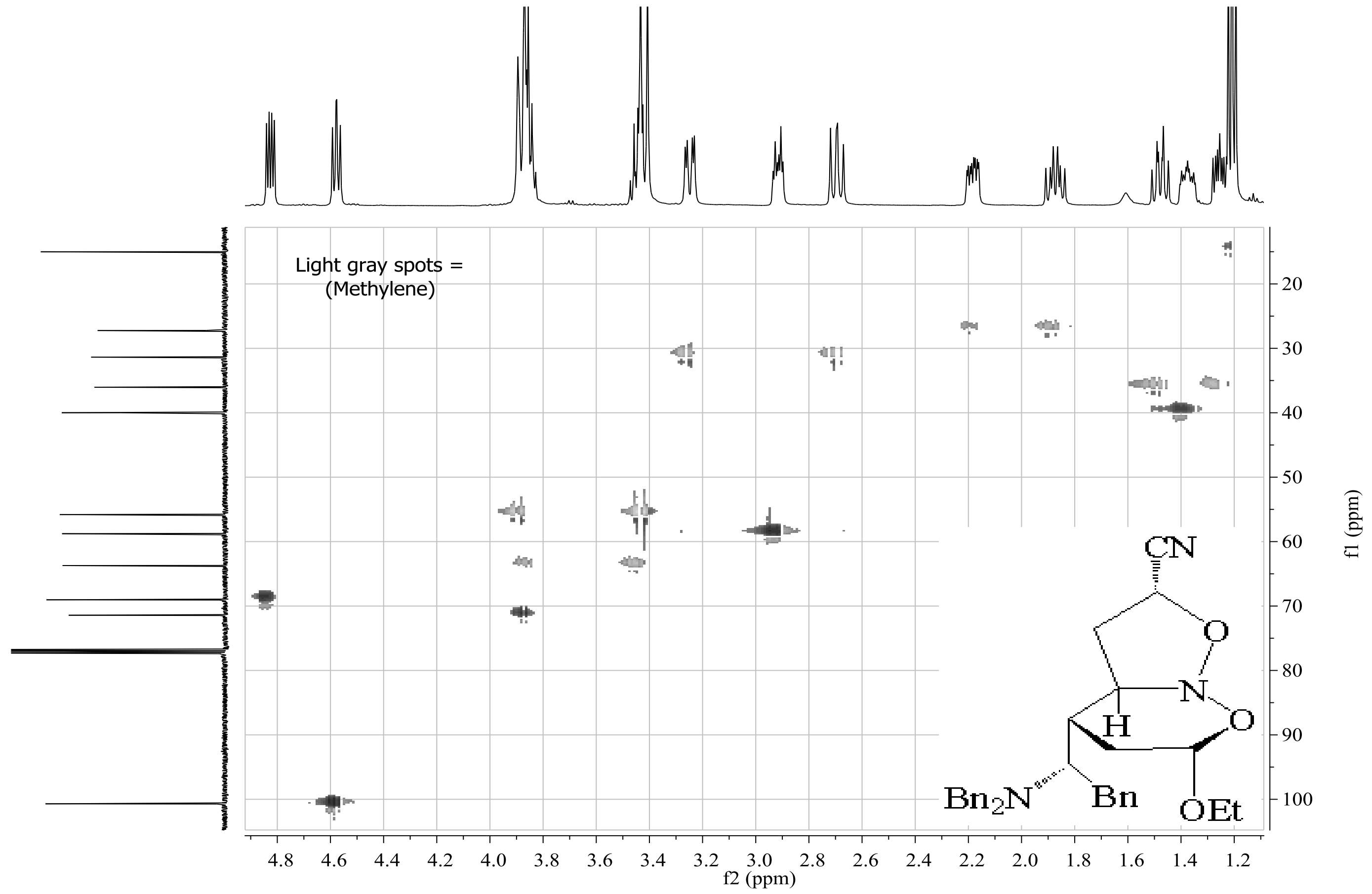


Spectrum 33 – HSQC of **11b**

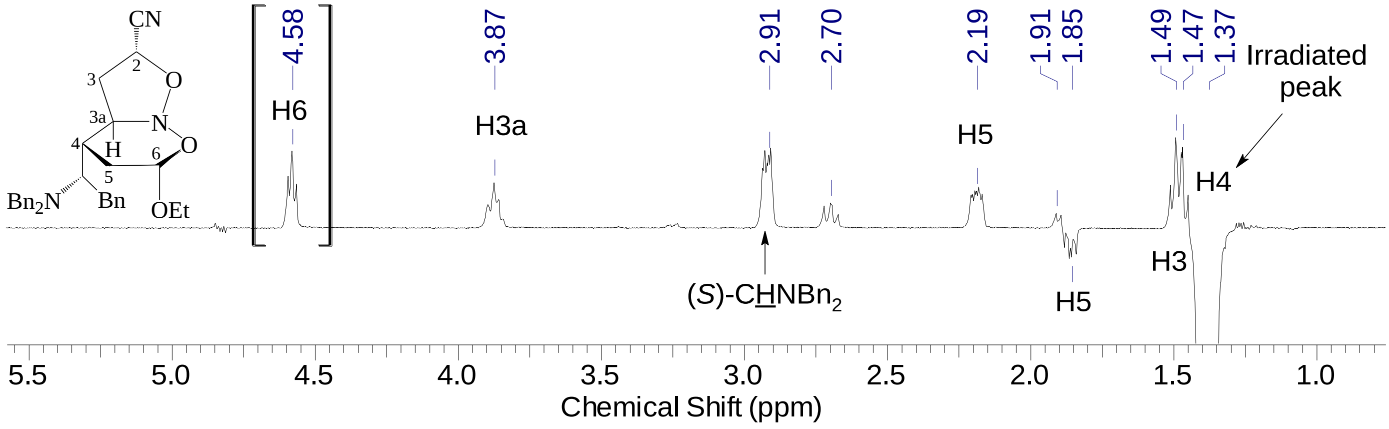


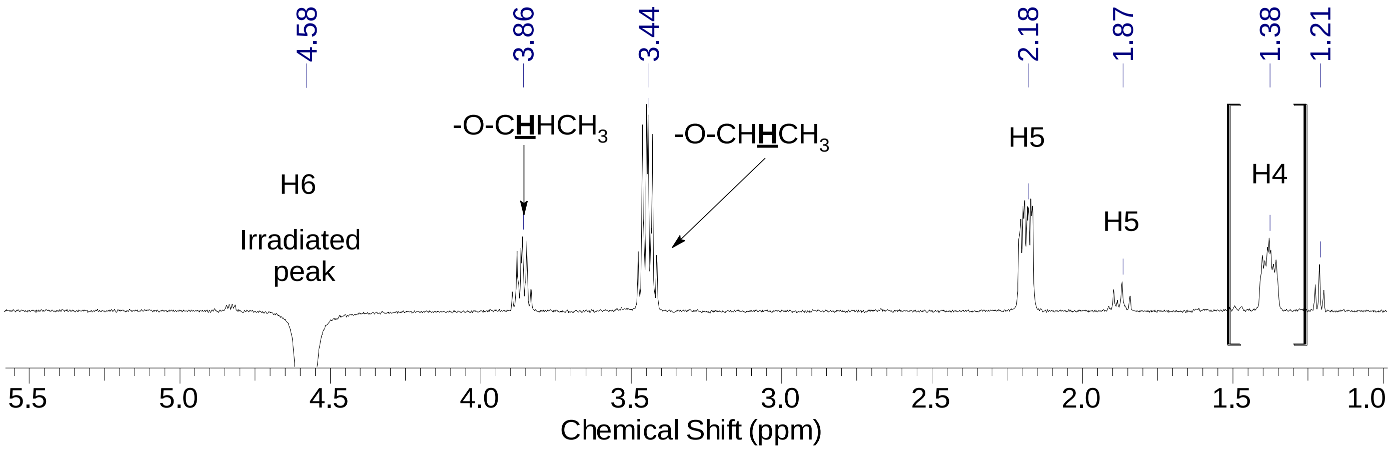

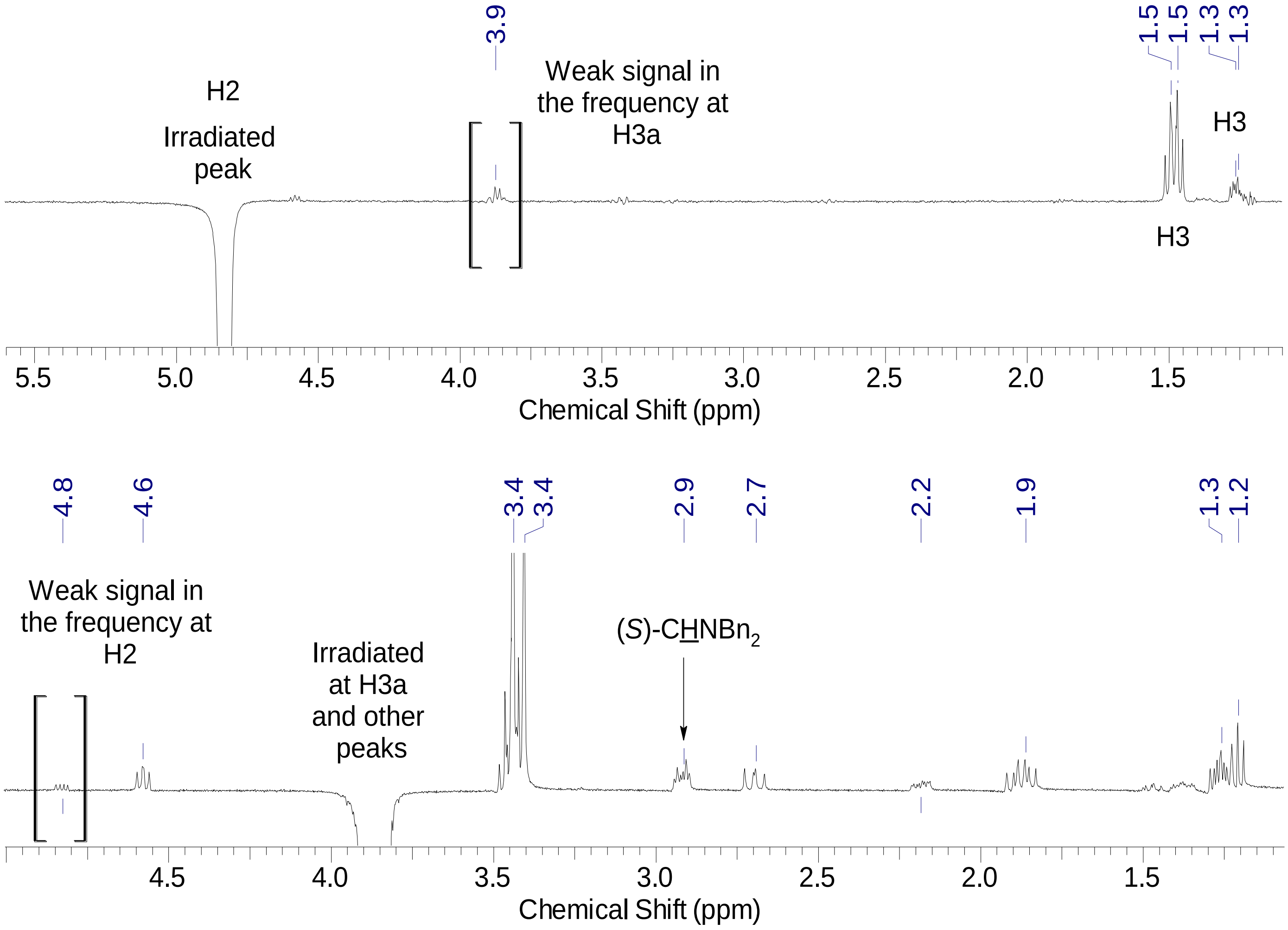


Spectrum 34 – 2D NOESY of **11b**

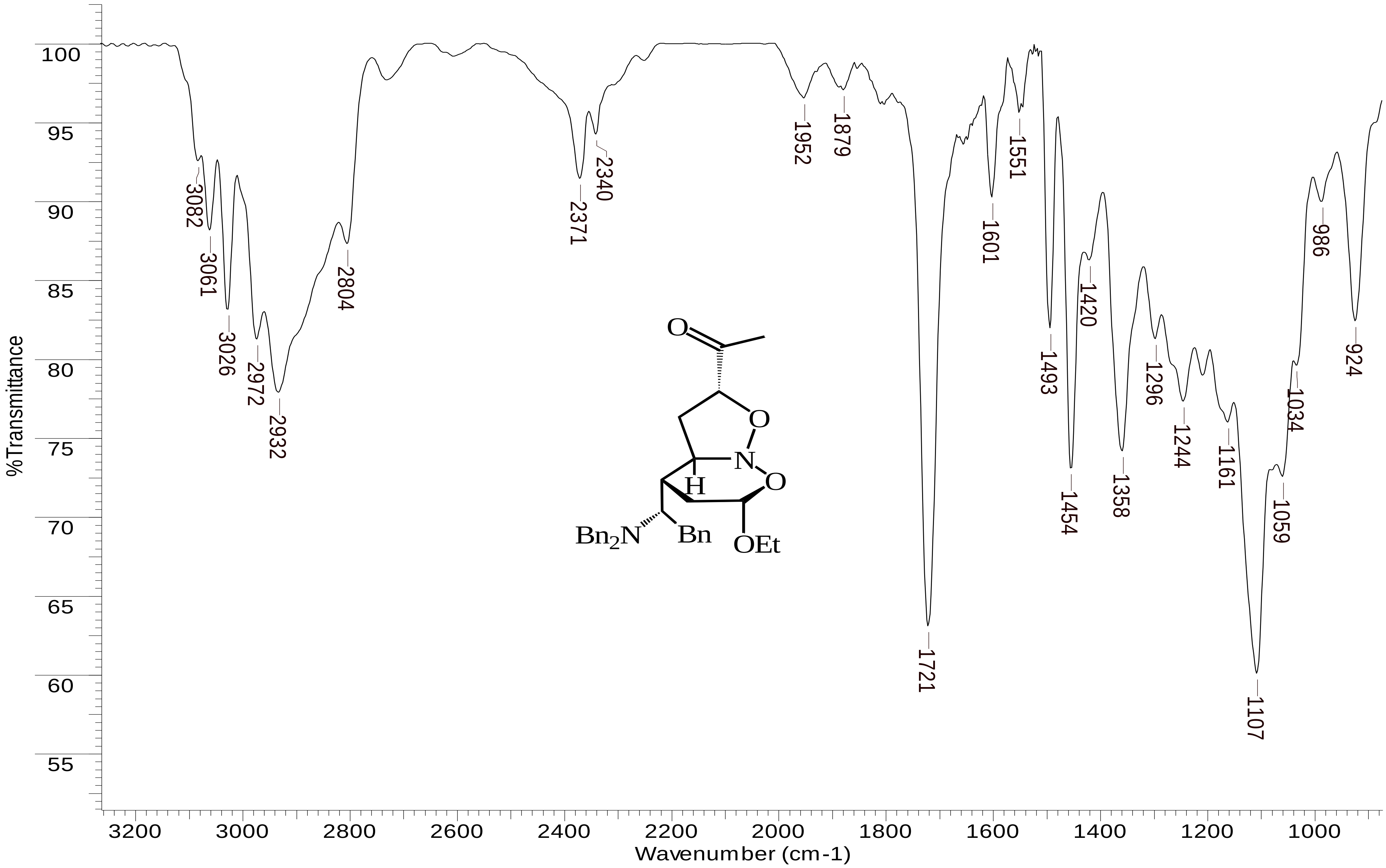


Spectrum 35 – Infrared of **12b**

1H NMR (400 MHz, CHLOROFORM-D) δ ppm 1.10 (ddd, *J*=12.18, 8.77, 5.73 Hz, 1 H); 1.23 (t, *J*=7.06 Hz, 3 H); 1.42 (m, 1 H); 1.47 (m, 1 H); 1.86 (m, 1 H); 2.06 (s, 3 H); 2.22 (ddd, *J*=12.84, 6.23, 2.98 Hz, 1 H); 2.69 (dd, *J*=13.45, 10.80 Hz, 1 H); 2.93 (ddd, *J*=10.69, 3.86, 3.75 Hz, 1 H); 3.25 (dd, *J*=13.45, 3.75 Hz, 1 H); 3.42 (d, *J*=13.45 Hz, 2 H); 3.47 (ddd, *J*=7.06, 7.06, 2.43 Hz, 1 H); 3.54 (m, 1 H); 3.87 (d, *J*=13.89 Hz, 2 H); 3.91 (m, 1 H); 4.61 (dd, *J*=10.47, 5.62 Hz, 1 H); 4.64 (dd, *J*=8.05, 6.73 Hz, 1 H); 7.27 (m, 15 H)

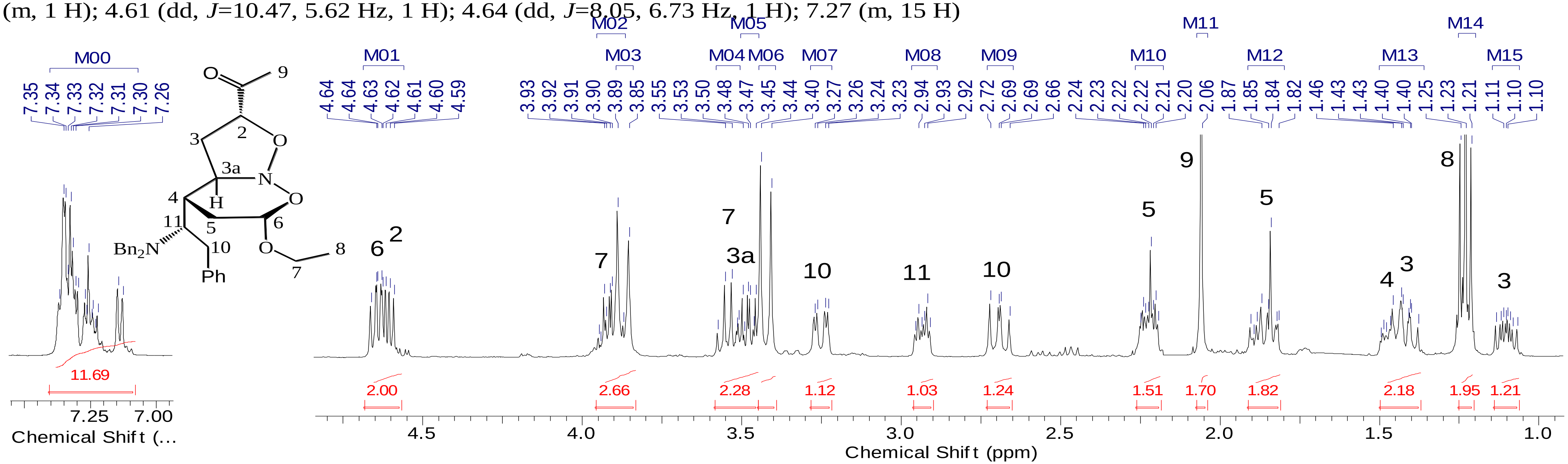


13C NMR (101 MHz, CHLOROFORM-D) δ ppm 15.0 ($CH_3$); 26.2 ($CH_3$); 27.6 ($CH_2$); 31.6 ($CH_2$); 34.6 ($CH_2$); 40.3 (CH); 55.7 (2x $CH_2$); 59.3 (CH); 63.5 ($CH_2$); 70.7 (CH); 87.2 (CH); 100.4 (CH); 126.4-129.2 (15x $CH_{Ar}$); 139.4 (2x $C_{ipso}$); 139.8 ($C_{ipso}$); 207.0 (C=O)

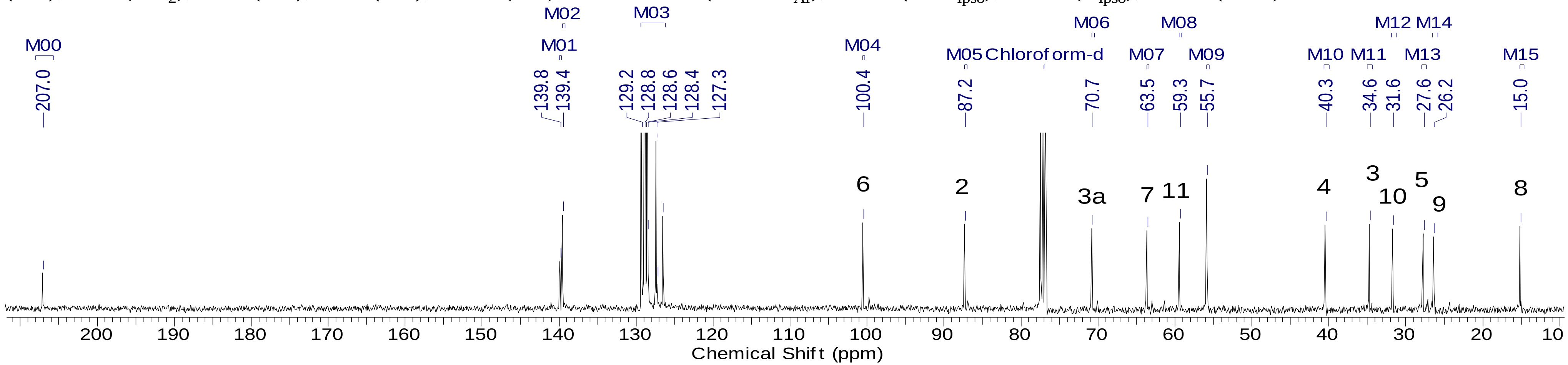


Spectrum 36 - $^1$H and $^{13}$C NMR of **12b**

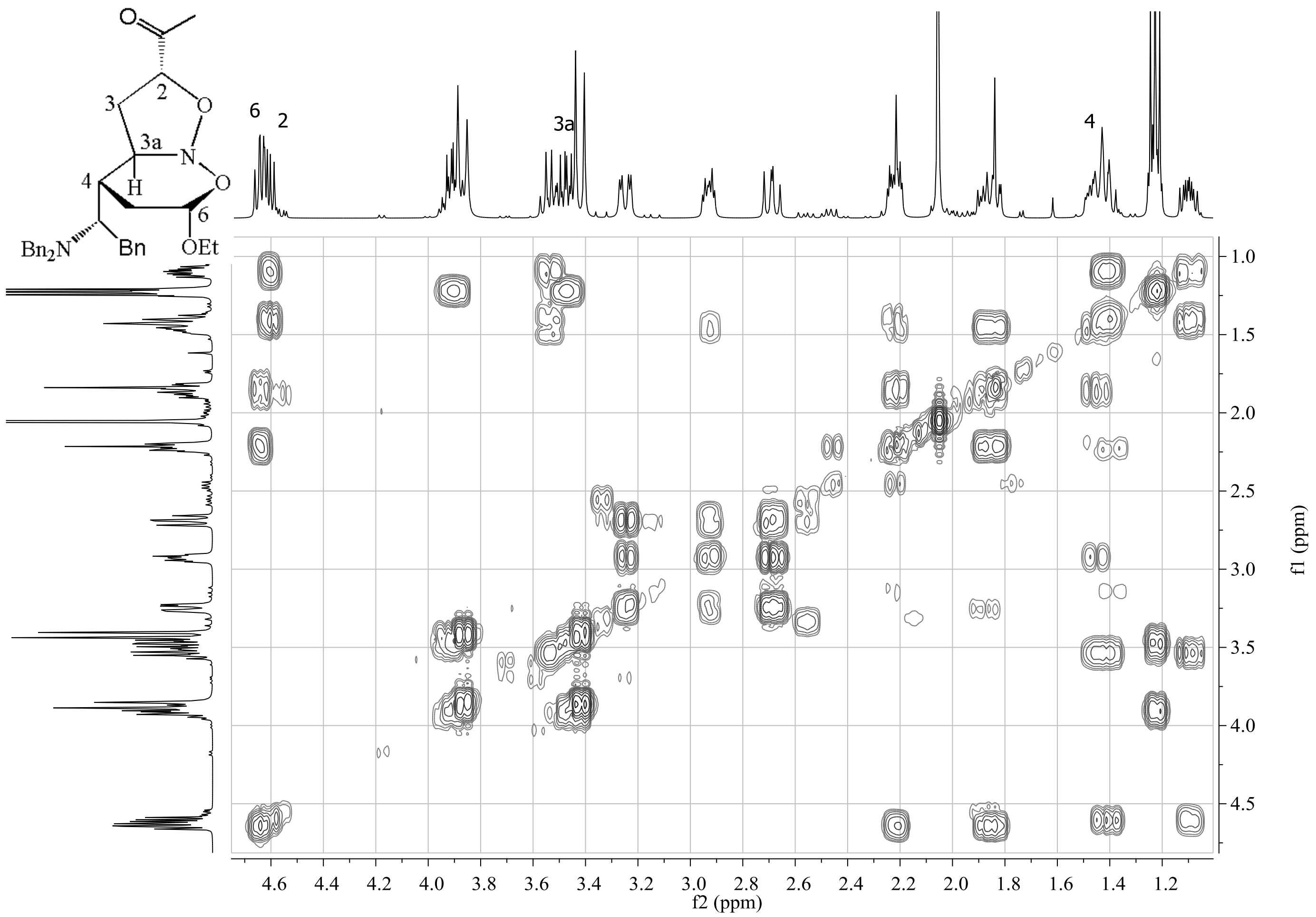


Spectrum 37 – 2D COSY of **12b**

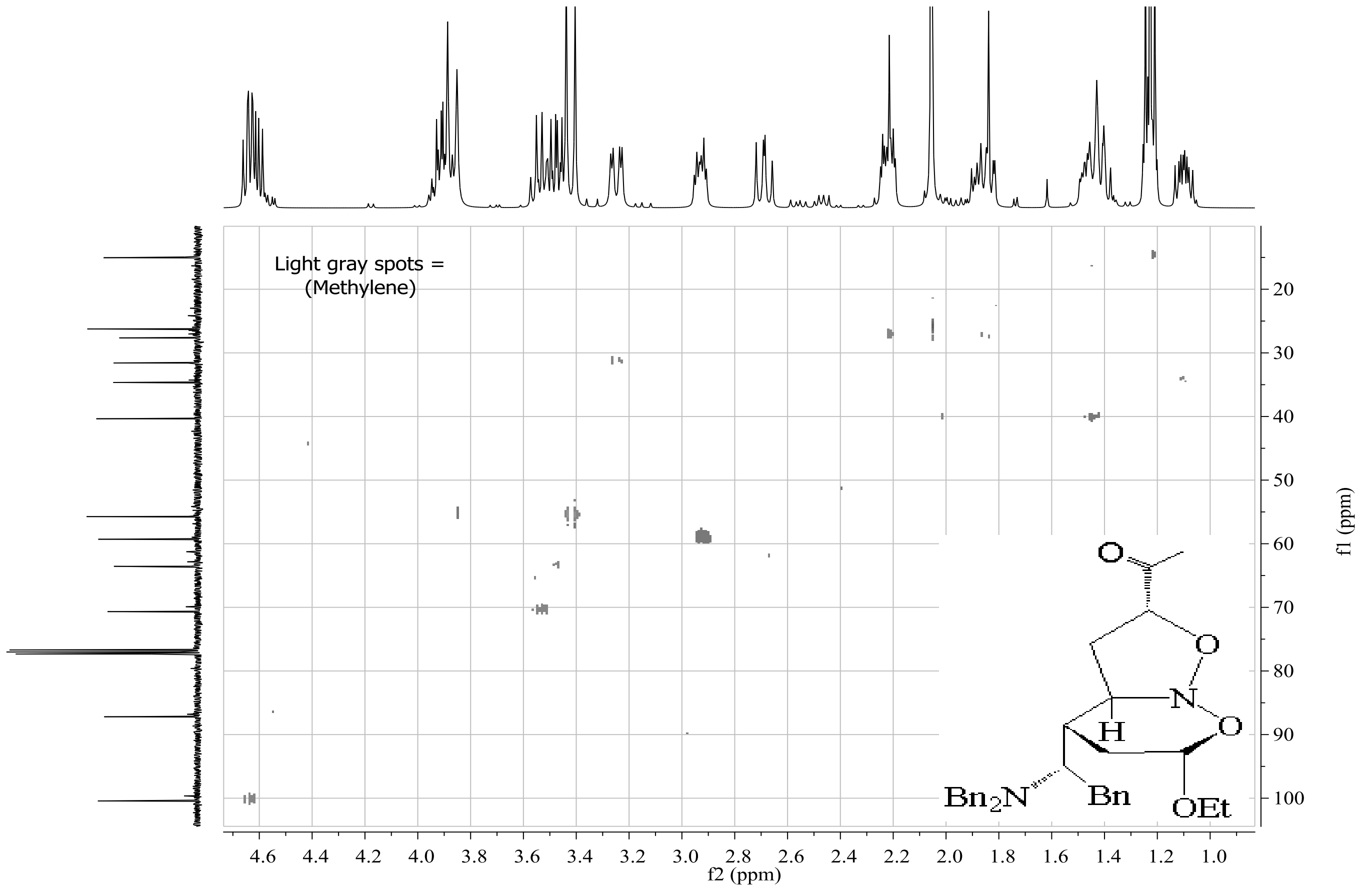


Spectrum 38 – HSQC of **12b**

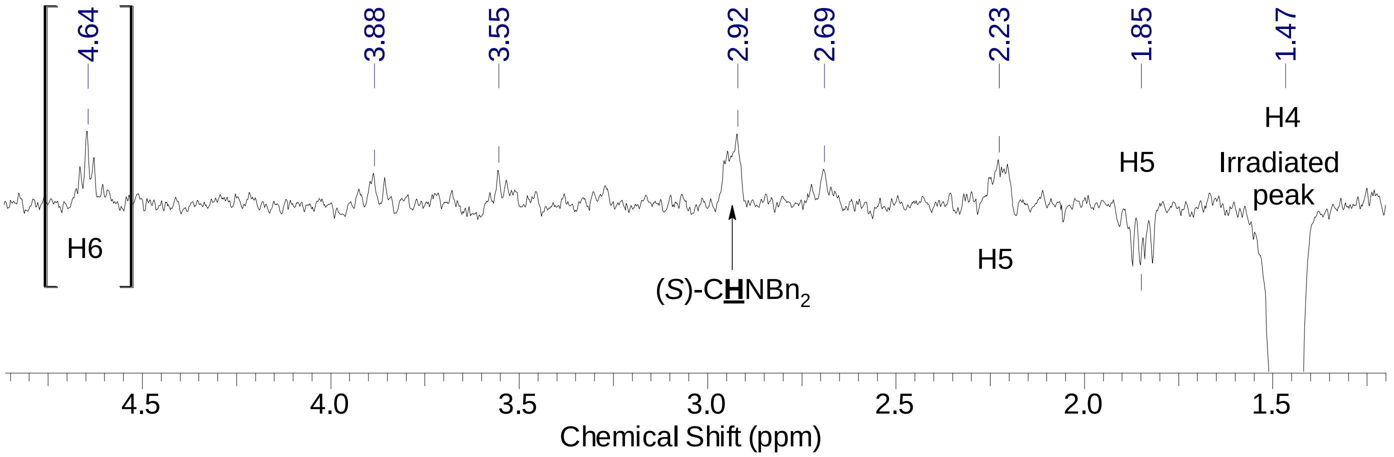


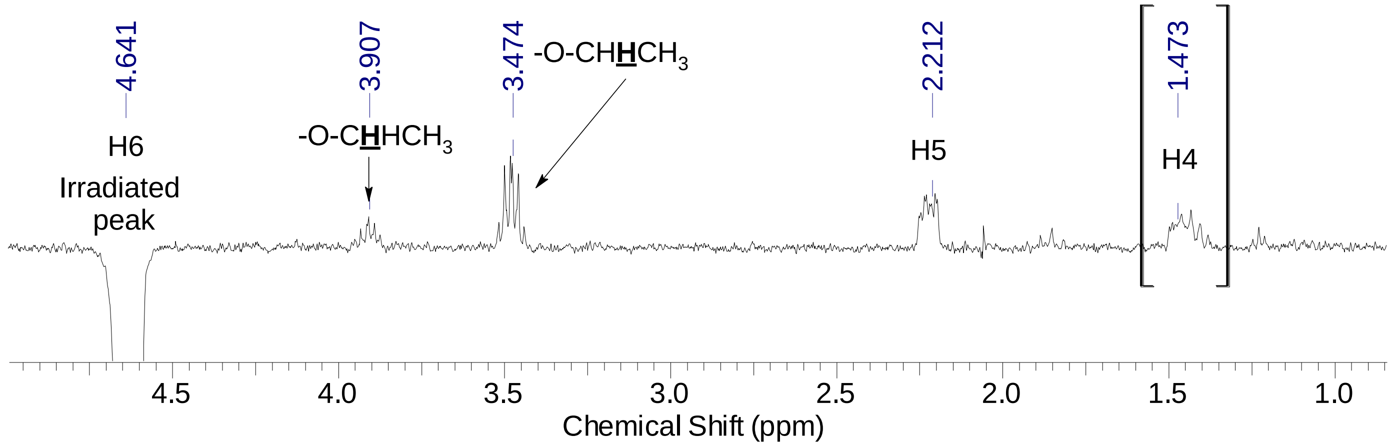

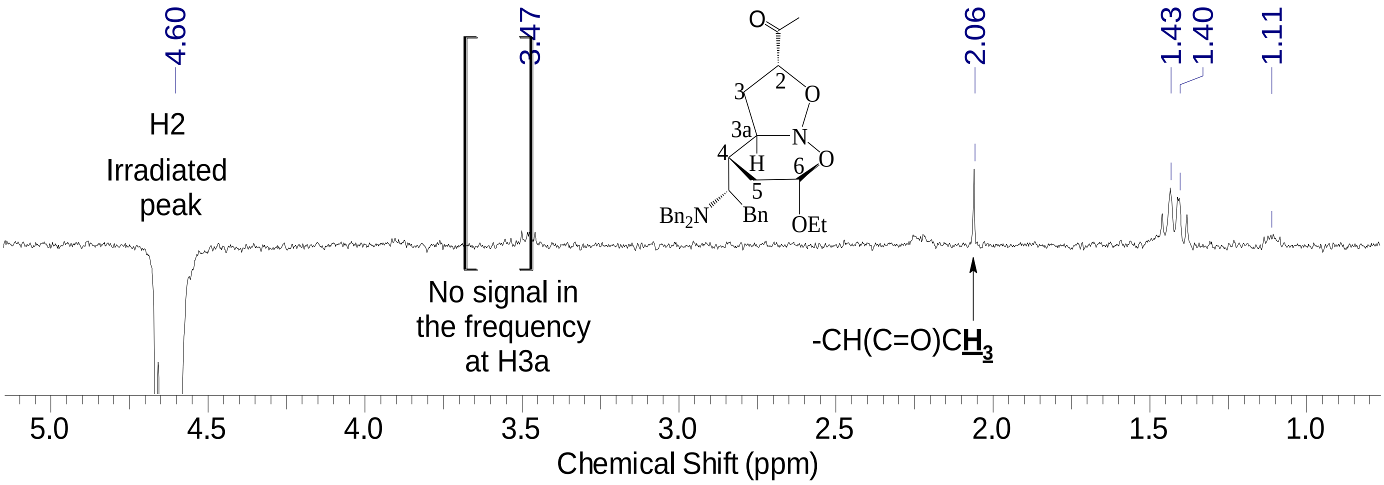


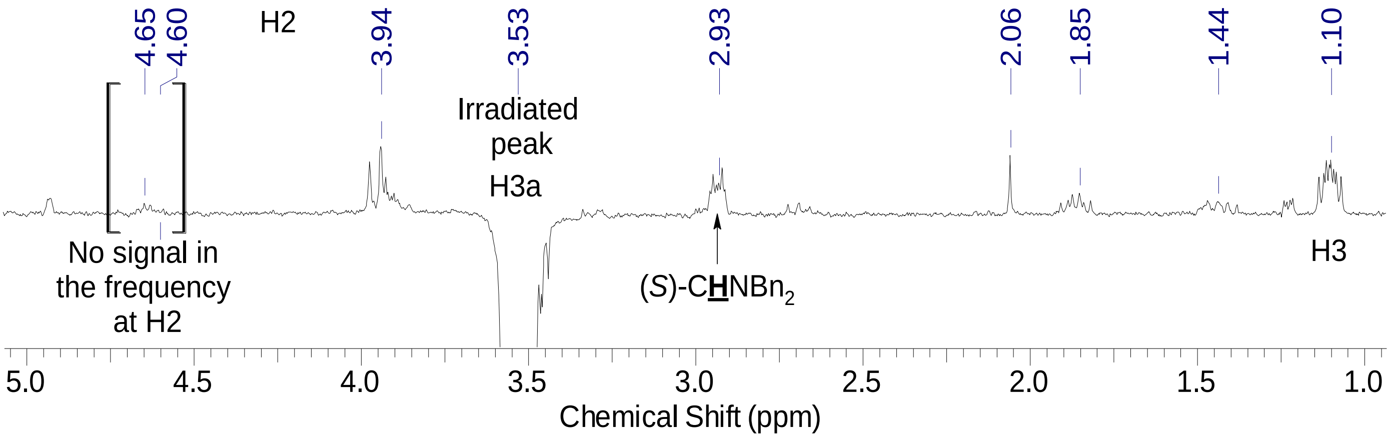


Spectrum 39 – 2D NOESY of **12b**

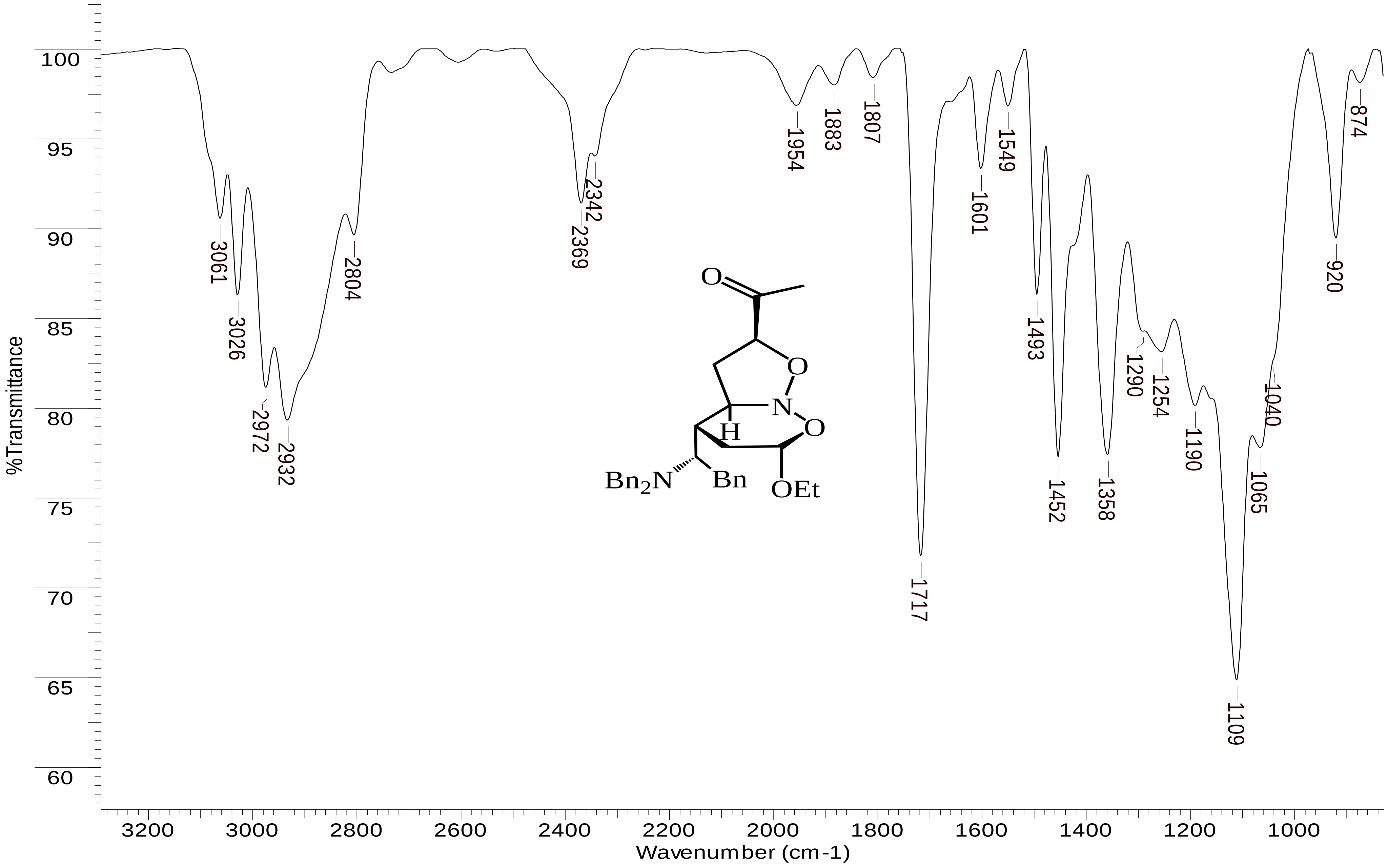


Spectrum 40 – Infrared of **12b’**

1H NMR (400 MHz, CHLOROFORM-D) δ ppm 1.23 (t, *J*=7.06 Hz, 3 H); 1.42 (m, 1 H); 1.49 (m, 1 H); 1.58 (ddd, *J*=12.02, 9.04, 6.51 Hz, 1 H); 1.84 (ddd, *J*=13.51, 13.51, 8.27 Hz, 1 H); 2.20 (m, 1 H); 2.23 (s, 3 H); 2.68 (dd, *J*=13.67, 9.70 Hz, 1 H); 2.98 (ddd, *J*=9.54, 4.82 Hz, 1 H); 3.21 (dd, *J*=13.78, 4.74 Hz, 1 H); 3.41 (d, *J*=13.45 Hz, 2 H); 3.46 (m, 1 H); 3.55 (m, 1 H); 3.84 (d, *J*=12.57 Hz, 2 H); 3.86 (m, 1 H); 4.28 (dd, *J*=9.70, 6.39 Hz, 1 H); 4.56 (dd, *J*=8.27, 6.50 Hz, 1 H); 7.24 (m, 15 H)

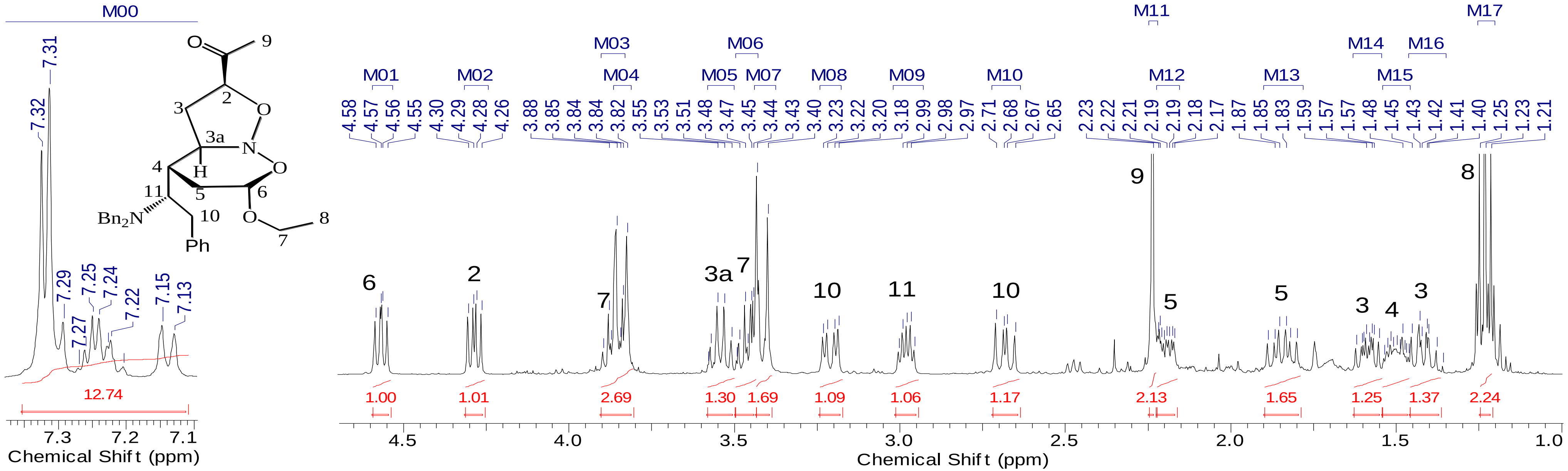


13C NMR (101 MHz, CHLOROFORM-D) δ ppm 15.0 (CH$_3$); 26.8 (CH$_3$); 27.9 (CH$_2$); 32.1 (CH$_2$); 32.6 (CH$_2$); 40.5 (CH); 55.5 (2x CH$_2$); 59.3 (CH); 63.4 (CH$_2$); 71.2 (CH); 88.6 (CH); 100.5 (CH); 126.2-129.3 (15x CH$_{Ar}$); 139.4 (2x C$_{ipso}$); 139.8 (C$_{ipso}$); 209.1 (C=O)

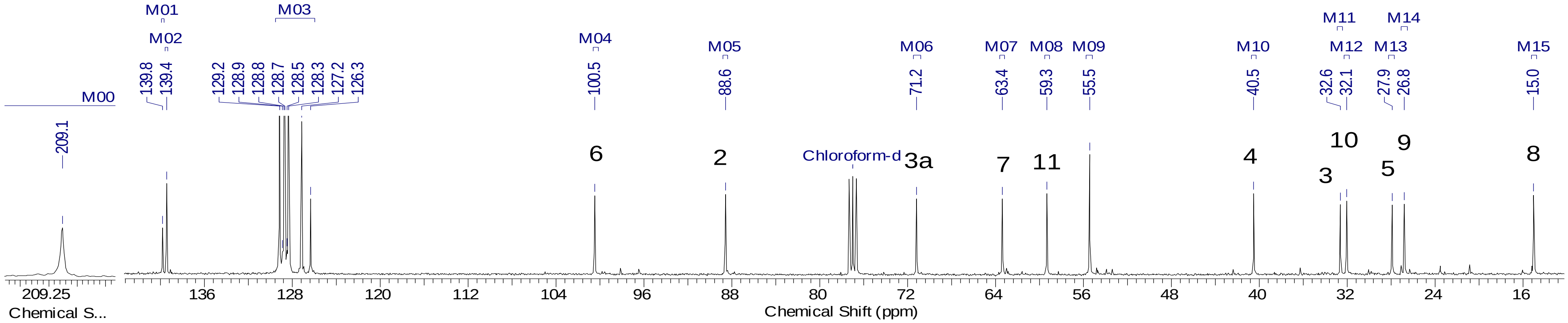


Spectrum 41 - $^{1}$H and $^{13}$C NMR of **12b'**

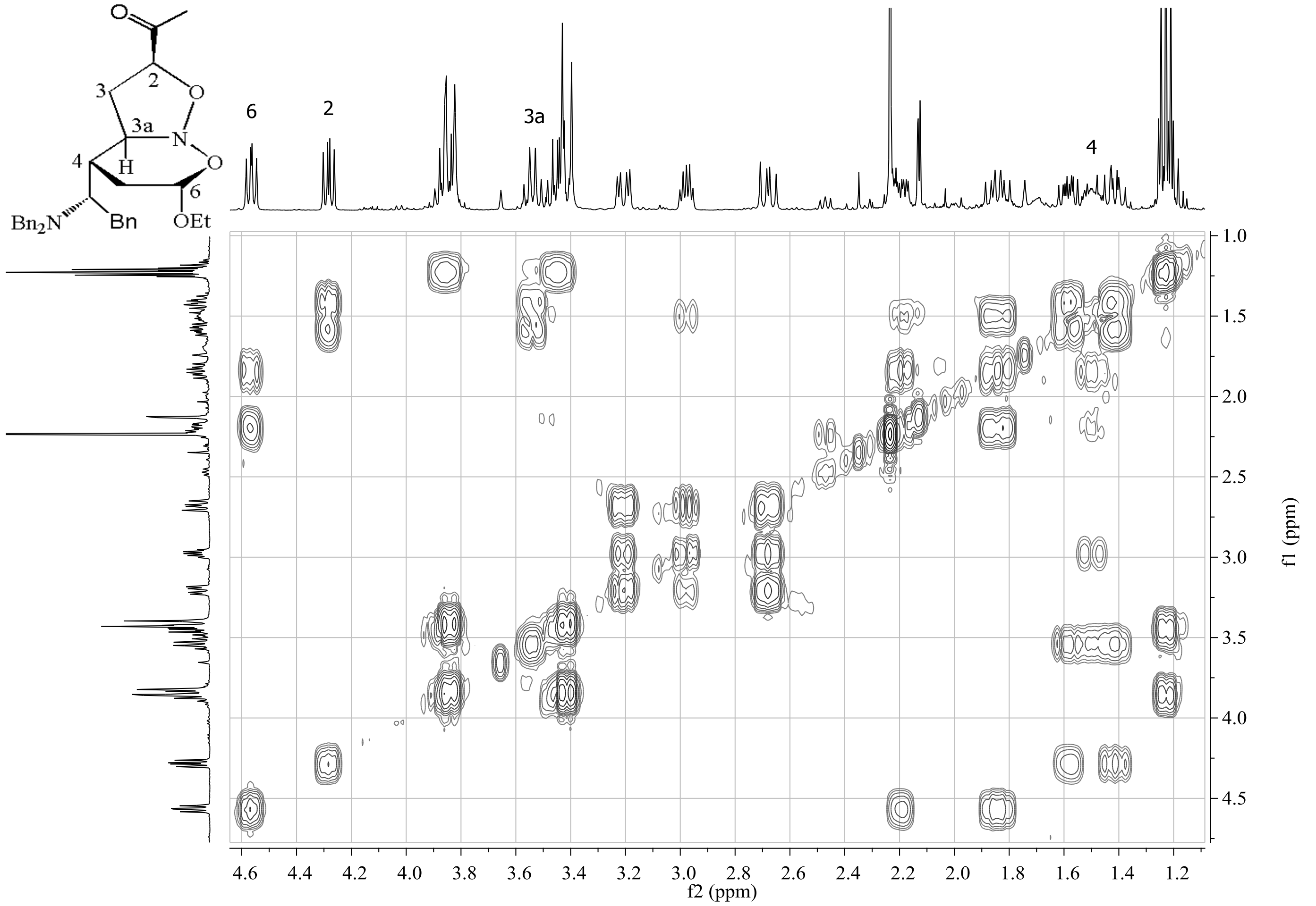


Spectrum 42 – 2D COSY of **12b’**

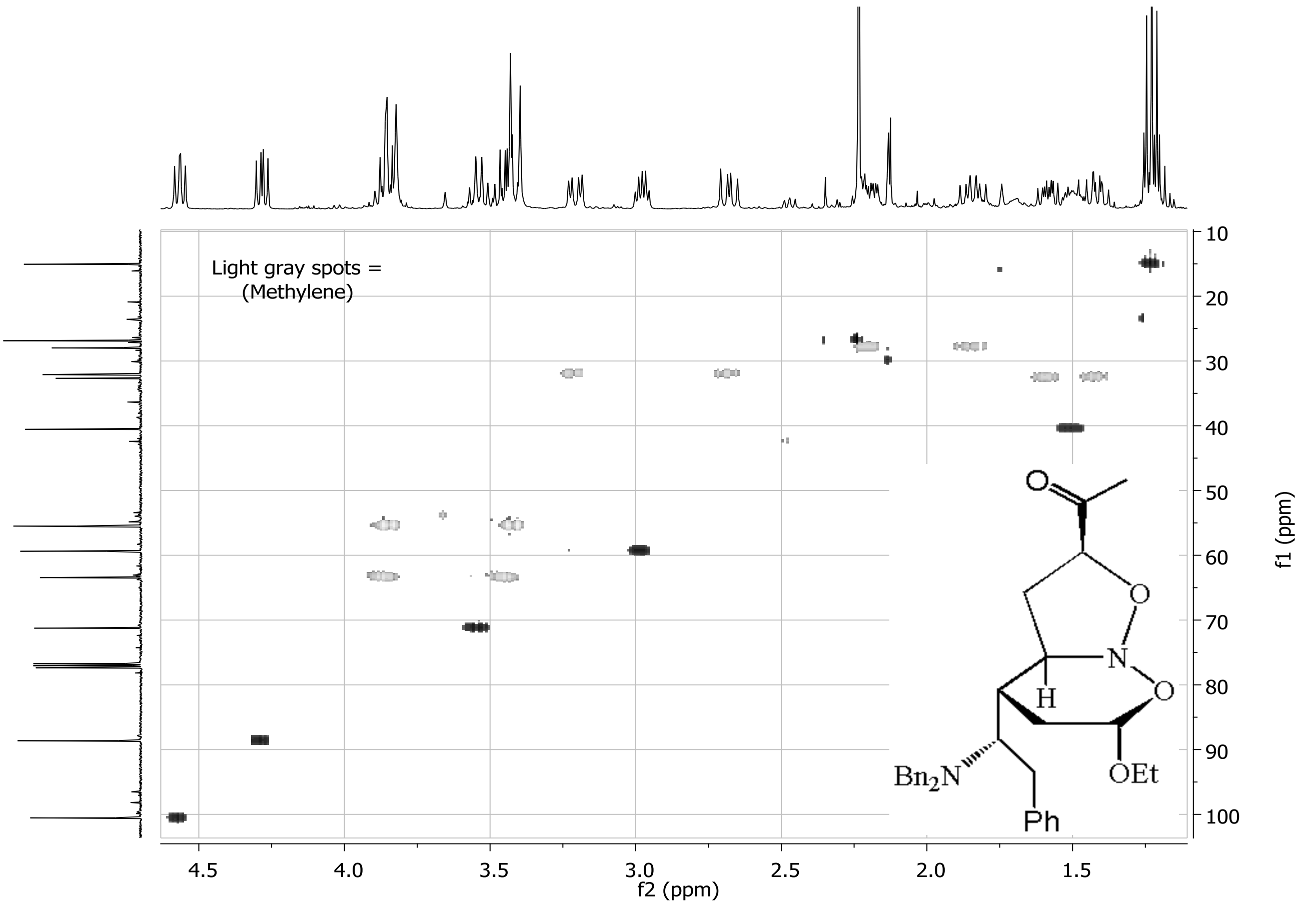


Spectrum 43 – HSQC of **12b’**

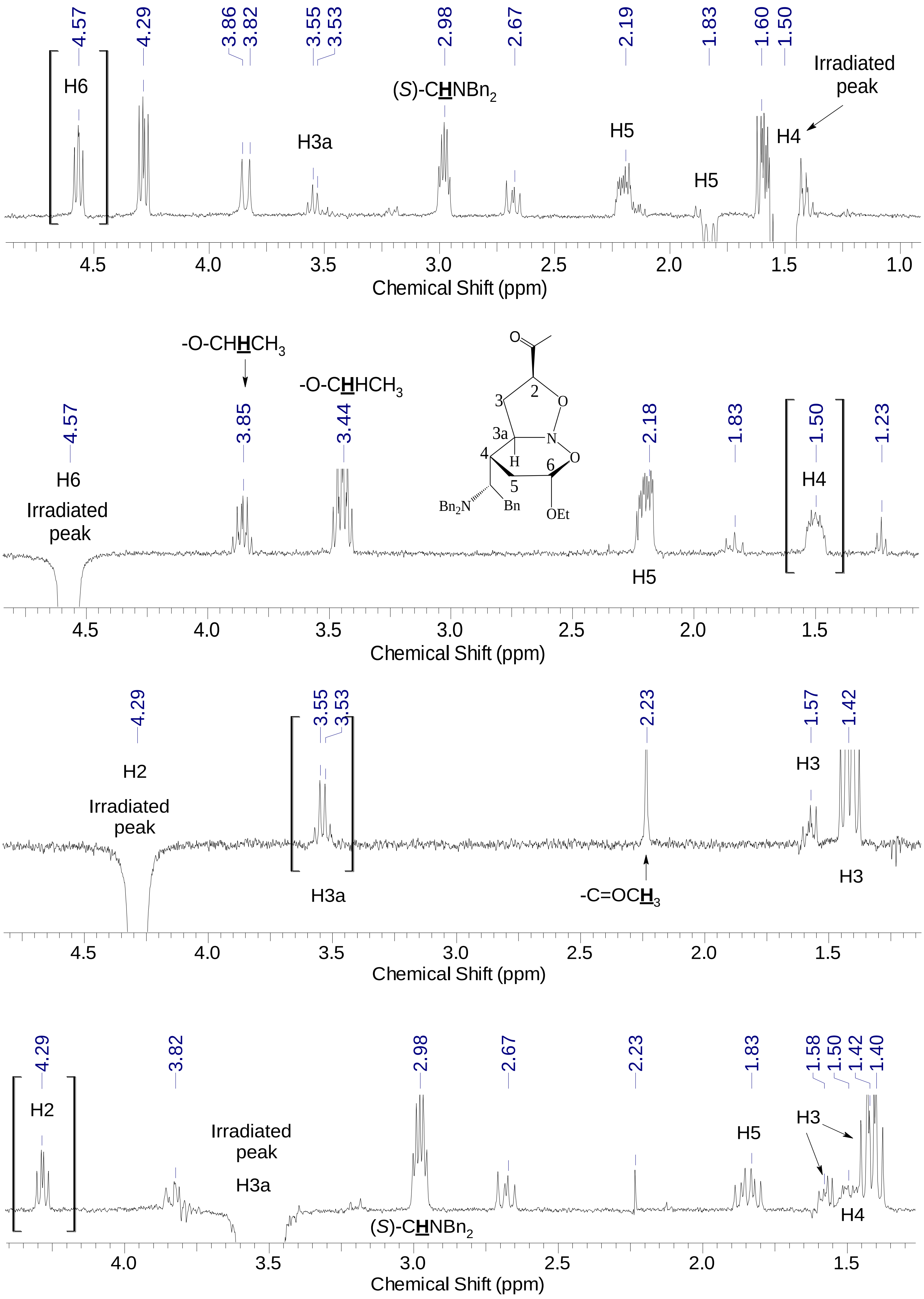


Spectrum 44 – 2D NOESY of **12b’**

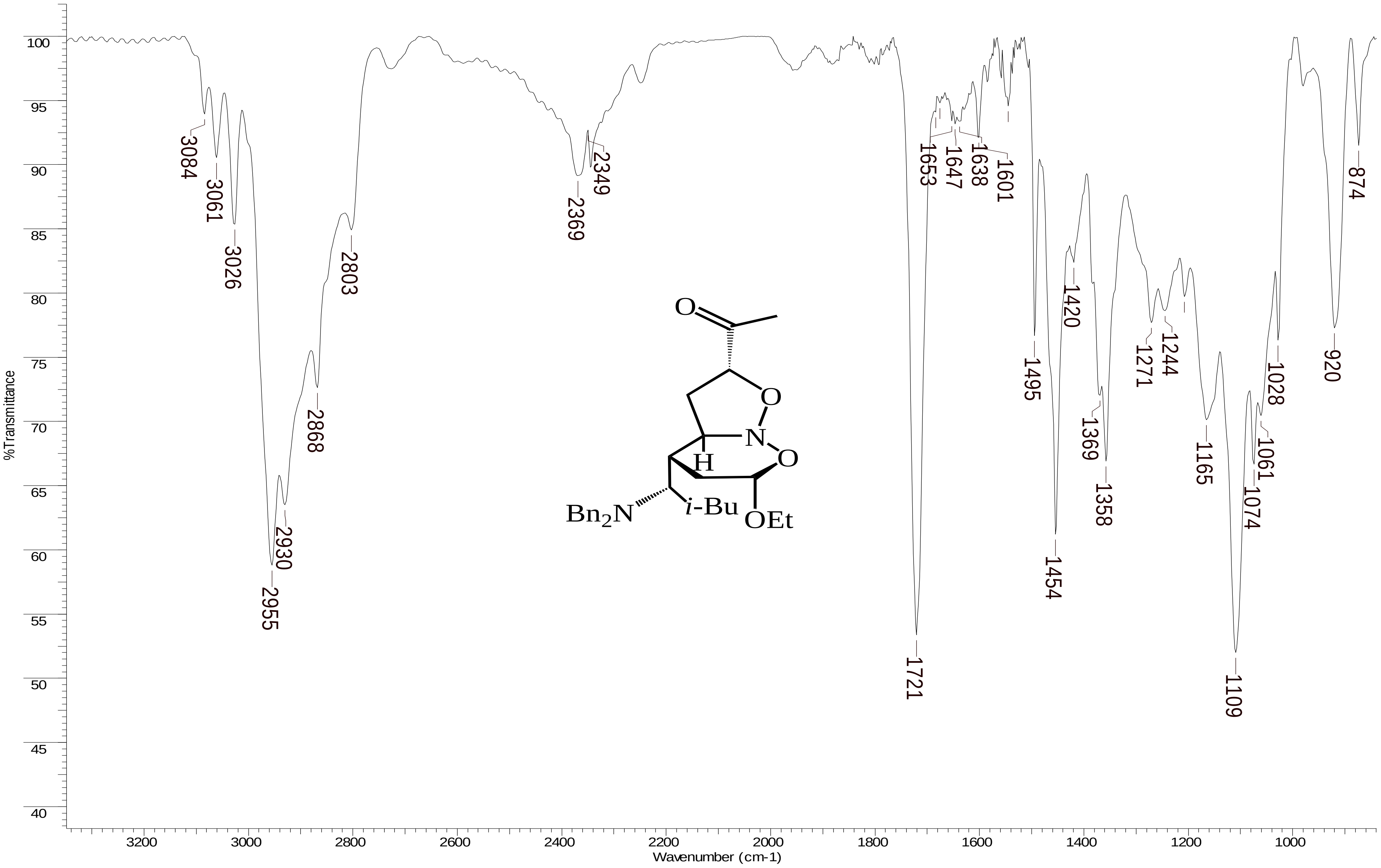


Spectrum 45 – Infrared of **13c**

1H NMR (400 MHz, CHLOROFORM-D) δ ppm 0.92 (d, *J*=5.95 Hz, 3 H); 0.97 (d, *J*=5.95 Hz, 3 H); 1.23 (t, *J*=7.17 Hz, 3 H); 1.27 (m, 1 H); 1.51 (m, 3 H); 1.61 (m, 1 H); 1.78 (m, 1 H); 1.85 (m, 1 H); 2.06 (ddd, *J*=12.84, 6.67, 2.54 Hz, 1 H); 2.14 (s, 3 H); 2.70 (m, 1 H); 3.33 (d, *J*=13.45 Hz, 2 H); 3.50 (m, 1 H); 3.63 (m, 1 H); 3.72 (d, *J*=13.23 Hz, 2 H); 3.94 (m, *J*=14.22, 9.59, 7.06 Hz, 1 H); 4.73 (dd, *J*=7.94, 6.84 Hz, 1H); 4.77 (dd, *J*=10.36, 5.73 Hz, 1H); 7.25 (m, 10 H)

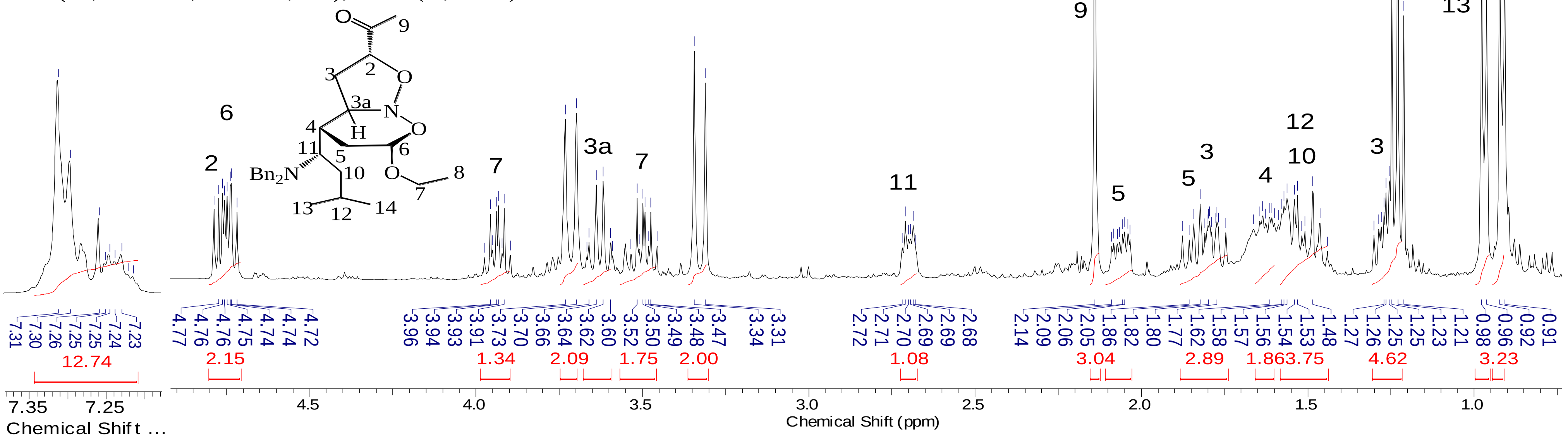


13C NMR (101 MHz, CHLOROFORM-D) δ ppm 15.0 ($CH_3$); 21.8 (CH); 24.0 ($CH_3$); 25.6 ($CH_3$); 26.4 ($CH_3$); 27.7 ($CH_2$); 34.3 ($CH_2$); 35.0 ($CH_2$); 41.2 (CH); 54.6 (CH); 55.5 (2x $CH_2$); 63.6 ($CH_2$); 70.8 (CH); 87.2 (CH); 100.6 (CH); 127.2-129.3 (10x $CH_{Ar}$); 139.8 (2x $C_{ipso}$); 207.1 (C=O)

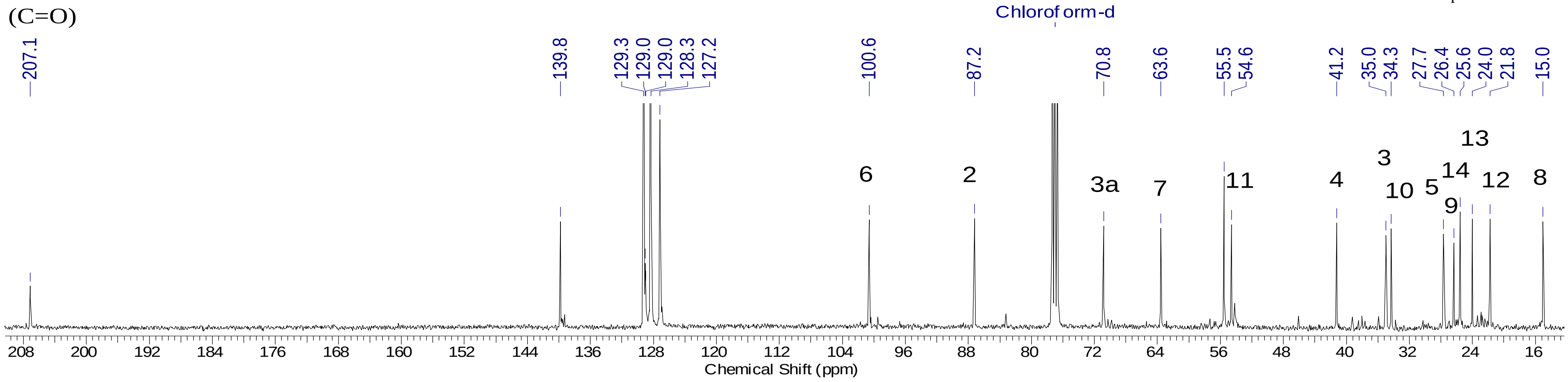


Spectrum 46 - $^1$H and $^{13}$C NMR of **13c**

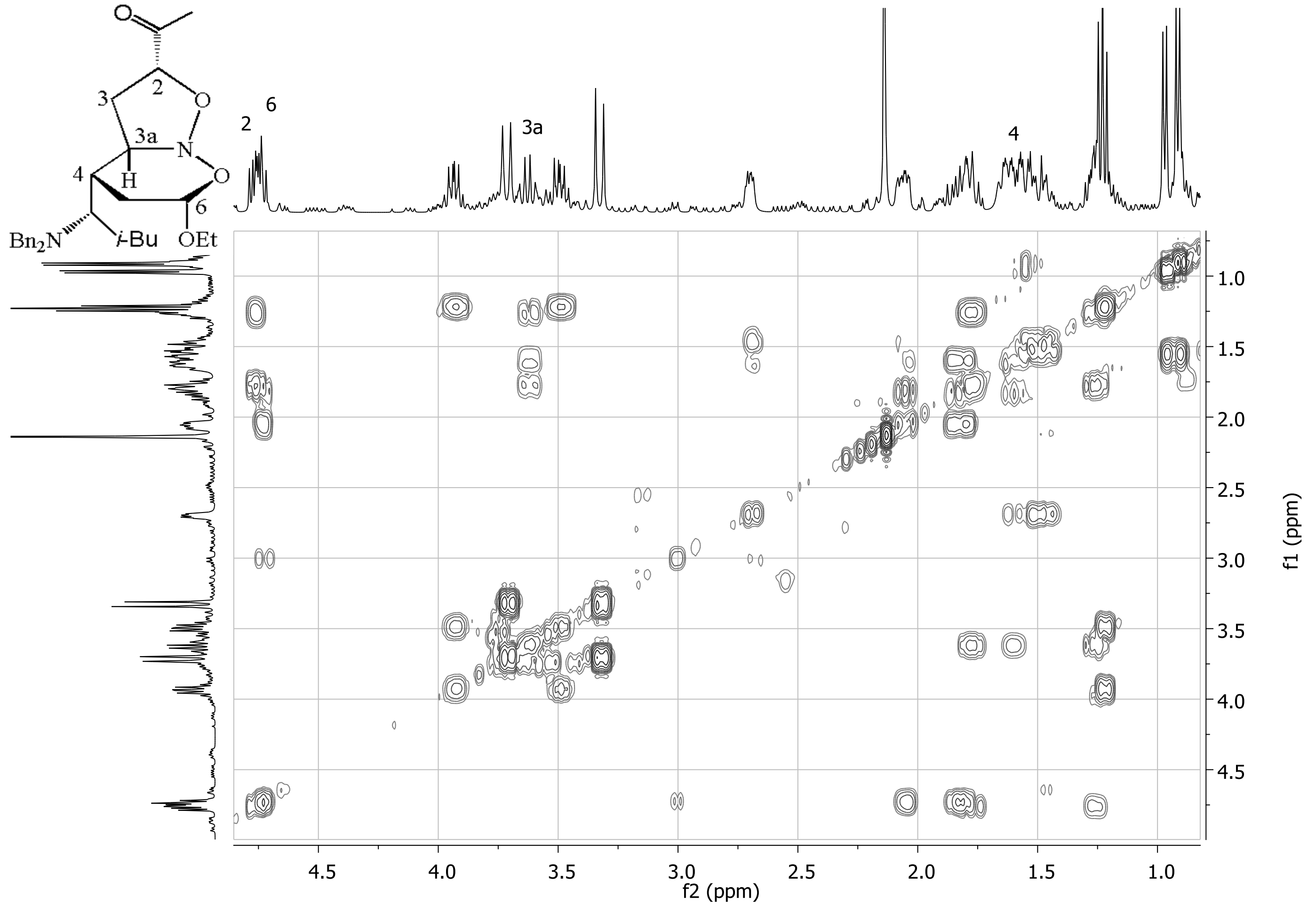


Spectrum 47 – 2D COSY of **13c**

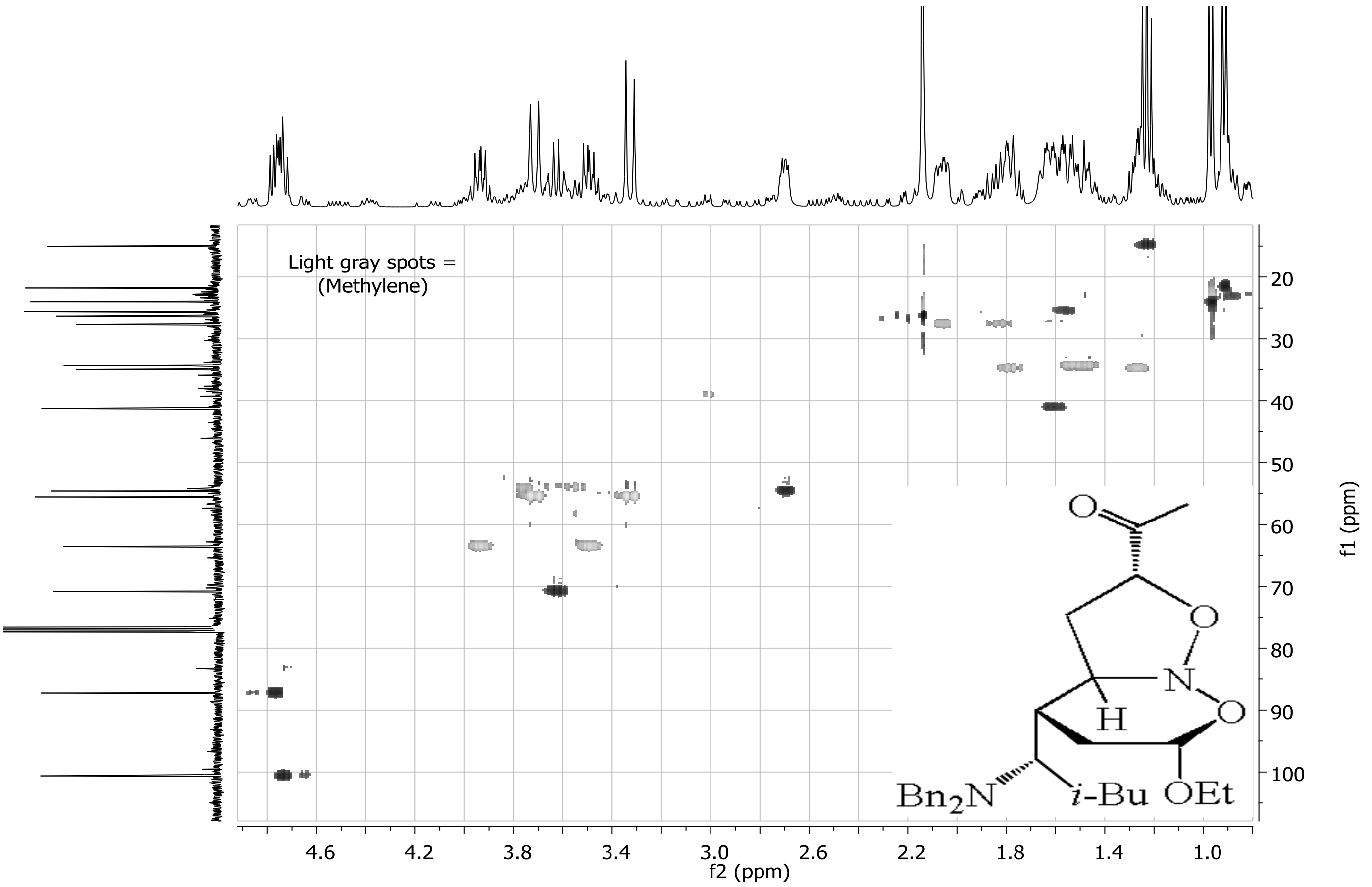


Spectrum 48 – HSQC of **13c**

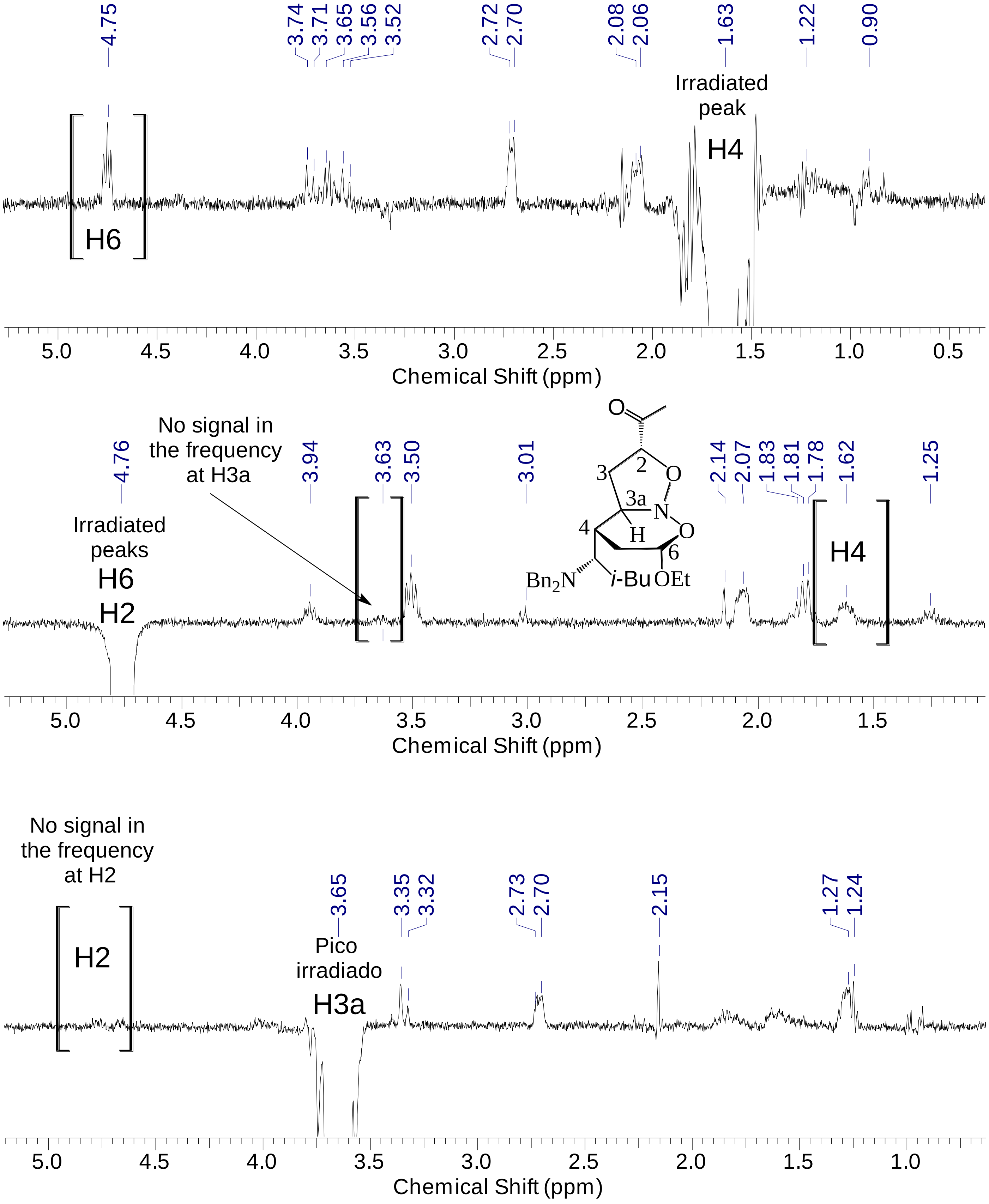


Spectrum 49 – 2D NOESY of **13c**

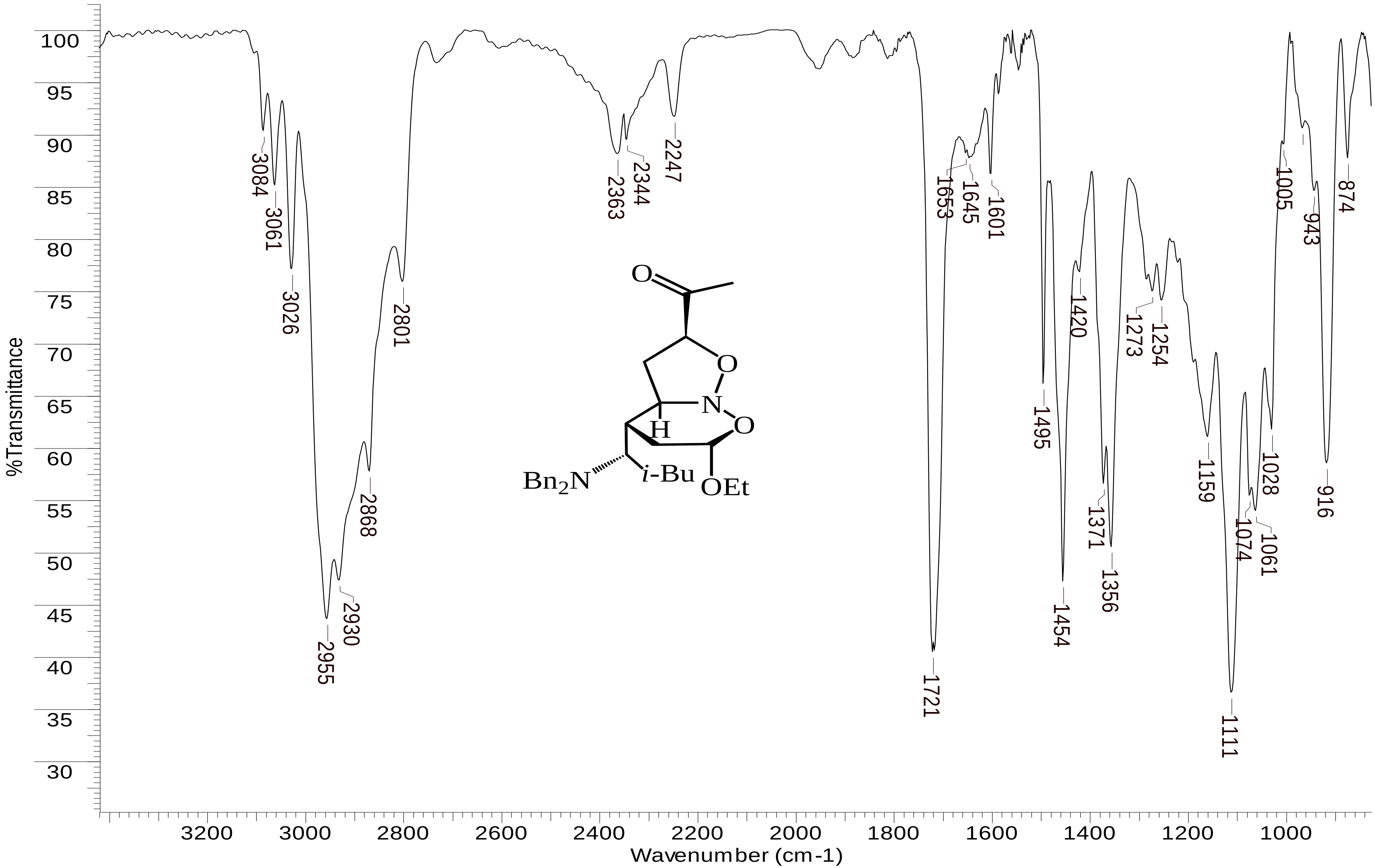


Spectrum 50 – Infrared of **13c’**

1H NMR (400 MHz, CHLOROFORM-D) δ ppm 0.91 (d, *J*=5.95 Hz, 6 H); 1.23 (t, *J*=7.06 Hz, 3 H); 1.40 (m, 1 H); 1.52 (m, 3 H); 1.61 (m, 1 H); 1.79 (m, 1 H); 1.88 (ddd, *J*=12.02, 8.49, 6.17 Hz, 1 H); 2.02 (ddd, *J*=12.79, 6.39, 2.65 Hz, 1 H); 2.31 (s, 3 H); 2.70 (ddd, *J*=8.9, 4.2, 3.8 Hz 1 H); 3.37 (d, *J*=13.45 Hz, 2 H); 3.47 (m, 1 H); 3.61 (q, *J*=8.45 Hz, 1 H); 3.70 (d, *J*=13.23 Hz, 2 H); 3.88 (dq, *J*=9.6, 7.1 Hz, 1 H); 4.37 (dd, *J*=9.70, 5.95 Hz, 1 H); 4.65 (t, *J*=7.72 Hz, 1 H); 7.25 (m, 10 H)

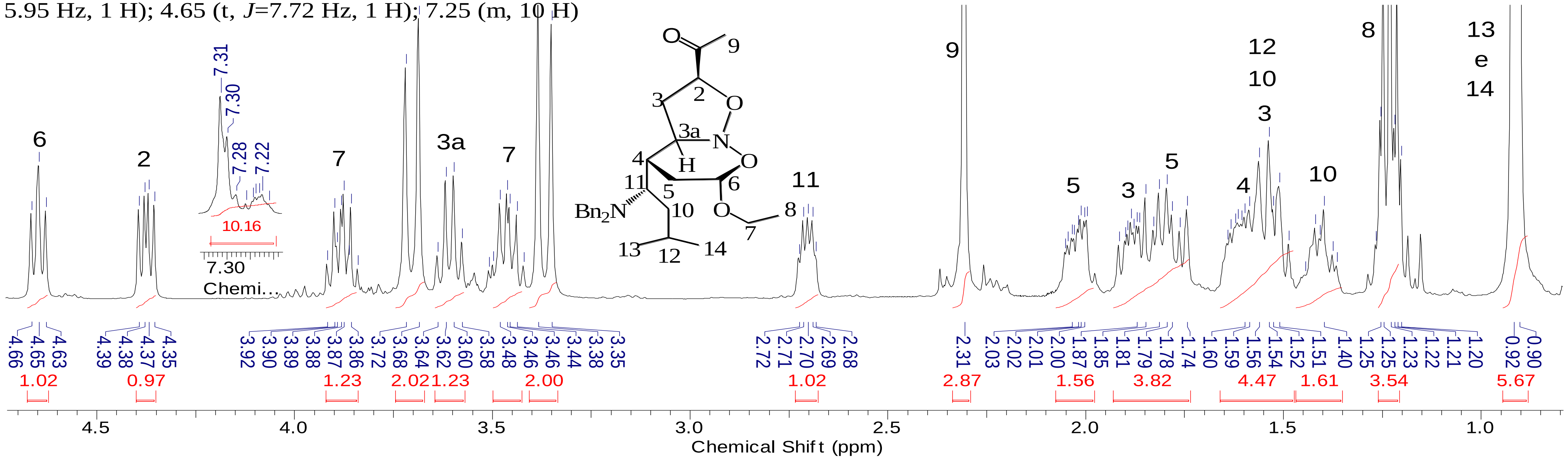


13C NMR (101 MHz, CHLOROFORM-D) δ ppm 15.0 ($CH_3$); 22.0 (CH); 23.7 ($CH_3$); 25.4 ($CH_3$); 26.8 ($CH_3$); 28.0 ($CH_2$); 32.6 ($CH_2$); 34.8 ($CH_2$); 40.9 (CH); 54.9 (CH); 55.3 (2x $CH_2$); 63.4 ($CH_2$); 71.3 (CH); 88.6 (CH); 100.6 (CH); 127.0-129.2 (10x $CH_{Ar}$); 139.8 (2x $C_{ipso}$); 209.2 (C=O)

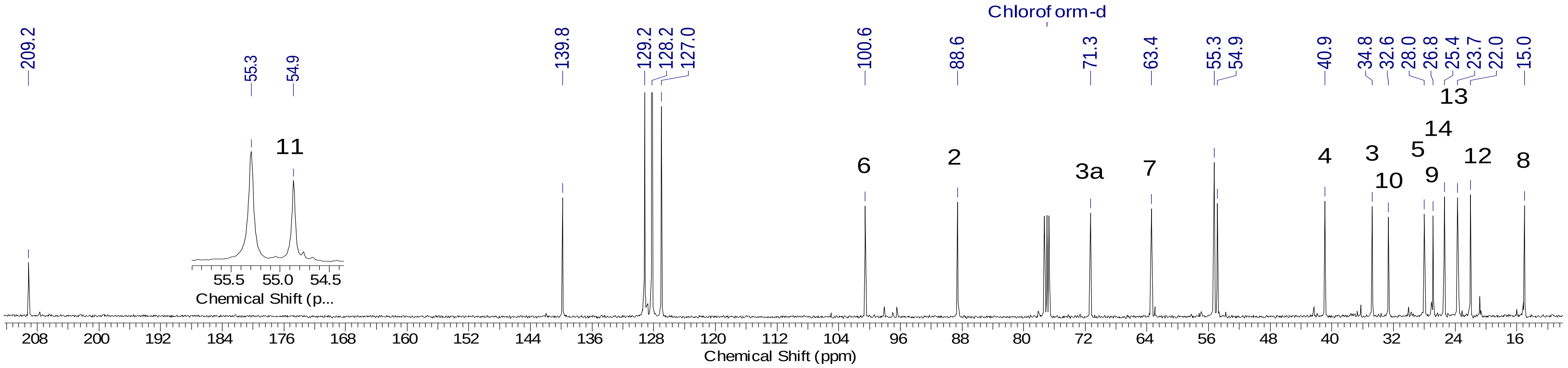


Spectrum 51 - $^1$H and $^{13}$C NMR of **13c'**

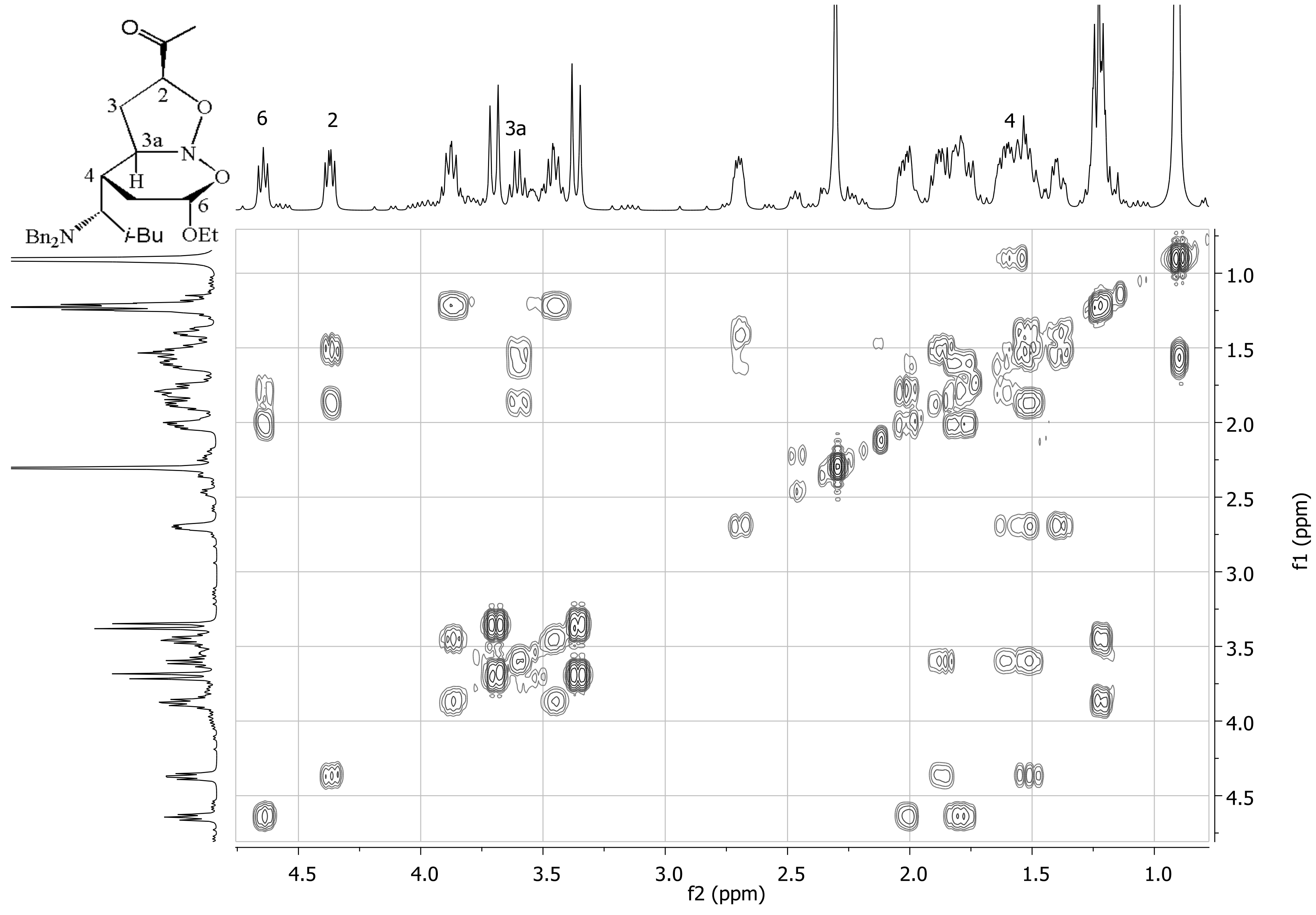


Spectrum 52 – 2D COSY of **13c’**

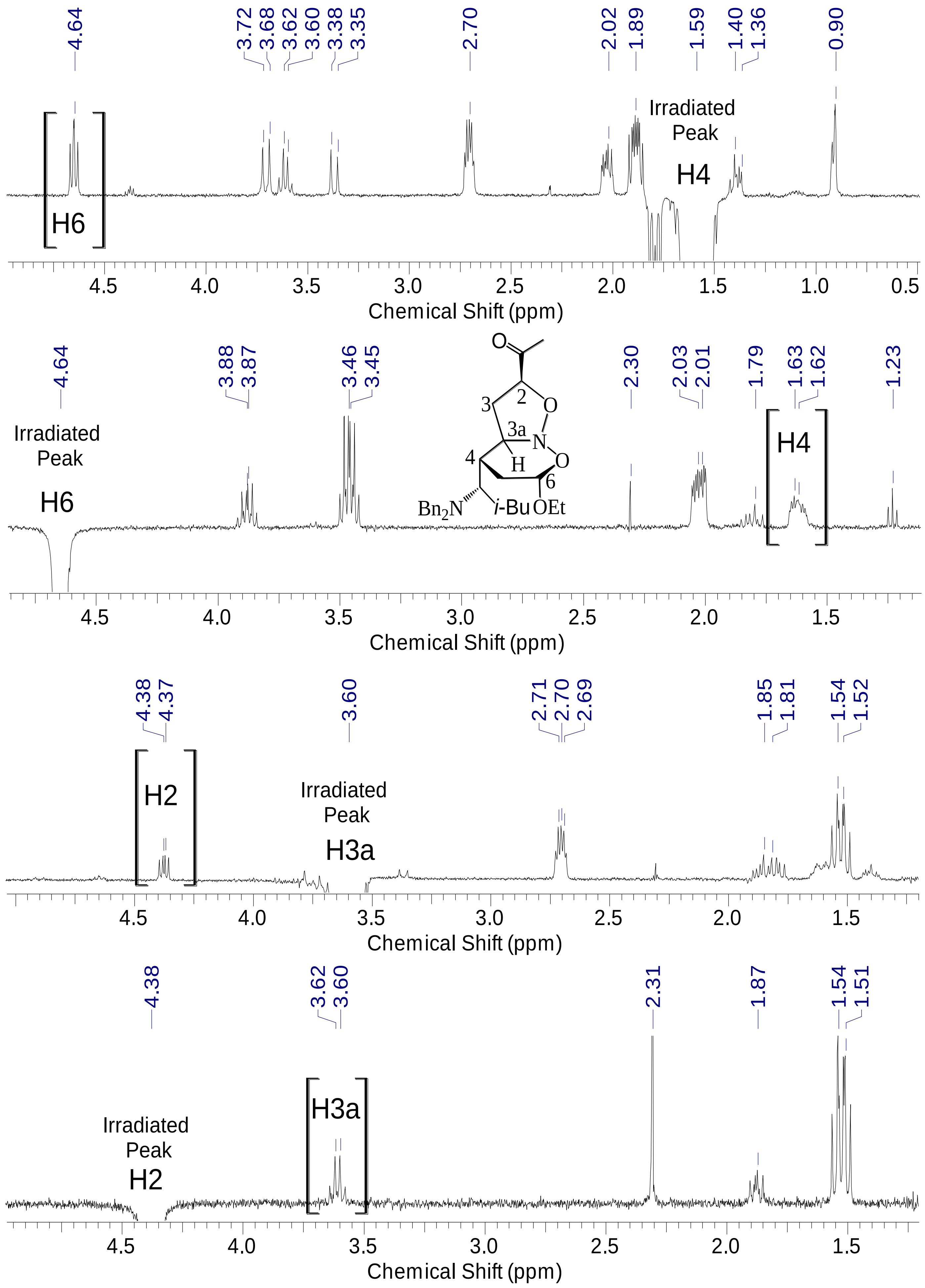


Spectrum 53 – 2D NOESY of **13c’**

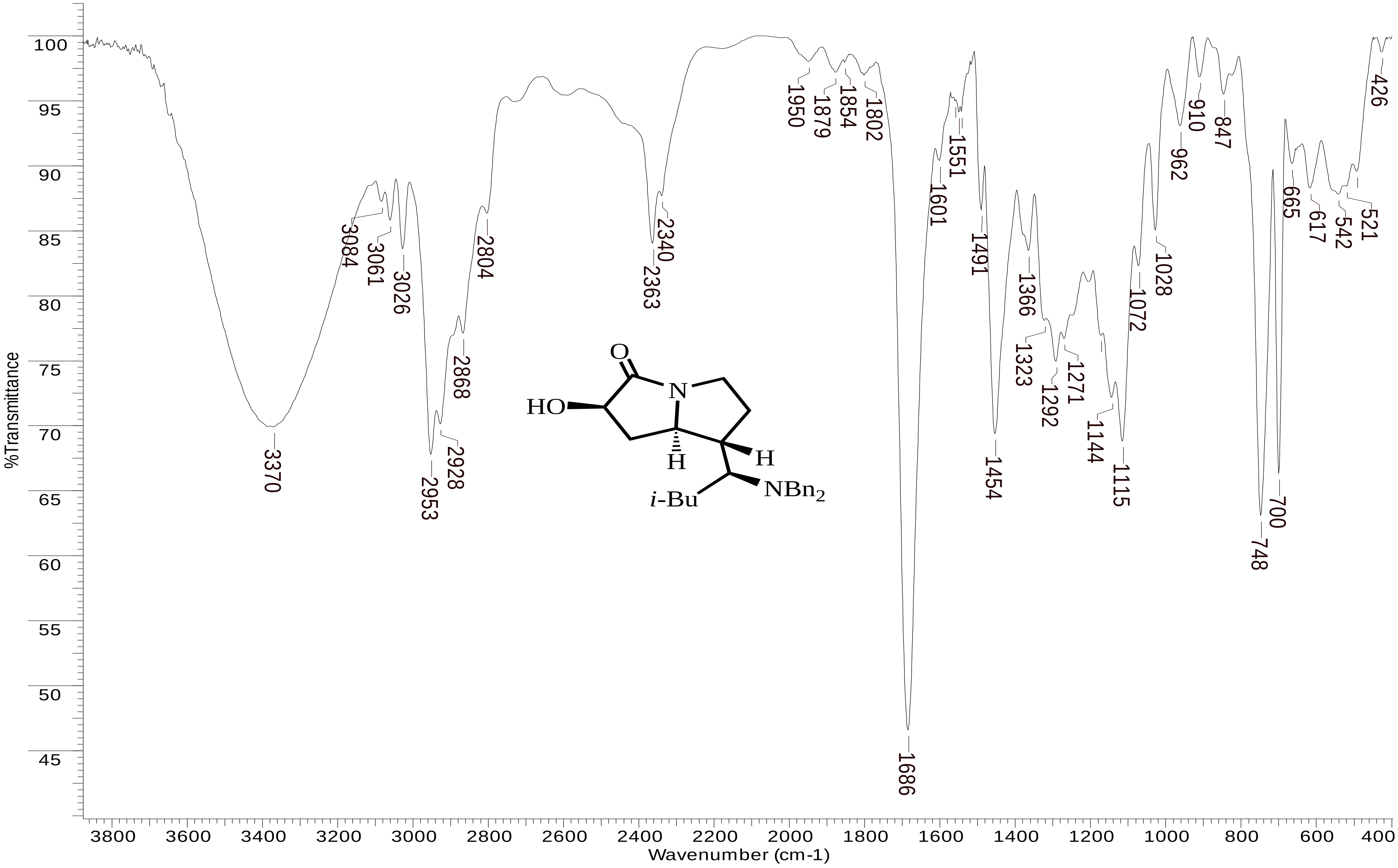


Spectrum 54 – Infrared of **14c**

1H NMR (400 MHz, CHLOROFORM-D) δ ppm 0.86 (d, *J*=6.26 Hz, 3 H); 0.91 (d, *J*=6.26 Hz, 3 H); 1.18 (ddd, *J*=13.30, 7.24, 5.67 Hz, 1 H); 1.52 (ddd, *J*=11.93, 10.56, 8.80 Hz, 1 H); 1.62 (m, 1 H); 1.67 (m, 1 H); 1.80 (m, 1 H); 1.89 (m, 1 H); 2.26 (ddd, *J*=11.74, 7.82, 5.87 Hz, 1 H); 2.43 (m, 1 H); 2.65 (ddd, *J*=6.95, 4.89 Hz, 1 H); 3.15 (dd, *J*=10.96, 9.78 Hz, 1 H); 3.43 (m, 2 H); 3.48 (d, *J*=13.69 Hz, 2 H); 3.70 (d, *J*=13.69 Hz, 2 H); 4.46 (dd, *J*=10.37, 7.63 Hz, 1 H); 7.29 (m, 10 $H_{Ar.}$)

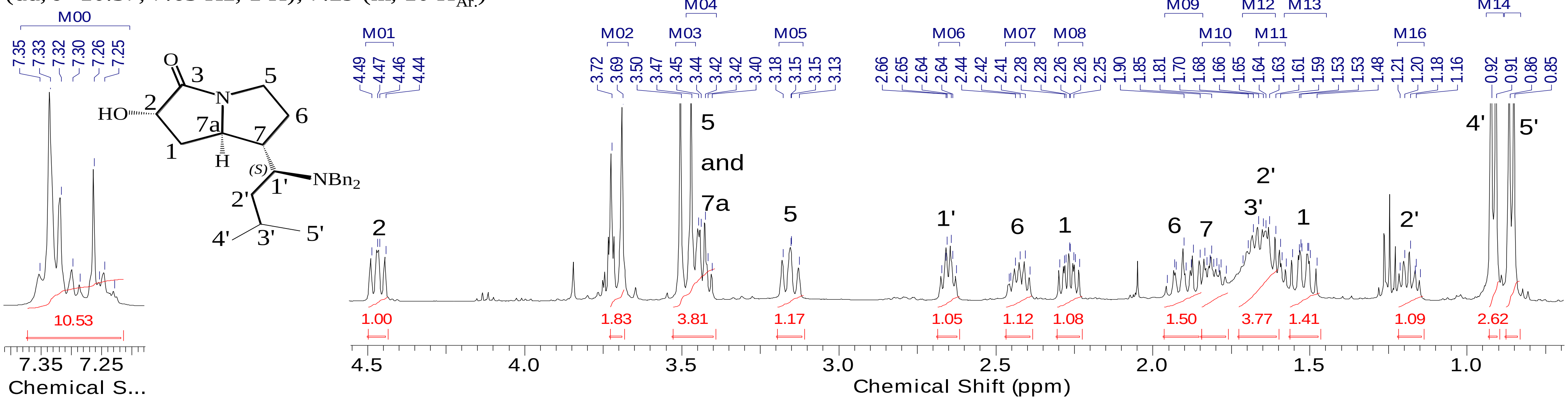


13C NMR (126 MHz, CHLOROFORM-D) δ ppm 22.7 ($CH_2$); 23.2 ($CH_2$); 25.7 (CH); 31.0 ($CH_2$); 37.9 ($CH_2$); 38.1 ($CH_2$); 40.6 ($CH_2$); 49.8 (CH); 54.7 (2 x $CH_2$); 55.9 (CH); 59.3 (CH); 73.1 (CH); 127.1 ($CH_{Ar.}$); 128.2 ($CH_{Ar.}$); 129.0 ($CH_{Ar.}$); 139.9 (2x $C_{ipso}$); 173.9 (C=O)

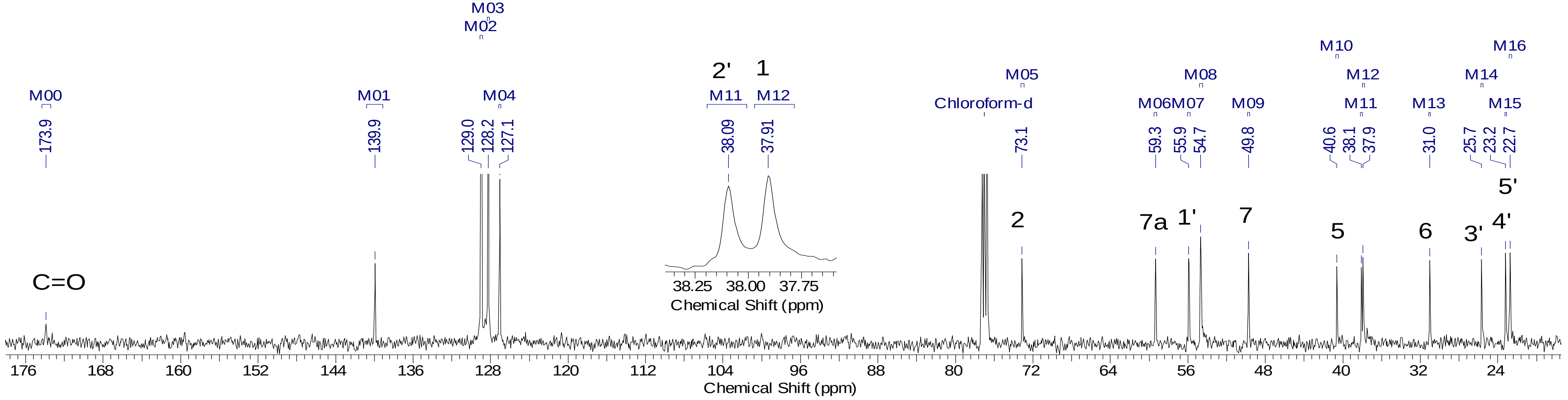


Spectrum 55 - $^{1}$H and $^{13}$C NMR of **14c**

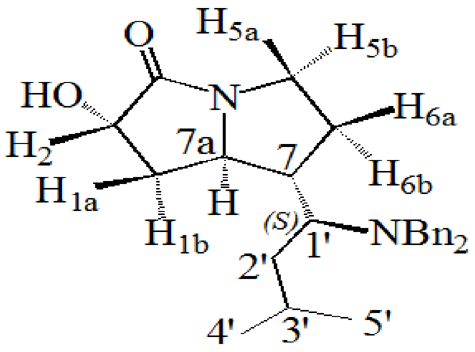


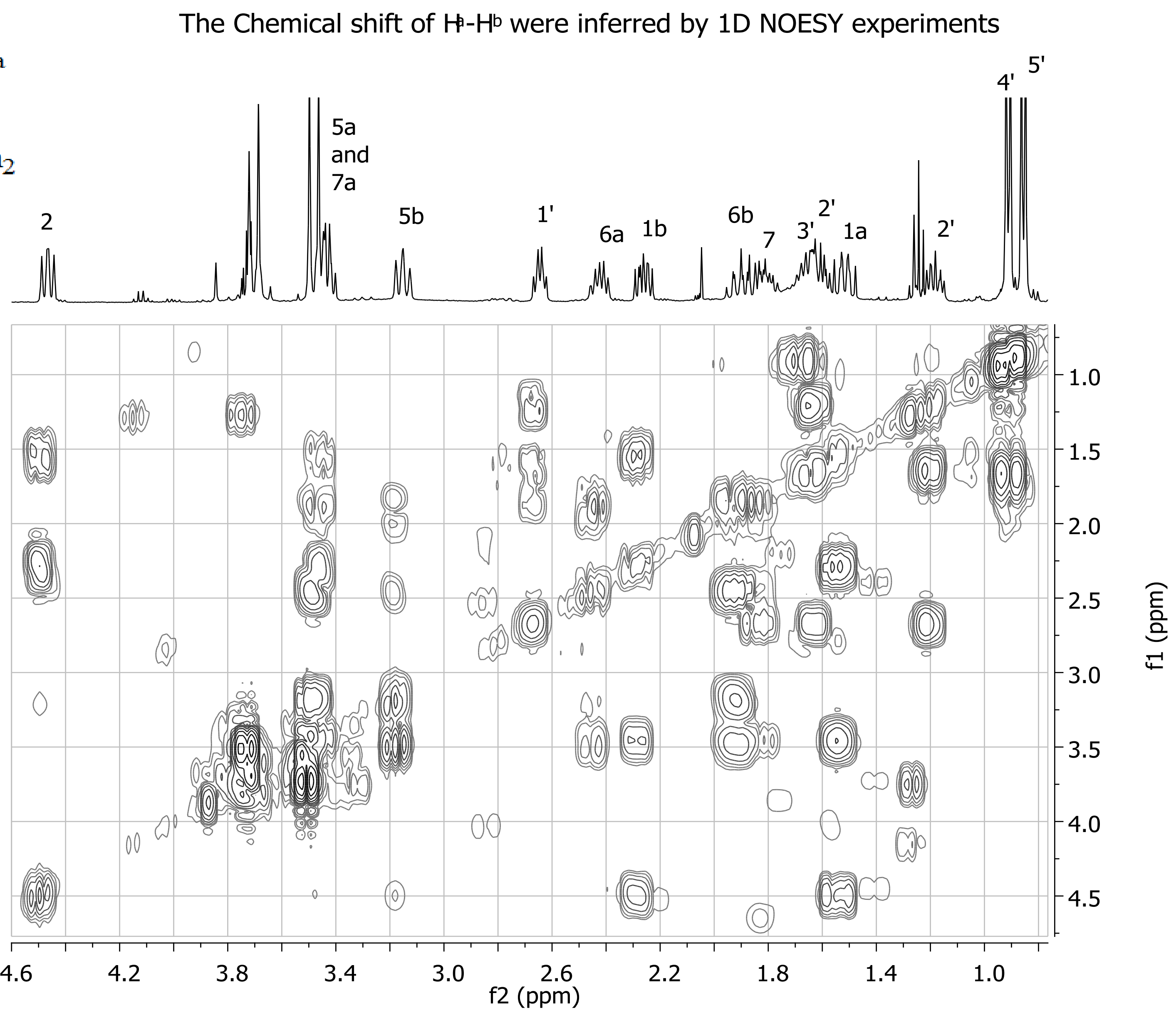


Spectrum 56 – 2D COSY of **14c**

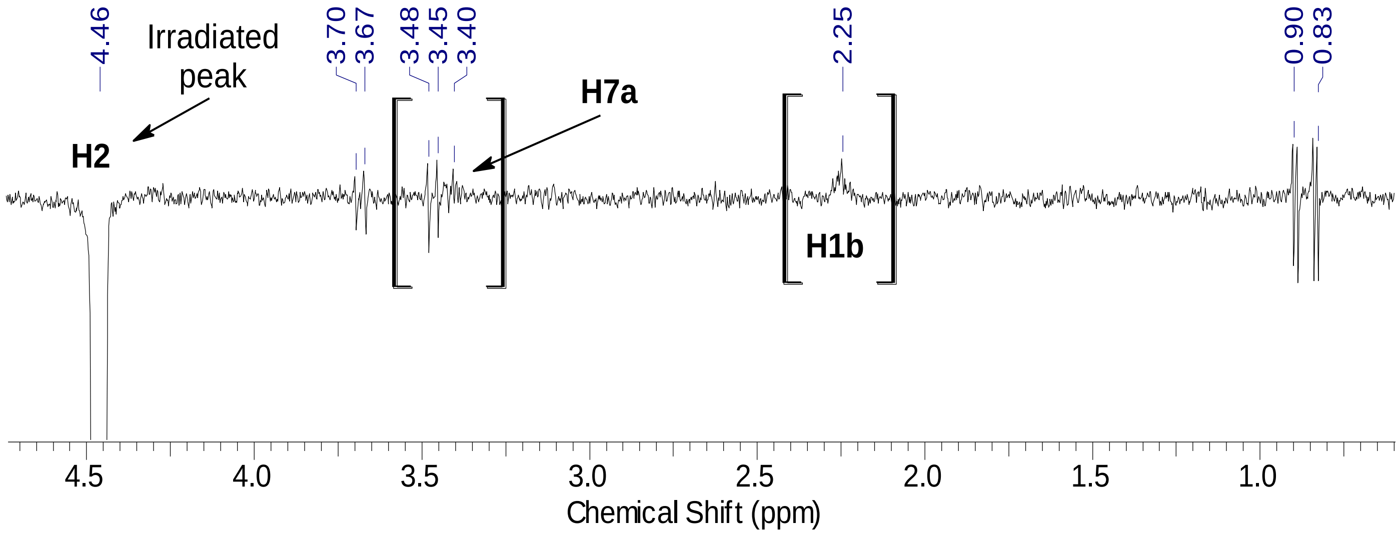
4.46
Irradiated peak
H2
3.70
3.67
3.48
3.45
3.40
H7a
2.25
H1b
0.90
0.83
4.5
4.0
3.5
3.0
2.5
2.0
1.5
1.0
Chemical Shift (ppm)

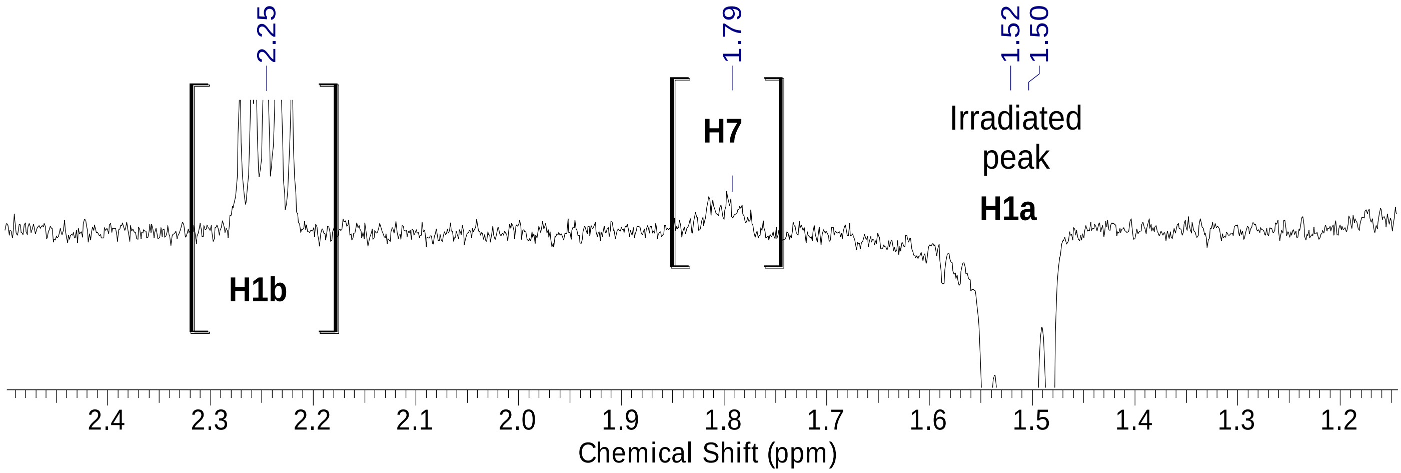
2.25
H1b
1.79
H7
1.52
1.50
Irradiated peak
H1a
2.4
2.3
2.2
2.1
2.0
1.9
1.8
1.7
1.6
1.5
1.4
1.3
1.2
Chemical Shift (ppm)

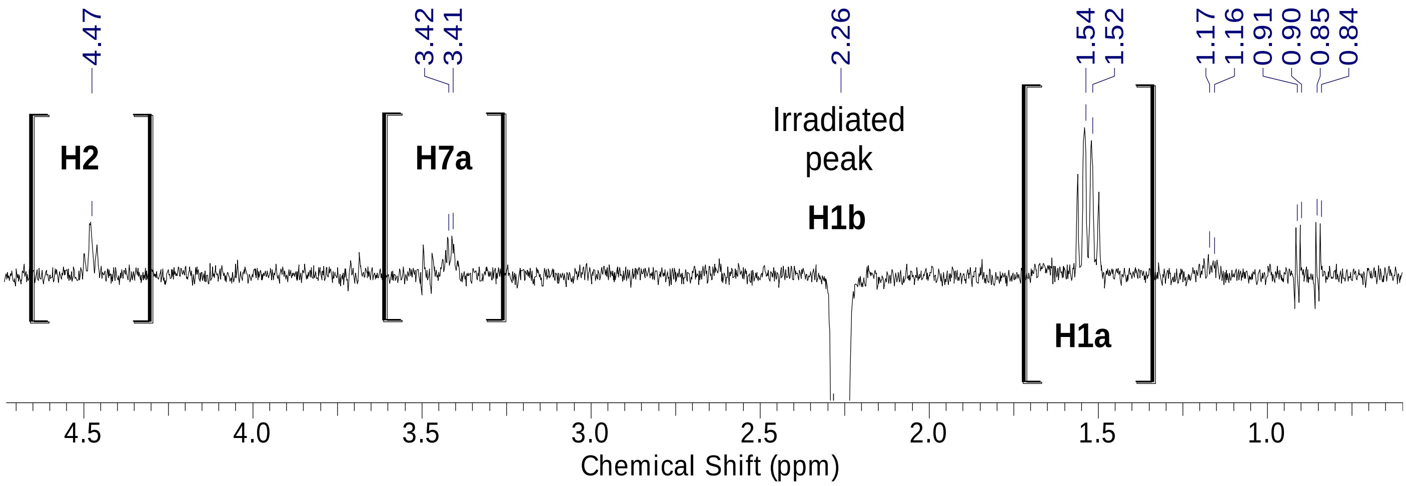
4.47
H2
3.42
3.41
H7a
2.26
Irradiated peak
H1b
1.54
1.52
H1a
1.17
1.16
0.91
0.90
0.85
0.84
4.5
4.0
3.5
3.0
2.5
2.0
1.5
1.0
Chemical Shift (ppm)

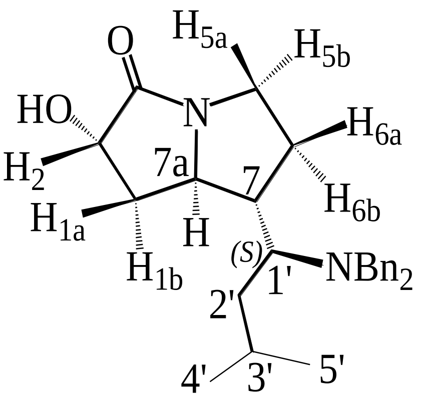
O
HO
N
H5a
H5b
H6a
H6b
H2
7a
7
H1a
H1b
H
(S)
1'
NBn2
2'
3'
4'
5'

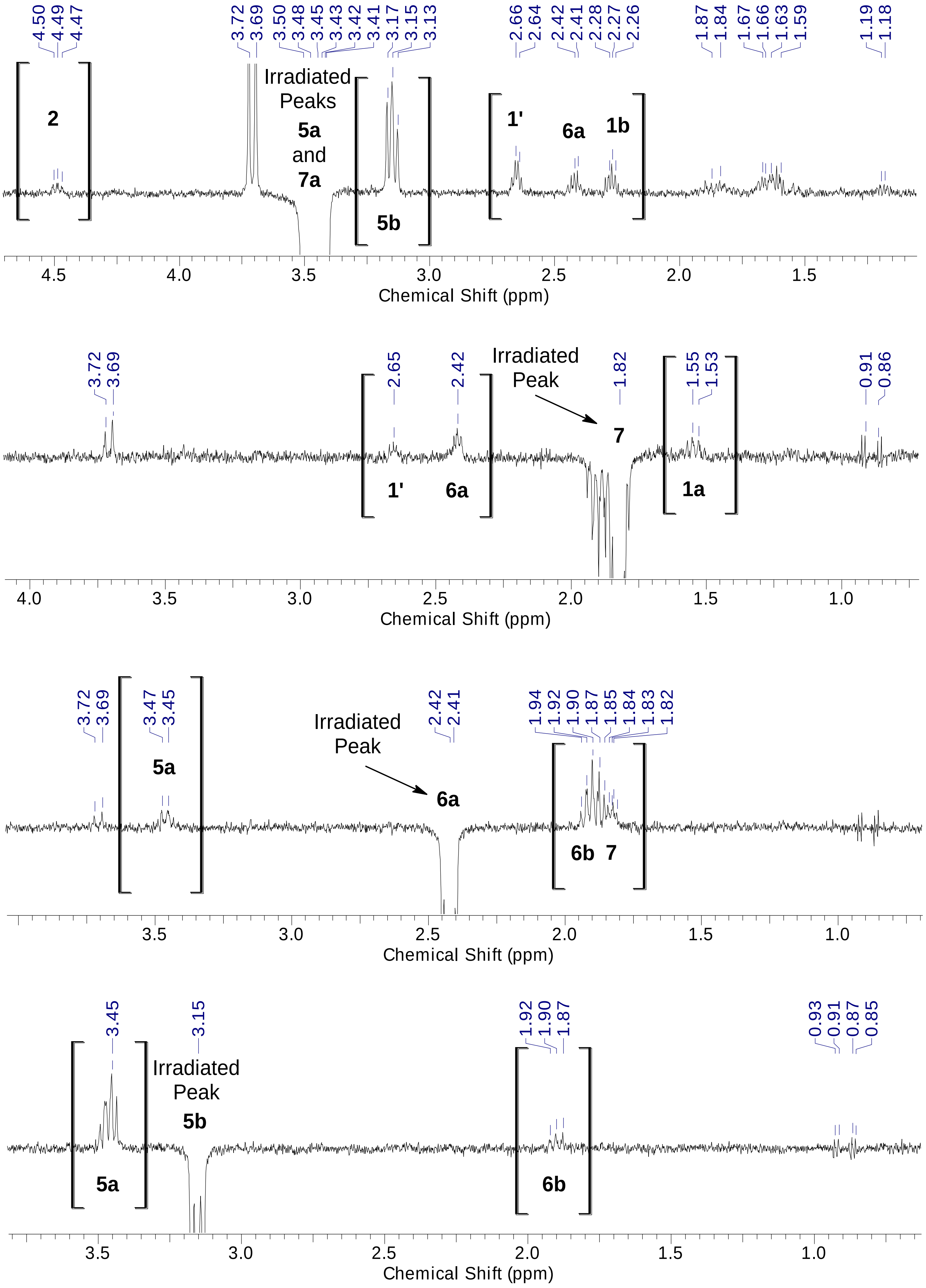


Spectrum 57 – 2D NOESY of **14c**
