# Supplementary material for: Diastereoselective synthesis of nitroso acetals from (S,E)-γ-aminated nitroalkenes via multicomponent [4 + 2]/[3 + 2] cycloadditions promoted by LiCl or LiClO4
Source: Beilstein J Org Chem. 2013 Apr 30;9:838–45. doi: 10.3762/bjoc.9.96 (PMC3678848; doi:10.3762/bjoc.9.96)
Supplement: File 2 — Dataset of X-ray crystallography and extended ORTEP drawing of 11b. [file Beilstein_J_Org_Chem-09-838-s002.pdf]

SUPPORTING INFORMATION

for

Diastereoselective synthesis of nitroso acetals from (*S*,*E*)-γ-aminated nitroalkenes via multicomponent [4 + 2]/[3 + 2] cycloadditions promoted by LiCl or $LiClO_4$

Leandro Lara de Carvalho[1], Robert Alan Burrow[2] and Vera Lúcia Patrocinio Pereira*[1,§]

Address: [1]Núcleo de Pesquisas de Produtos Naturais, Laboratório de Síntese Estereosseletiva de Substâncias Bioativas, Universidade Federal do Rio de Janeiro, 21941-902, Rio de Janeiro, Brazil and [2]Departamento de Química, Laboratório de Materiais Inorgânicos, Universidade Federal de Santa Maria, 97105-900, Santa Maria, Rio Grande do Sul, RS, Brazil

Email: Vera Lúcia Patrocinio Pereira - patrocinio@correio.nppn.ufrj.br

[*]Corresponding author

[§]Tel.: +55 21 2562 6792; Fax: +55 21 2562 6512

Dataset of X-ray crystallography and extended ORTEP drawing of 11b

X-RAY DIFFRACTION EXPERIMENTS OF 11b

The Cambridge Crystallographic Data Centre (CCDC) register number 860534

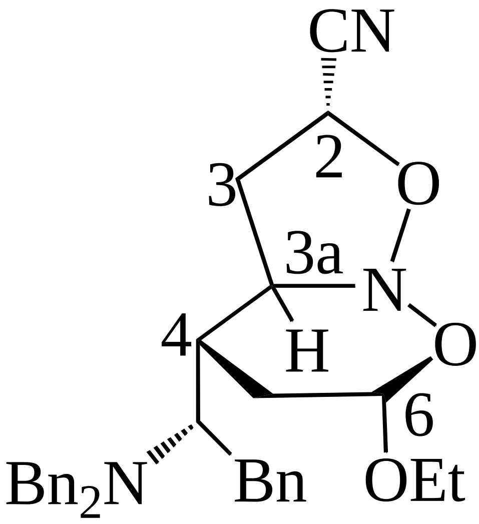

A colorless Block-like specimen of $C_{31}H_{35}N_3O_3$, approximate dimensions 0.180 mm × 0.380 mm × 0.500 mm, was used for the X-ray crystallographic analysis. The X-ray intensity data were measured.

Table 1: Data collection details for 11b.

Axis	dx/ mm	2θ/°	ω/°	φ/°	χ/°	Width/°	Frames	Time/ s	Wave length/ Å	Voltage/ kV	Current/ mA
Omega	40.015	19.50	-358.74	-159.48	-3.90	1.00	103	10.00	0.71073	45	30.0
Omega	40.015	19.50	-344.97	-21.86	-57.17	1.00	115	10.00	0.71073	45	30.0
Omega	40.015	19.50	-350.80	0.59	-24.27	1.00	104	10.00	0.71073	45	30.0
Omega	40.015	19.50	-38.48	75.94	22.60	1.00	69	10.00	0.71073	45	30.0
Omega	40.015	19.50	-358.99	13.87	-7.47	1.00	105	10.00	0.71073	45	30.0
Omega	40.015	19.50	-344.72	-198.97	-54.94	1.00	113	10.00	0.71073	45	30.0
Omega	40.015	19.50	-345.74	0.02	-47.16	1.00	110	10.00	0.71073	45	30.0
Omega	40.015	19.50	-348.97	-233.81	-28.70	1.00	104	10.00	0.71073	45	30.0
Omega	40.015	19.50	-345.40	-52.85	-49.77	1.00	111	10.00	0.71073	45	30.0
Omega	40.015	19.50	-346.88	-177.00	-37.44	1.00	106	10.00	0.71073	45	30.0
Omega	40.015	-19.50	-30.20	110.31	3.93	1.00	92	10.00	0.71073	45	30.0
Omega	40.015	-19.50	-54.06	178.75	54.09	1.00	39	10.00	0.71073	45	30.0
Omega	40.015	-19.50	-55.31	115.62	56.37	1.00	40	10.00	0.71073	45	30.0
Omega	40.015	-19.50	-24.18	96.07	-53.36	1.00	78	10.00	0.71073	45	30.0
Omega	40.015	19.50	-344.55	-221.92	-62.13	1.00	52	10.00	0.71073	45	30.0
Phi	40.015	19.40	0.00	-197.50	0.00	1.00	355	10.00	0.71073	45	30.0
Omega	40.015	-19.50	-51.61	76.30	49.64	1.00	37	10.00	0.71073	45	30.0
Omega	40.015	19.50	-345.56	68.33	-46.18	1.00	109	10.00	0.71073	45	30.0
Omega	40.015	19.50	-345.10	-84.50	-52.24	1.00	112	10.00	0.71073	45	30.0
Omega	40.015	-19.50	-24.17	20.28	-54.92	1.00	79	10.00	0.71073	45	30.0
Omega	40.015	-19.50	-101.69	-67.51	-1.79	1.00	131	10.00	0.71073	45	30.0
Omega	40.015	-19.50	-28.36	-5.01	-27.58	1.00	69	10.00	0.71073	45	30.0
Omega	40.015	19.50	-346.41	31.54	-41.95	1.00	108	10.00	0.71073	45	30.0
Omega	40.015	19.50	-345.45	-103.70	-49.64	1.00	111	10.00	0.71073	45	30.0
Omega	40.015	-19.50	-57.07	-24.60	58.59	1.00	42	10.00	0.71073	45	30.0
Omega	40.015	19.50	-41.08	-53.81	28.70	1.00	69	10.00	0.71073	45	30.0
Omega	40.015	19.50	-358.61	-250.40	-2.10	1.00	102	10.00	0.71073	45	30.0
Omega	40.015	-19.50	-101.76	167.98	-1.64	1.00	131	10.00	0.71073	45	30.0
Omega	40.015	-19.50	-46.65	28.46	40.41	1.00	33	10.00	0.71073	45	30.0

A total of 2829 frames were collected. The total exposure time was 7.86 hours. The frames were integrated with the Bruker SAINT software package using a narrow-frame algorithm. The integration of the data using a monoclinic unit cell yielded a total of 6961 reflections to a maximum θ angle of 28.47° (0.75 Å resolution), of which 6961 were independent (average redundancy 1.000, completeness = 99.4%, Rsig = 5.40%) and 3665 (52.65%) were greater than 2σ(F2). The final cell constants of *a* = 10.2933(5) Å, *b* = 11.2552(6) Å, *c* = 12.6152(6) Å, β = 106.628(2)°, volume = 1400.39(12) $Å^3$, are based upon the refinement of the XYZ-centroids of 9906 reflections above 20 σ(I) with 4.521° < 2θ < 37.94°. The calculated minimum and maximum transmission coefficients (based on crystal size) are 0.9627 and 0.9867.

The crystal was twinned with two main components in a 3:1 intensity ratio. The integration was performed considering both components. During the scaling, only reflections belonging to the more intense component were output for solution and refinement.

The structure was solved and refined using the Bruker SHELXTL Software Package, using the space group P 1 21 1, with *Z* = 2 for the formula unit, C31H35N3O3. The final anisotropic full-matrix least-squares refinement on F2 with 336 variables converged at R1 = 5.01%, for the observed data and wR2 = 11.61% for all data. The goodness-of-fit was 0.977. The largest peak in the final difference electron density synthesis was 0.126 e-/Å3 and the largest hole was -0.098 e-/$Å^3$ with an RMS deviation of 0.027 e-/$Å^3$. On the basis of the final model, the calculated density was 1.180 g/cm^3 and F(000), 532 e-.

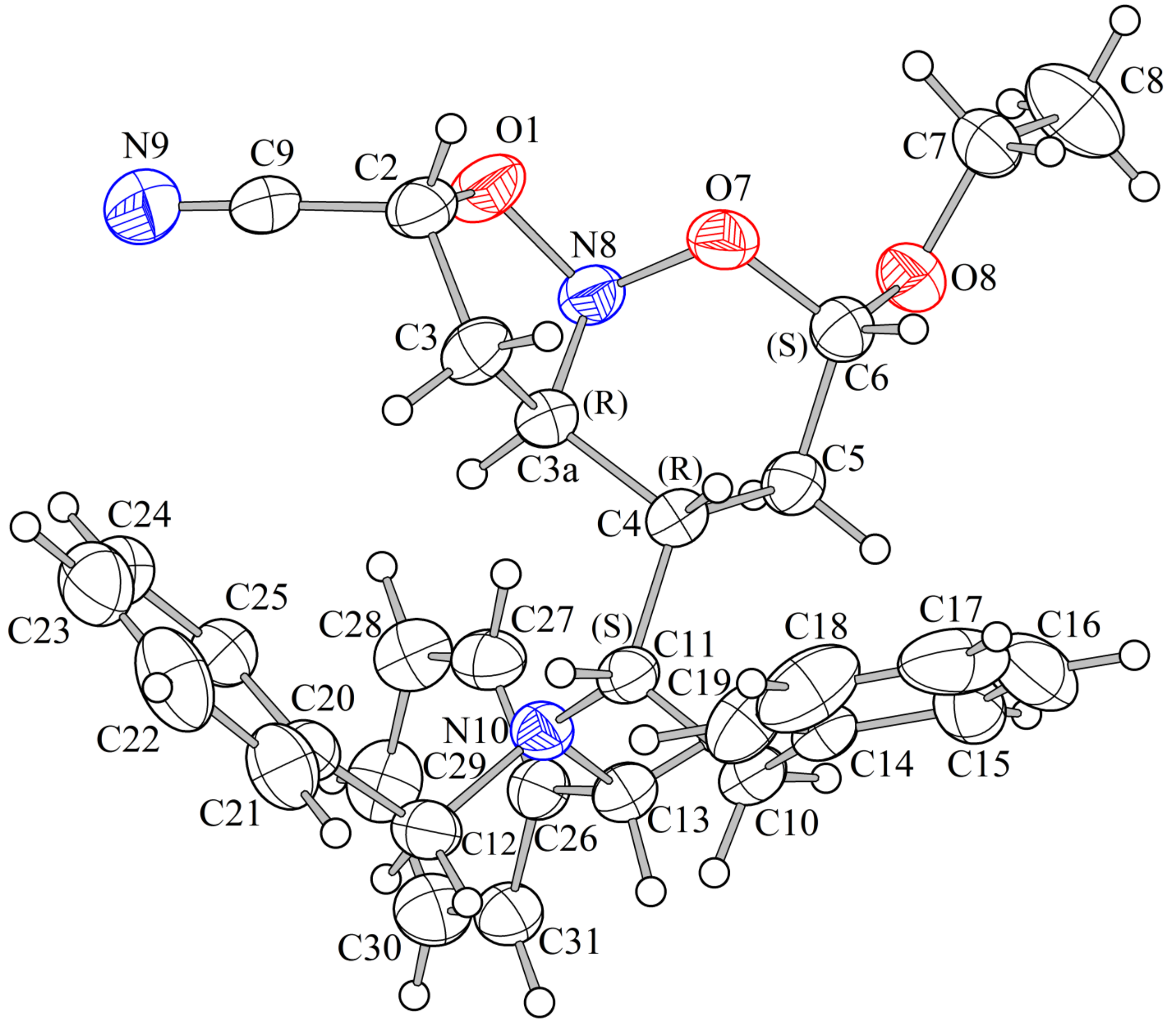
N9
C9
C2
O1
O7
N8
C7
C8
O8
(S)
C6
C3
(R)
C3a
(R)
C5
C4
C24
C23
C25
C28
C27
(S)
C11
C18
C17
C16
C19
C20
N10
C22
C29
C14
C15
C21
C12
C26
C13
C10
C30
C31

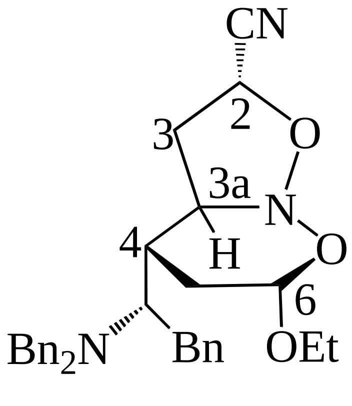
CN
2
3
O
3a
N
4
H
O
6
Bn2N
Bn
OEt

Table 2: Sample and crystal data for 11b.

Identification code	RB_UFRJ_LL271a	
Chemical formula	C31H35N3O3	
Formula weight	497.62	
Temperature	296(2) K	
Wavelength	0.71073 Å	
Crystal size	0.180 x 0.380 x 0.500 mm	
Crystal habit	colorless Block	
Crystal system	monoclinic	
Space group	P 1 21 1	
Unit cell dimensions	a = 10.2933(5) Å	α = 90°
b = 11.2552(6) Å	β = 106.628(2)°	
c = 12.6152(6) Å	γ = 90°	
Volume	1400.39(12) Å3	
Z	2	
Density (calculated)	1.180 Mg/cm3	
Absorption coefficient	0.076 mm-1	
F(000)	532	

Table 3: Data collection and structure refinement for 11b.

Theta range for data collection	2.06 to 28.47°	
Index ranges	-13<=h<=13, -14<=k<=15, -16<=l<=16	
Reflections collected	6961	
Coverage of independent reflections	99.4%	
Max. and min. transmission	0.9867 and 0.9627	
Structure solution technique	direct methods	
Structure solution program	SHELXS-97 (Sheldrick, 2008)	
Refinement method	Full-matrix least-squares on F2	
Refinement program	SHELXL-97 (Sheldrick, 2008)	
Function minimized	Σ w(Fo2 - Fc2)2	
Data / restraints / parameters	6961 / 1 / 336	
Goodness-of-fit on F2	0.977	
Final R indices	3665 data; I>2σ(I)	R1 = 0.0501, wR2 = 0.0992
all data	R1 = 0.1093, wR2 = 0.1161	
Weighting scheme	w=1/[σ2(Fo2)+(0.0544P)2+0.0000P] where P=(Fo2+2Fc2)/3	
Absolute structure parameter	0.0(11)	
Extinction coefficient	0.0164(19)	
Largest diff. peak and hole	0.126 and -0.098 eÅ-3	
R.M.S. deviation from mean	0.027 eÅ-3	

Table 4: Atomic coordinates and equivalent isotropic atomic displacement parameters (Å2) for 11b.
U(eq) is defined as one third of the trace of the orthogonalized Uij tensor.

	x/a	y/b	z/c	U(eq)
O1	0.41159(13)	0.46950(18)	0.33025(14)	0.0829(5)
C2	0.3866(2)	0.4187(2)	0.2218(2)	0.0704(7)
C3	0.51784(19)	0.4282(2)	0.19050(19)	0.0682(6)
C3A	0.60129(17)	0.51134(19)	0.27866(17)	0.0541(5)
C4	0.75518(16)	0.49195(19)	0.31232(16)	0.0524(5)
C5	0.8074(2)	0.5014(2)	0.43787(17)	0.0623(6)
C6	0.7451(2)	0.4021(2)	0.48706(19)	0.0633(6)
C7	0.7222(3)	0.3258(3)	0.6549(2)	0.0821(8)
O7	0.60902(14)	0.37976(14)	0.42203(12)	0.0681(4)
C8	0.7550(4)	0.3571(4)	0.7739(3)	0.1352(13)
N8	0.54976(15)	0.49437(17)	0.37562(14)	0.0613(5)
O8	0.75326(14)	0.42687(15)	0.59655(13)	0.0709(4)
C9	0.2763(2)	0.4862(3)	0.1495(3)	0.0960(9)
N9	0.1922(3)	0.5391(4)	0.0902(3)	0.1558(16)
C10	0.97305(18)	0.5392(2)	0.2618(2)	0.0632(6)
N10	0.79101(15)	0.69538(15)	0.24385(14)	0.0558(4)
C11	0.82155(18)	0.56867(19)	0.24220(17)	0.0540(5)
C12	0.7765(2)	0.7529(2)	0.13663(18)	0.0651(6)
C13	0.8804(2)	0.7642(2)	0.33454(19)	0.0622(6)
C14	0.00280(18)	0.4129(2)	0.24096(19)	0.0610(6)
C15	0.0871(3)	0.3446(3)	0.3197(2)	0.0911(9)
C16	0.1163(4)	0.2282(4)	0.2971(5)	0.1268(14)
C17	0.0607(4)	0.1805(3)	0.1953(5)	0.1272(15)
C18	0.9772(3)	0.2491(4)	0.1153(4)	0.1153(12)
C19	0.9480(2)	0.3630(3)	0.1377(2)	0.0860(8)
C20	0.6492(2)	0.71480(19)	0.05261(19)	0.0610(6)
C21	0.6496(3)	0.6452(2)	0.9633(2)	0.0857(8)
C22	0.5242(5)	0.6058(3)	0.8915(2)	0.1110(11)
C23	0.4056(4)	0.6395(4)	0.9112(3)	0.1104(11)
C24	0.4059(3)	0.7092(4)	0.9966(3)	0.1013(10)
C25	0.5258(2)	0.7462(3)	0.0668(2)	0.0767(7)
C26	0.81170(19)	0.8750(2)	0.35773(16)	0.0559(5)
C27	0.6929(2)	0.8684(2)	0.38730(19)	0.0688(6)
C28	0.6314(2)	0.9692(3)	0.41195(19)	0.0776(7)
C29	0.6871(3)	0.0787(2)	0.4049(2)	0.0806(7)
C30	0.8043(3)	0.0861(2)	0.3750(2)	0.0791(7)
C31	0.8657(2)	0.9851(2)	0.35085(18)	0.0657(6)

Table 5: Bond lengths (Å) for 11b.

O1-N8	1.401(2)	O1-C2	1.436(3)
C2-C9	1.451(4)	C2-C3	1.516(3)
C2-H2	0.98	C3-C3A	1.518(3)
C3-H3A	0.97	C3-H3B	0.97
C3A-N8	1.478(3)	C3A-C4	1.534(2)

C3A-H3AA	0.98	C4-C5	1.524(3)
C4-C11	1.530(3)	C4-H4	0.98
C5-C6	1.508(3)	C5-H5A	0.97
C5-H5B	0.97	C6-O8	1.388(3)
C6-O7	1.428(2)	C6-H6	0.98
C7-O8	1.440(3)	C7-C8	1.484(4)
C7-H7A	0.97	C7-H7B	0.97
O7-N8	1.475(2)	C8-H8A	0.96
C8-H8B	0.96	C8-H8C	0.96
C9-N9	1.136(4)	C10-C14	1.494(3)
C10-C11	1.543(3)	C10-H10A	0.97
C10-H10B	0.97	N10-C11	1.462(3)
N10-C13	1.467(3)	N10-C12	1.468(3)
C11-H11	0.98	C12-C20	1.493(3)
C12-H12A	0.97	C12-H12B	0.97
C13-C26	1.504(3)	C13-H13A	0.97
C13-H13B	0.97	C14-C15	1.355(3)
C14-C19	1.382(3)	C15-C16	1.392(6)
C15-H15	0.93	C16-C17	1.359(6)
C16-H16	0.93	C17-C18	1.362(6)
C17-H17	0.93	C18-C19	1.364(5)
C18-H18	0.93	C19-H19	0.93
C20-C21	1.372(3)	C20-C25	1.378(3)
C21-C22	1.419(4)	C21-H21	0.93
C22-C23	1.367(5)	C22-H22	0.93
C23-C24	1.331(5)	C23-H23	0.93
C24-C25	1.363(4)	C24-H24	0.93
C25-H25	0.93	C26-C31	1.371(3)
C26-C27	1.379(3)	C27-C28	1.377(3)
C27-H27	0.93	C28-C29	1.373(4)
C28-H28	0.93	C29-C30	1.366(4)
C29-H29	0.93	C30-C31	1.375(3)
C30-H30	0.93	C31-H31	0.93

Table 6: Bond angles (°) for 11b.

N8-O1-C2	110.66(15)	O1-C2-C9	106.5(2)
O1-C2-C3	106.87(16)	C9-C2-C3	113.3(2)
O1-C2-H2	110.0	C9-C2-H2	110.0
C3-C2-H2	110.0	C2-C3-C3A	102.25(18)
C2-C3-H3A	111.3	C3A-C3-H3A	111.3
C2-C3-H3B	111.3	C3A-C3-H3B	111.3
H3A-C3-H3B	109.2	N8-C3A-C3	105.35(16)
N8-C3A-C4	109.83(16)	C3-C3A-C4	115.99(17)
N8-C3A-H3AA	108.5	C3-C3A-H3AA	108.5
C4-C3A-H3AA	108.5	C5-C4-C11	118.60(16)
C5-C4-C3A	107.88(16)	C11-C4-C3A	110.87(16)
C5-C4-H4	106.2	C11-C4-H4	106.2

C3A-C4-H4	106.2	C6-C5-C4	108.12(17)
C6-C5-H5A	110.1	C4-C5-H5A	110.1
C6-C5-H5B	110.1	C4-C5-H5B	110.1
H5A-C5-H5B	108.4	O8-C6-O7	112.18(17)
O8-C6-C5	110.41(18)	O7-C6-C5	110.90(17)
O8-C6-H6	107.7	O7-C6-H6	107.7
C5-C6-H6	107.7	O8-C7-C8	108.1(3)
O8-C7-H7A	110.1	C8-C7-H7A	110.1
O8-C7-H7B	110.1	C8-C7-H7B	110.1
H7A-C7-H7B	108.4	C6-O7-N8	107.48(15)
C7-C8-H8A	109.5	C7-C8-H8B	109.5
H8A-C8-H8B	109.5	C7-C8-H8C	109.5
H8A-C8-H8C	109.5	H8B-C8-H8C	109.5
O1-N8-O7	104.10(15)	O1-N8-C3A	104.50(14)
O7-N8-C3A	103.65(15)	C6-O8-C7	113.01(19)
N9-C9-C2	177.6(3)	C14-C10-C11	115.14(16)
C14-C10-H10A	108.5	C11-C10-H10A	108.5
C14-C10-H10B	108.5	C11-C10-H10B	108.5
H10A-C10-H10B	107.5	C11-N10-C13	115.88(15)
C11-N10-C12	112.52(17)	C13-N10-C12	111.18(17)
N10-C11-C4	114.14(17)	N10-C11-C10	114.75(16)
C4-C11-C10	112.57(17)	N10-C11-H11	104.7
C4-C11-H11	104.7	C10-C11-H11	104.7
N10-C12-C20	111.38(18)	N10-C12-H12A	109.4
C20-C12-H12A	109.4	N10-C12-H12B	109.4
C20-C12-H12B	109.4	H12A-C12-H12B	108.0
N10-C13-C26	111.38(15)	N10-C13-H13A	109.4
C26-C13-H13A	109.4	N10-C13-H13B	109.4
C26-C13-H13B	109.4	H13A-C13-H13B	108.0
C15-C14-C19	117.6(3)	C15-C14-C10	122.1(2)
C19-C14-C10	120.3(2)	C14-C15-C16	121.0(3)
C14-C15-H15	119.5	C16-C15-H15	119.5
C17-C16-C15	120.6(4)	C17-C16-H16	119.7
C15-C16-H16	119.7	C16-C17-C18	118.8(4)
C16-C17-H17	120.6	C18-C17-H17	120.6
C17-C18-C19	120.6(4)	C17-C18-H18	119.7
C19-C18-H18	119.7	C18-C19-C14	121.5(3)
C18-C19-H19	119.3	C14-C19-H19	119.3
C21-C20-C25	118.1(2)	C21-C20-C12	122.6(2)
C25-C20-C12	119.2(2)	C20-C21-C22	119.1(3)
C20-C21-H21	120.4	C22-C21-H21	120.4
C23-C22-C21	119.6(3)	C23-C22-H22	120.2
C21-C22-H22	120.2	C24-C23-C22	121.0(3)
C24-C23-H23	119.5	C22-C23-H23	119.5
C23-C24-C25	119.8(3)	C23-C24-H24	120.1
C25-C24-H24	120.1	C24-C25-C20	122.3(3)
C24-C25-H25	118.9	C20-C25-H25	118.9
C31-C26-C27	118.2(2)	C31-C26-C13	121.06(19)

C27-C26-C13	120.7(2)	C28-C27-C26	121.1(2)
C28-C27-H27	119.4	C26-C27-H27	119.4
C29-C28-C27	119.8(2)	C29-C28-H28	120.1
C27-C28-H28	120.1	C30-C29-C28	119.4(2)
C30-C29-H29	120.3	C28-C29-H29	120.3
C29-C30-C31	120.6(2)	C29-C30-H30	119.7
C31-C30-H30	119.7	C26-C31-C30	120.8(2)
C26-C31-H31	119.6	C30-C31-H31	119.6

Table 7: Torsion angles (°) for 11b.

N8-O1-C2-C9	-129.63(19)	N8-O1-C2-C3	-8.2(3)
O1-C2-C3-C3A	-12.1(3)	C9-C2-C3-C3A	104.9(3)
C2-C3-C3A-N8	27.2(2)	C2-C3-C3A-C4	148.85(19)
N8-C3A-C4-C5	-17.9(2)	C3-C3A-C4-C5	-137.2(2)
N8-C3A-C4-C11	-149.30(17)	C3-C3A-C4-C11	91.4(2)
C11-C4-C5-C6	-169.46(17)	C3A-C4-C5-C6	63.5(2)
C4-C5-C6-O8	-161.16(17)	C4-C5-C6-O7	-36.2(2)
O8-C6-O7-N8	90.41(19)	C5-C6-O7-N8	-33.6(2)
C2-O1-N8-O7	-82.7(2)	C2-O1-N8-C3A	25.7(2)
C6-O7-N8-O1	-169.83(15)	C6-O7-N8-C3A	81.11(17)
C3-C3A-N8-O1	-32.9(2)	C4-C3A-N8-O1	-158.44(17)
C3-C3A-N8-O7	75.92(17)	C4-C3A-N8-O7	-49.67(18)
O7-C6-O8-C7	68.1(2)	C5-C6-O8-C7	-167.63(17)
C8-C7-O8-C6	171.3(2)	O1-C2-C9-N9	138.(9)
C3-C2-C9-N9	21.(10)	C13-N10-C11-C4	86.7(2)
C12-N10-C11-C4	-143.82(16)	C13-N10-C11-C10	-45.4(3)
C12-N10-C11-C10	84.1(2)	C5-C4-C11-N10	-70.7(2)
C3A-C4-C11-N10	54.9(2)	C5-C4-C11-C10	62.4(2)
C3A-C4-C11-C10	-171.97(17)	C14-C10-C11-N10	-169.81(19)
C14-C10-C11-C4	57.4(2)	C11-N10-C12-C20	70.9(2)
C13-N10-C12-C20	-157.24(19)	C11-N10-C13-C26	-156.48(18)
C12-N10-C13-C26	73.4(2)	C11-C10-C14-C15	-123.1(2)
C11-C10-C14-C19	59.3(3)	C19-C14-C15-C16	-0.5(4)
C10-C14-C15-C16	-178.1(2)	C14-C15-C16-C17	0.0(5)
C15-C16-C17-C18	0.8(5)	C16-C17-C18-C19	-1.1(5)
C17-C18-C19-C14	0.6(5)	C15-C14-C19-C18	0.2(4)
C10-C14-C19-C18	177.8(2)	N10-C12-C20-C21	-111.3(2)
N10-C12-C20-C25	66.0(3)	C25-C20-C21-C22	-1.8(4)
C12-C20-C21-C22	175.6(2)	C20-C21-C22-C23	1.2(4)
C21-C22-C23-C24	0.3(5)	C22-C23-C24-C25	-1.1(5)
C23-C24-C25-C20	0.6(4)	C21-C20-C25-C24	0.9(4)
C12-C20-C25-C24	-176.5(3)	N10-C13-C26-C31	-121.3(2)
N10-C13-C26-C27	59.1(3)	C31-C26-C27-C28	-1.5(3)
C13-C26-C27-C28	178.0(2)	C26-C27-C28-C29	1.4(3)
C27-C28-C29-C30	-1.0(4)	C28-C29-C30-C31	0.7(4)
C27-C26-C31-C30	1.3(3)	C13-C26-C31-C30	-178.3(2)
C29-C30-C31-C26		-0.9(3)	

Table 8: Anisotropic atomic displacement parameters (Å2) for 11b. The anisotropic atomic displacement factor exponent takes the form: -2π2[h2 a*2 U11 + ... + 2 h k a* b* U12]

	U11	U22	U33	U23	U13	U12
O1	0.0511(8)	0.1244(15)	0.0777(11)	-0.0079(11)	0.0257(7)	-0.0077(9)
C2	0.0577(12)	0.0823(17)	0.0690(16)	-0.0013(13)	0.0145(11)	-0.0185(12)
C3	0.0561(11)	0.0799(16)	0.0671(14)	-0.0135(13)	0.0151(10)	-0.0085(11)
C3A	0.0499(10)	0.0557(13)	0.0586(13)	-0.0015(10)	0.0184(9)	-0.0019(9)
C4	0.0468(9)	0.0543(13)	0.0566(12)	-0.0051(10)	0.0155(8)	-0.0032(10)
C5	0.0558(10)	0.0715(15)	0.0566(13)	0.0001(12)	0.0111(9)	-0.0070(11)
C6	0.0606(12)	0.0699(17)	0.0595(15)	0.0026(11)	0.0172(10)	0.0013(11)
C7	0.0811(15)	0.0909(19)	0.0788(19)	0.0276(16)	0.0304(13)	0.0089(14)
O7	0.0693(9)	0.0648(10)	0.0705(10)	0.0040(8)	0.0203(7)	-0.0158(8)
C8	0.194(4)	0.144(3)	0.082(2)	0.027(2)	0.062(2)	0.003(3)
N8	0.0515(9)	0.0716(13)	0.0635(11)	-0.0006(10)	0.0206(8)	-0.0039(9)
O8	0.0799(10)	0.0746(10)	0.0602(10)	0.0069(8)	0.0231(7)	0.0011(8)
C9	0.0544(13)	0.137(3)	0.100(2)	0.044(2)	0.0264(13)	-0.0089(16)
N9	0.0735(15)	0.241(4)	0.157(3)	0.104(3)	0.0390(16)	0.0137(19)
C10	0.0476(11)	0.0730(16)	0.0698(16)	-0.0032(11)	0.0181(10)	-0.0073(10)
N10	0.0567(9)	0.0544(12)	0.0536(11)	-0.0004(9)	0.0117(8)	-0.0099(8)
C11	0.0478(10)	0.0606(14)	0.0535(13)	-0.0031(10)	0.0141(9)	-0.0076(9)
C12	0.0688(13)	0.0660(14)	0.0629(14)	0.0028(12)	0.0228(11)	-0.0115(11)
C13	0.0525(11)	0.0645(15)	0.0662(15)	0.0006(12)	0.0117(10)	-0.0109(11)
C14	0.0434(10)	0.0712(17)	0.0724(16)	0.0033(13)	0.0233(10)	-0.0017(10)
C15	0.0838(16)	0.105(3)	0.094(2)	0.0311(18)	0.0417(15)	0.0203(17)
C16	0.129(3)	0.113(3)	0.165(4)	0.075(3)	0.086(3)	0.045(3)
C17	0.113(3)	0.078(2)	0.227(5)	0.005(3)	0.109(3)	0.000(2)
C18	0.0773(18)	0.109(3)	0.167(4)	-0.058(3)	0.047(2)	-0.0090(19)
C19	0.0616(13)	0.088(2)	0.105(2)	-0.0221(17)	0.0177(13)	0.0052(13)
C20	0.0669(13)	0.0602(15)	0.0568(14)	0.0070(11)	0.0192(11)	-0.0037(11)
C21	0.1124(19)	0.0781(19)	0.0630(16)	-0.0007(15)	0.0195(15)	0.0115(15)
C22	0.167(3)	0.080(2)	0.0643(19)	-0.0052(16)	-0.001(2)	0.003(2)
C23	0.110(3)	0.106(3)	0.090(3)	0.017(2)	-0.0115(19)	-0.028(2)
C24	0.0726(16)	0.144(3)	0.079(2)	0.032(2)	0.0091(15)	-0.0156(17)
C25	0.0706(14)	0.0952(19)	0.0643(16)	0.0150(13)	0.0193(12)	-0.0002(13)
C26	0.0559(11)	0.0595(14)	0.0484(13)	-0.0049(10)	0.0084(9)	-0.0092(11)
C27	0.0695(14)	0.0652(16)	0.0727(16)	-0.0053(13)	0.0218(12)	-0.0185(13)
C28	0.0771(14)	0.084(2)	0.0741(17)	-0.0146(14)	0.0253(12)	-0.0071(15)
C29	0.0855(17)	0.0696(19)	0.0812(18)	-0.0212(14)	0.0149(14)	0.0006(14)
C30	0.0875(17)	0.0601(17)	0.0867(19)	-0.0094(14)	0.0202(14)	-0.0124(14)
C31	0.0650(12)	0.0616(16)	0.0669(14)	-0.0026(12)	0.0130(10)	-0.0136(13)

Table 9: Hydrogen atomic coordinates and isotropic atomic displacement parameters (Å2) for 11b.

	x/a	y/b	z/c	U(eq)
H2	0.3603	0.3352	0.2230	0.085
H3A	0.5026	0.4616	0.1171	0.082
H3B	0.5614	0.3513	0.1935	0.082
H3AA	0.5824	0.5933	0.2525	0.065
H4	0.7692	0.4092	0.2940	0.063
H5A	0.9055	0.4948	0.4617	0.075
H5B	0.7824	0.5776	0.4621	0.075
H6	0.7980	0.3300	0.4860	0.076
H7A	0.7753	0.2577	0.6451	0.098
H7B	0.6269	0.3058	0.6264	0.098
H8A	0.7029	0.4252	0.7827	0.203
H8B	0.8498	0.3749	0.8018	0.203
H8C	0.7333	0.2913	0.8142	0.203
H10A	1.0086	0.5898	0.2144	0.076
H10B	1.0212	0.5587	0.3378	0.076
H11	0.7780	0.5438	0.1658	0.065
H12A	0.8538	0.7329	0.1107	0.078
H12B	0.7754	0.8385	0.1457	0.078
H13A	0.9621	0.7857	0.3153	0.075
H13B	0.9067	0.7155	0.4007	0.075
H15	1.1260	0.3760	0.3898	0.109
H16	1.1744	0.1828	0.3522	0.152
H17	1.0794	0.1024	0.1804	0.153
H18	0.9397	0.2179	0.0449	0.138
H19	0.8901	0.4081	0.0821	0.103
H21	0.7309	0.6242	-0.0499	0.103
H22	0.5227	0.5574	-0.1685	0.133
H23	0.3235	0.6134	-0.1357	0.132
H24	0.3244	0.7326	0.0082	0.122
H25	0.5244	0.7942	0.1264	0.092
H27	0.6536	0.7946	0.3907	0.083
H28	0.5523	0.9630	0.4333	0.093
H29	0.6454	1.1472	0.4203	0.097
H30	0.8430	1.1600	0.3709	0.095
H31	0.9448	0.9916	0.3296	0.079
